# Resveratrol–Maltol and Resveratrol–Thiophene Hybrids as Cholinesterase Inhibitors and Antioxidants: Synthesis, Biometal Chelating Capability and Crystal Structure

**DOI:** 10.3390/molecules27196379

**Published:** 2022-09-27

**Authors:** Milena Mlakić, Lajos Fodor, Ilijana Odak, Ottó Horváth, Marija Jelena Lovrić, Danijela Barić, Valentina Milašinović, Krešimir Molčanov, Željko Marinić, Zlata Lasić, Irena Škorić

**Affiliations:** 1 Department of Organic Chemistry, Faculty of Chemical Engineering and Technology, University of Zagreb, Marulićev trg 19, HR-10000 Zagreb, Croatia; 2 Department of General and Inorganic Chemistry, Institute of Chemistry, Faculty of Engineering, University of Pannonia, P.O. Box 158, H-8201 Veszprém, Hungary; 3 Department of Chemistry, Faculty of Science and Education, University of Mostar, Matice hrvatske bb, 88000 Mostar, Bosnia and Herzegovina; 4 Group for Computational Life Sciences, Division of Physical Chemistry, Ruđer Bošković Institute, Bijenička cesta 54, HR-10000 Zagreb, Croatia; 5 Division of Physical Chemistry, Rudjer Bošković Institute, Bijenička cesta 54, HR-10000 Zagreb, Croatia; 6 NMR Center, Rudjer Bošković Institute, Bijenička cesta 54, HR-10000 Zagreb, Croatia; 7 Teva api Analytical R&D, Pliva, Prilaz Baruna Filipovića 25, HR-10000 Zagreb, Croatia

**Keywords:** cholinesterase inhibitory activity, molecular docking, pyran-4-ones, thienostilbenes

## Abstract

New resveratrol–thiophene and resveratrol–maltol hybrids were synthesized as cholinesterase inhibitors and antioxidants. As with photostability experiments, biological tests also found remarkable differences in the properties and behavior of thiophene and maltol hybrids. While resveratrol–thiophene hybrids have excellent inhibitory and antioxidant properties (similar to the activity of reference drug galantamine), maltols have been proven to be weaker inhibitors and antioxidants. The molecular docking of selected active ligands gave insight into the structures of docked enzymes. It enabled the identification of interactions between the ligand and the active site of both cholinesterases. The maltols that proved to be active cholinesterase inhibitors were able to coordinate Fe^3+^ ion, forming complexes of 1:1 composition. Their formation constants, determined by spectrophotometry, are very similar, lgK = 11.6–12.6, suggesting that Fe^3+^ binds to the common hydroxy-pyranone moiety and is hardly affected by the other aromatic part of the ligand. Accordingly, the characteristic bands in their individual absorption spectra are uniformly red-shifted relative to those of the free ligands. The crystal structures of two new resveratrol–maltol hybrids were recorded, giving additional information on the molecules’ intermolecular hydrogen bonds and packing. In this way, several functionalities of these new resveratrol hybrids were examined as a necessary approach to finding more effective drugs for complicated neurodegenerative diseases.

## 1. Introduction

**Figure 1:**
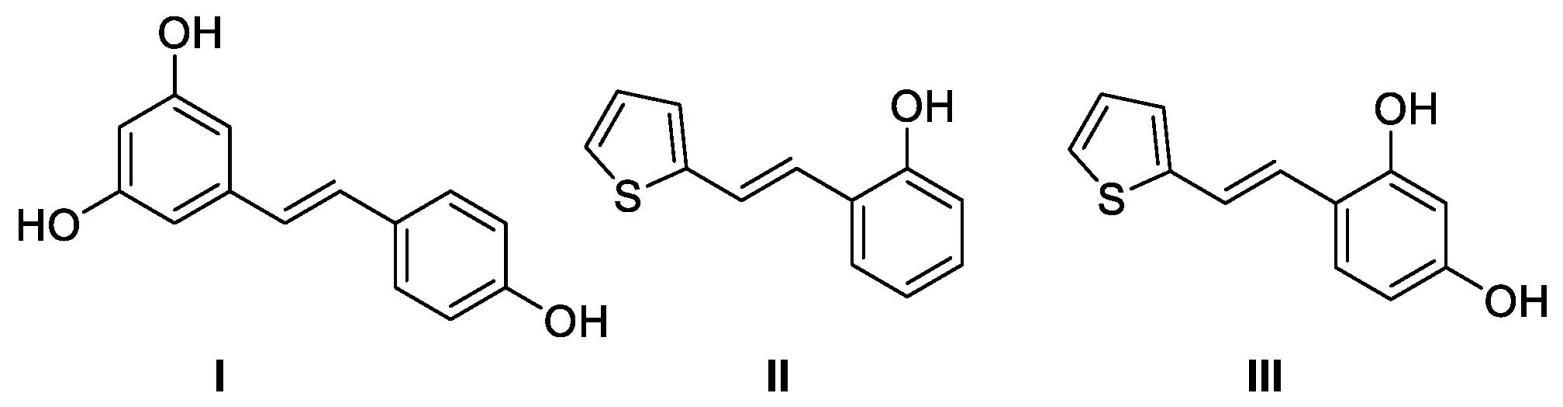
Resveratrol (**I**) and thienostilbenes (**II** and **III**) with improved antioxidant activity.

Resveratrol (3,4′,5-trihydroxystilbene (**I**), Figure 1 is a bioactive non-flavonoid polyphenol belonging to the group of *trans*-stilbene derivatives [1,2,3]. Resveratrol exists in the geometric isomeric forms as *cis*- and *trans*-isomers. The *trans*-isomer is more stable and is associated with biological properties and health benefits. It plays an important role in neurodegenerative diseases such as Alzheimer’s (AD) and Parkinson’s disease [4]. It is proven that AD is a multifactorial disease involving the aggregation of β-amyloid (Aβ) [5,6], oxidative stress [7], and metal ion dyshomeostasis [8,9].

There is limited evidence in the literature of UV light interactions with *trans*-resveratrol [10,11]. Unfortunately, the *trans*-resveratrol isomerizes to the *cis*-isomer upon light or UV irradiation [12]. *cis*-Resveratrol is less stable and cannot be utilized commercially [13]. For this reason, there is a constant search for new heterocyclic resveratrol analogues that possess the same or even better bioactive properties as *trans*-resveratrol but are less sensitive to light. Oxidative stress participates in the pathology of many neurodegenerative diseases [14,15,16] and also represents an important aspect of research. In patients with AD, the brain tissues are exposed to oxidative stress, which leads to protein damage, DNA damage, and lipid oxidation, thus resulting in the growth of AD [17].

Acetylcholinesterase (AChE) and butyrylcholinesterase (BChE) are two related enzymes which are in humans, the products of different genes but share about 54% of their amino acid sequence [18]. AChE has an essential physiological role in the body as it controls the transmission of nerve impulses in the cholinergic synapses of the central and peripheral nervous system through the hydrolysis of the neurotransmitter acetylcholine. It also has a role in neuritogenesis, cell adhesion, dopamine neuronal activation, and the formation of amyloid fibers characteristic for AD [19]. The role of BChE is not physiologically essential but it could be assigned to the detoxification of xenobiotics and the bioactivation of drugs [20]. Additionally, BChE serves as a co-regulator of cholinergic neurotransmission and is capable of catalyzing the hydrolysis of acetylcholine [21]. It was found that high BChE levels are associated with neuritic plaques and neurofibrillary tangles, the neuropathologic hallmarks of AD [22]. Therefore, both cholinesterases are pharmacologically relevant targets in neurodegenerative disorders, and today’s treatment includes cholinesterase inhibitors such as donepezil, galantamine, physostigmine, rivastigmine, etc. [23].

Hence, developing cholinesterase inhibitors with antioxidant capabilities may benefit the neurotransmitter action in AD patients [24,25]. Besides the inhibitory activity, the antioxidative activity has an additional positive pleiotropic effect in increasing both the level and duration of neurotransmitter activity. For the above reason, it is worth highlighting the results of some of our previous research [26]. Heteroaromatic resveratrol analogs with a hydroxyl group(s) (compounds **II** and **III**, Figure 1) exhibited effective radical scavenging activity. The half-maximal DPPH radical scavenging concentration (IC_50_) for derivative **II**, with OH group at the *ortho* position and *trans*-geometry of the stilbene moiety, is 158.8 μM, implying that **II** is a potent antioxidant. For comparison, the IC_50_ value for resveratrol was found to be 77.9 μM [27] and 74.0 μM [28].

The configurational change to *cis*-isomer resulted in a decrease in antioxidant activity, but still, the IC_50_ value is reached in the micromolar range (IC_50_ 782.1 μM). The same configurational effect was also observed for *cis*- and *trans*-resveratrol [29,30], indicating that the structure–activity relationship must be considered in the determination of biological activity. The introduction of an additional OH group at position 4 on the phenyl moiety increased antioxidant activity. Derivative **III** showed the best scavenging activity with an excellent IC_50_ value of 26.8 μM, three times better than resveratrol. In derivative **III**, both OH groups can be involved in resonance stabilization, resulting in better radical scavenging activity. Considering these results, compounds **II** and **III** may be good candidates as cholinesterase inhibitors with antioxidant capabilities.

**Figure 2:**
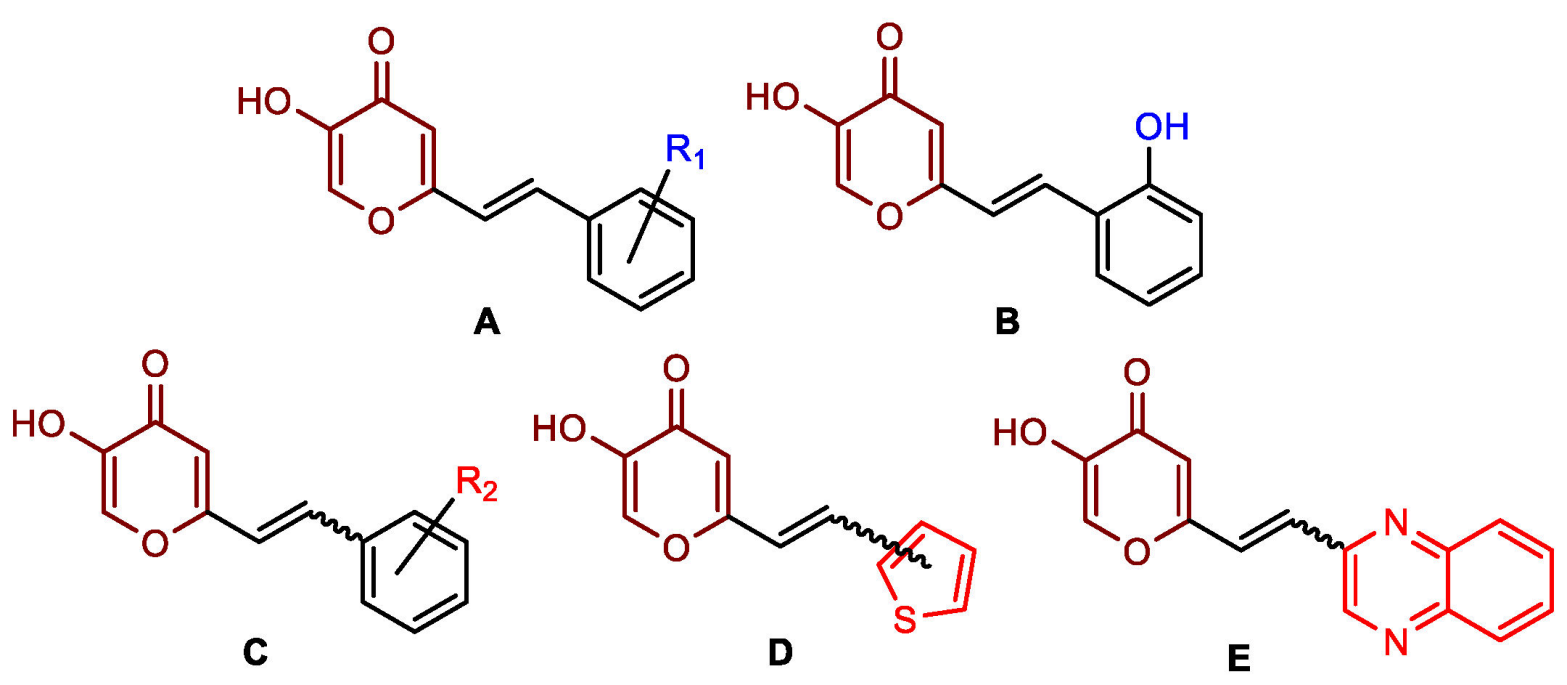
Representative resveratrol–maltol target structures.

To enhance the activity of resveratrol in the case of neurodegenerative diseases, the maltol moiety (3-hydroxypyran-4-one) was incorporated into the resveratrol-like structure to achieve resveratrol–maltol hybrids as novel multi-target-directed ligands (MTDL, structures **A** and **B**, Figure 2) and then compared with the same functionalities of thienostilbenes **II** and **III** as resveratrol derivatives (Figure 1). Among all the factors related to AD, the accumulation and aggregation of β-amyloid peptide (Aβ), the associated biometal ion dyshomeostasis, and oxidative stress have been demonstrated to be involved with the pathogenesis of AD [31,32]. There are two principal forms of Aβ in the brain tissue of AD patients, one of those responsible for the initial self-aggregation of Aβ, and the resulting β-amyloid oligomers and fibrils are toxic to neurons. High concentrations of iron ions also have been found to co-localize with amyloid deposits in AD patients compared to age-matched controls. In addition, the redox active iron ions can generate reactive oxygen species (ROS), which will cause oxidative damage and may trigger the death of neurons. The combination of metal chelation and anti-oxidant activity into one molecule may provide a promising therapeutic strategy for AD therapy. The in vitro biological evaluation of this type of compound (resveratrol–maltol hybrids) has shown their triple function: the inhibition of self-induced Aβ aggregation, antioxidation, and metal chelating activity [32]. Encouraged by this scientific knowledge, and having in mind our experience in the synthesis of (hetero)stilbene derivatives as cholinesterase inhibitors, we decided to prepare new resveratrol–maltol hybrid types combining the maltol moiety with (hetero)aryl one (structures **C**–**E**, Figure 2).

As photostability is one of the target characteristics of such resveratrol derivatives, the aim of this study was to investigate changes in UV spectra of compounds when exposed to UV light, their antioxidant and inhibitory activity against cholinesterases, as well as the capability to complex with biometals. In this way, several functionalities of these new resveratrol–maltol hybrids, as well as resveratrol–thiophene hybrids with previously improved antioxidative activity, were examined as a necessary approach to finding more effective drugs for complicated neurodegenerative diseases. As shown in our previous studies, sulfur analogs were found to be active in cholinesterase inhibition [33,34] and antioxidant activity [26]; therefore, some diheterostilbenes (maltol-heteroaromatic resveratrol analogs) are prepared in this work too (structures **D** and **E**, Figure 2).

## 2. Results and Discussion

### 2.1. Synthesis and Characterization of Resveratrol–Thiophene ***II*** and ***III*** and Resveratrol–Maltol Hybrids ***1***–***8***

**Scheme 1:**
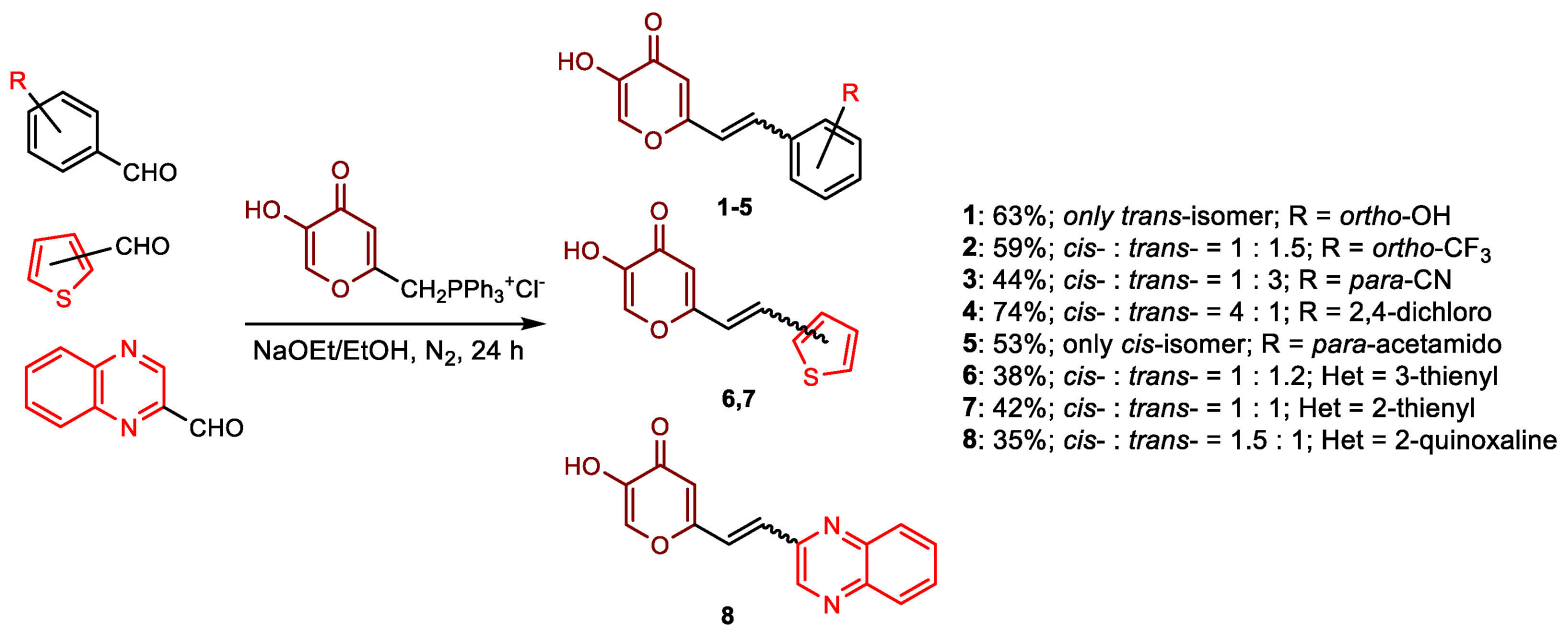
Synthesis of new 5-hydroxy-2-styryl-4*H*-pyran-4-ones **1**–**8** as chromatographically separated *cis*− and *trans*−isomers.

The synthesis of the resveratrol–thiophene hybrids **II** and **III** (Figure 1) is presented in our previous research [26], together with their spectroscopic characterization and photostability. New 5-hydroxy-2-styryl-4H-pyran-4-ones **1**–**8** (Figure 2 and Scheme 1) were also prepared by the Wittig reaction from phosphonium chloride and the selected aryl- and heteroaryl-carbaldehydes. Wittig reaction provided the styryl-pyranones **1**–**8** as mixtures of *cis*- and *trans*-isomers (except **1** and **5,** where only one isomer was formed; Scheme 1). Mixtures of the geometrical isomers of **1**–**8** were separated to obtain pure compounds by repeated column and thin-layer chromatography. According to ^1^H NMR spectroscopy, the substituent on the aryl ring strongly directs the ratio of geometric isomers in the Wittig reaction (Scheme 1). In the styryl-pyranone **1** with an *ortho*-OH substituent on the aromatic ring, trans-isomer predominates; however, in compound **5**, corresponding cis-configuration is the only product in the reaction mixture (See also Experimental Section).

All isolated isomers of styryl-pyranones **1**–**8** are fully spectroscopically characterized (See Experimental Section and ESI). In the ^1^H NMR spectra of isolated geometrical isomers of **1**–**8** (Experimental Section and ESI), visible are the resolved patterns for ethylenic protons between 4.73 and 7.14 ppm (*cis*-isomers) and between 6.50 and 7.78 ppm (*trans*-isomers) with the characteristic coupling constants, signals for the protons on various substituents and the characteristic signals and coupling constants of protons on the thienyl and quinoxaline rings.

**Figure 3:**
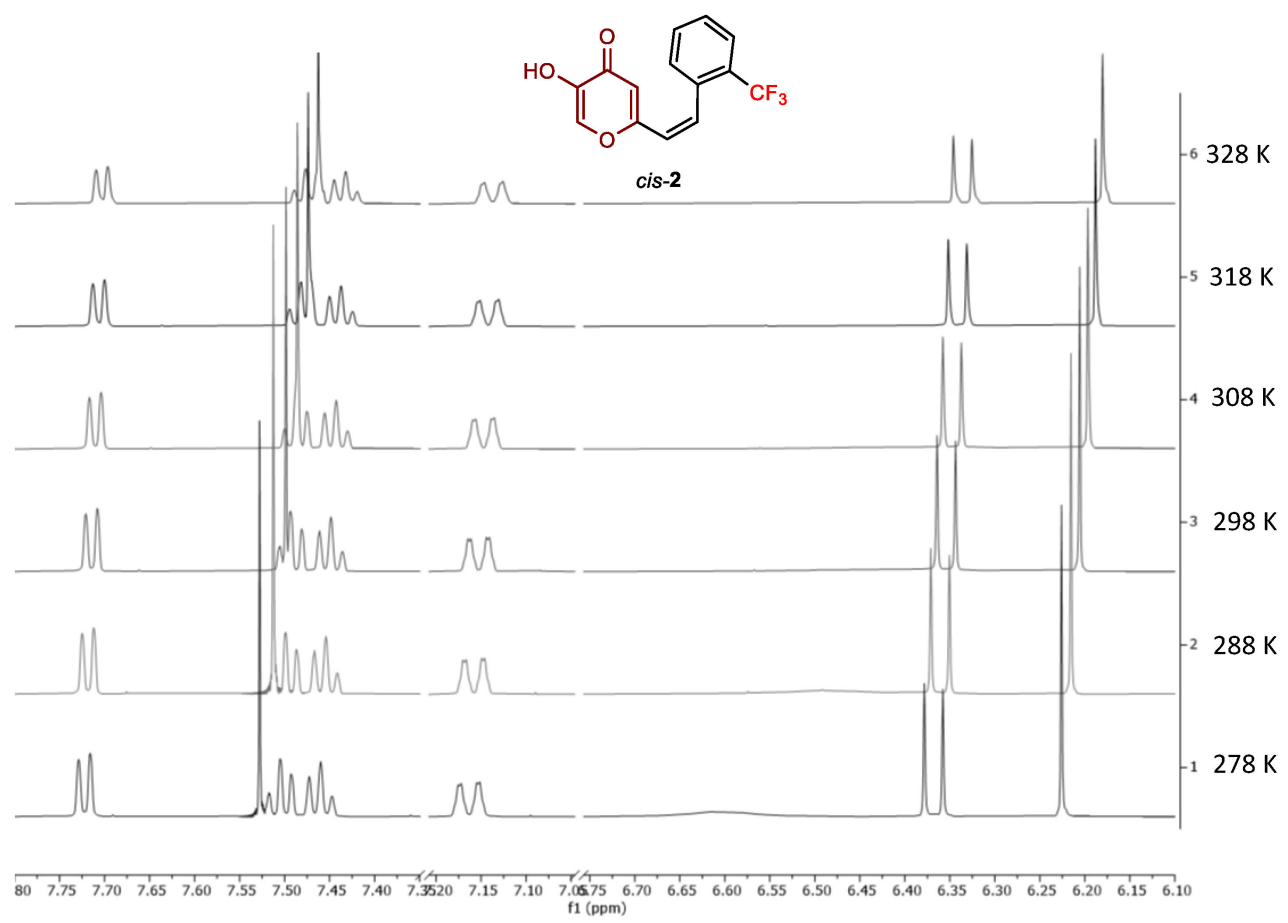
Temperature dependence of the chemical shifts of characteristic protons in the NMR experiments for *cis*-**2**.

Styryl-pyranones **2** and **6** were additionally exposed to temperature changes from 278 to 328 K. At each temperature, the corresponding ^1^H NMR spectrum was recorded (Figure 3 and ESI) to examine possible changes in conformations due to the breaking of hydrogen bonds in the molecules. Only one proton of the double bond in *cis*-**2**, located at a lower chemical shift (at 6.30 ppm), moves towards a more shielded area with the temperature increase (Figure 3). There is also a noticeable shift of both protons (at 6.25 and 7.55 ppm) of the 4-pyranone ring towards the more shielded area and the hydroxyl proton due to breaking hydrogen bonds and conformational changes.

**Figure 4:**
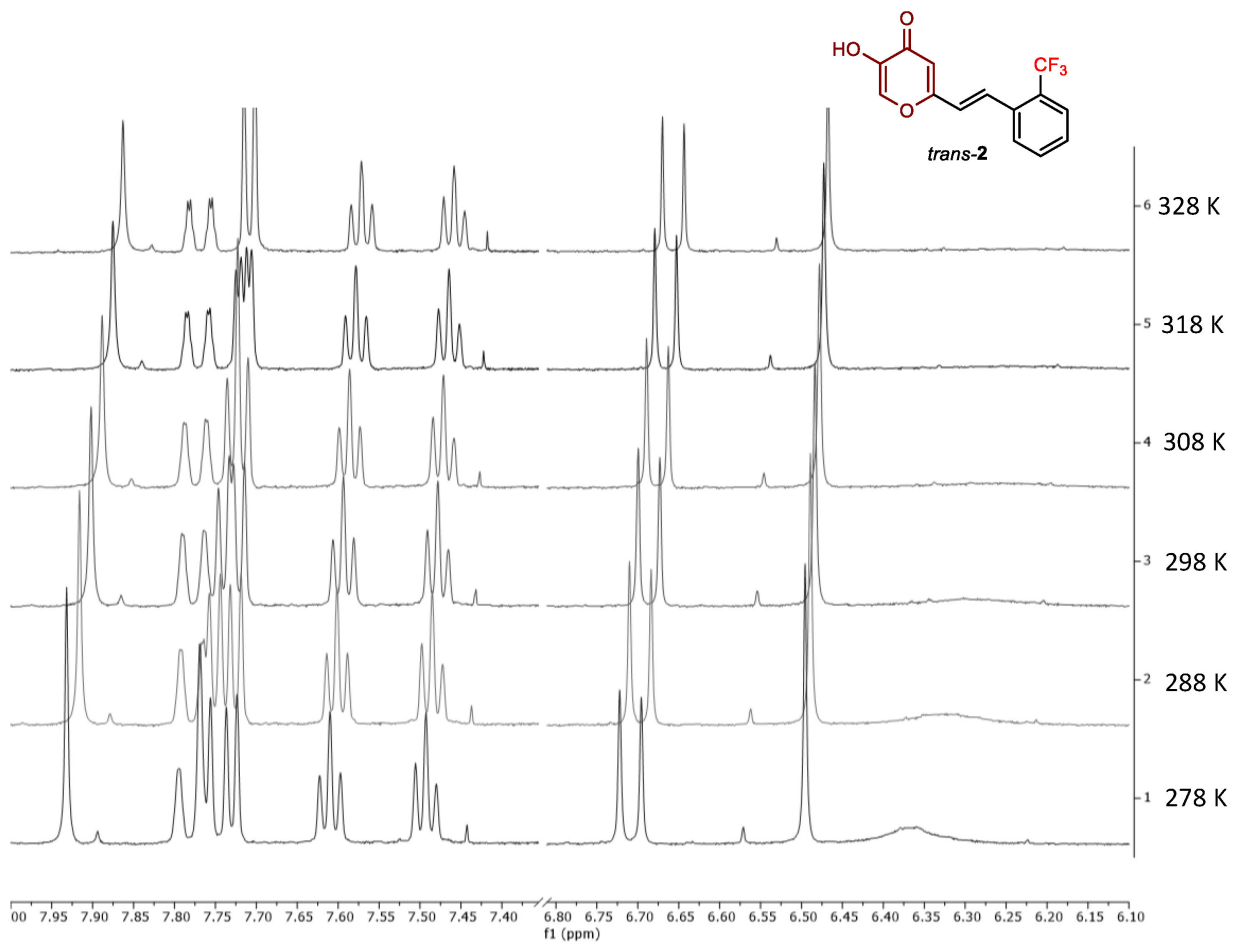
Temperature dependence of the chemical shifts of characteristic protons in the NMR experiments for *trans*-**2**.

In the case of *trans*-**2** (Figure 4), a similar trend is visible for the same group of protons. Besides that, the transition of the signals from two aromatic doublets (at 7.72 and 7.61 ppm) to one doublet at 7.61 ppm, due to the increase in temperature, is interesting. It can be explained as a consequence of the increase in kinetic energy and rotation of the single bonds, so two aromatic protons become chemically equivalent.

This kind of examination of the change in chemical shifts under the temperature influence clearly shows that there are intermolecular hydrogen bonds between the molecules of styrylpyranones in the solutions, which break and the molecules become more flexible, taking some other conformations, which is reflected in the chemical shifts changes of individual protons that are closest to the particular changes.

### 2.2. Photochemical Reactivity and Photophysical Properties of New Styryl-Pyranones ***1***–***8***

**Figure 5:**
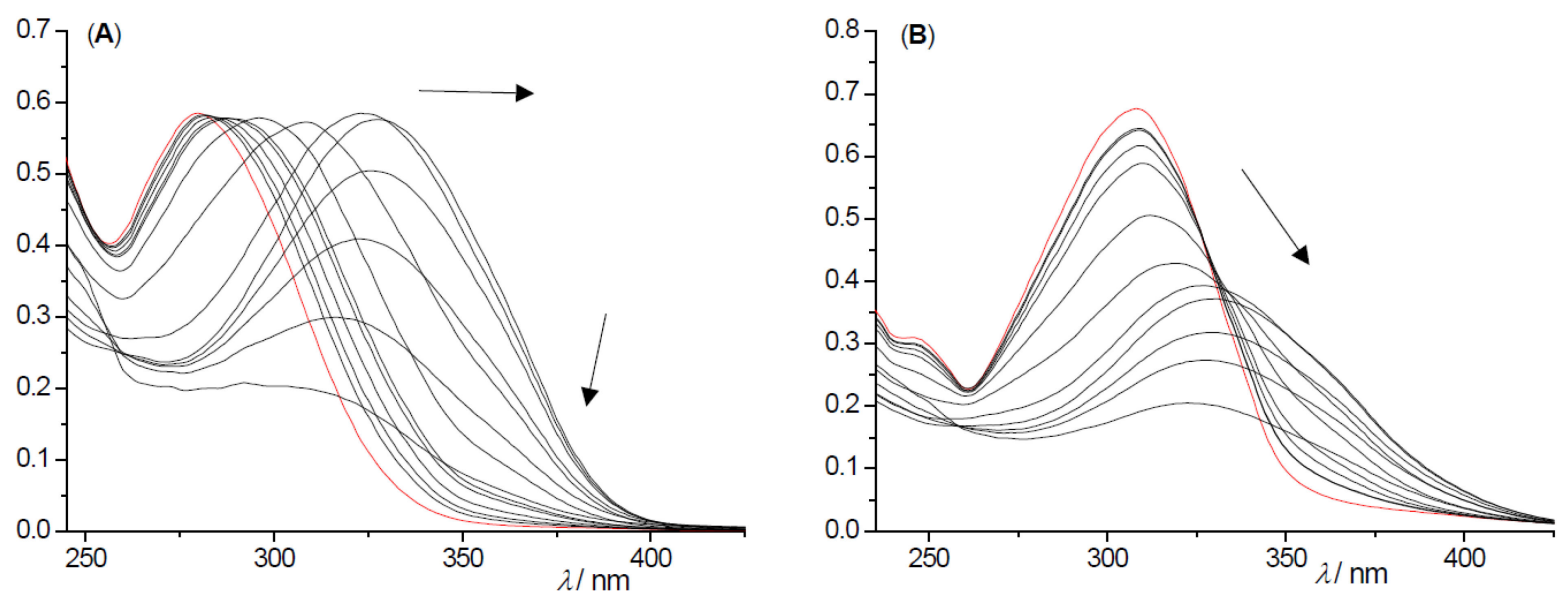
Spectral changes during the irradiation of *cis*-**2** (**A**) in ACN after 0 (red), 2, 4, 8, 16, 32, 64, 128, 256, 512, 1024, 2048 and 4096 s; (*λ*_ir_ = 313 nm, *l* = 1 cm) and *trans*-**2** (**B**) in ACN after 0 (red), 2, 4, 8, 16, 32, 64, 128, 256, 512, 1024 and 2048 s; (*λ*_ir_ = 313 nm, *l* = 1 cm).

**Figure 6:**
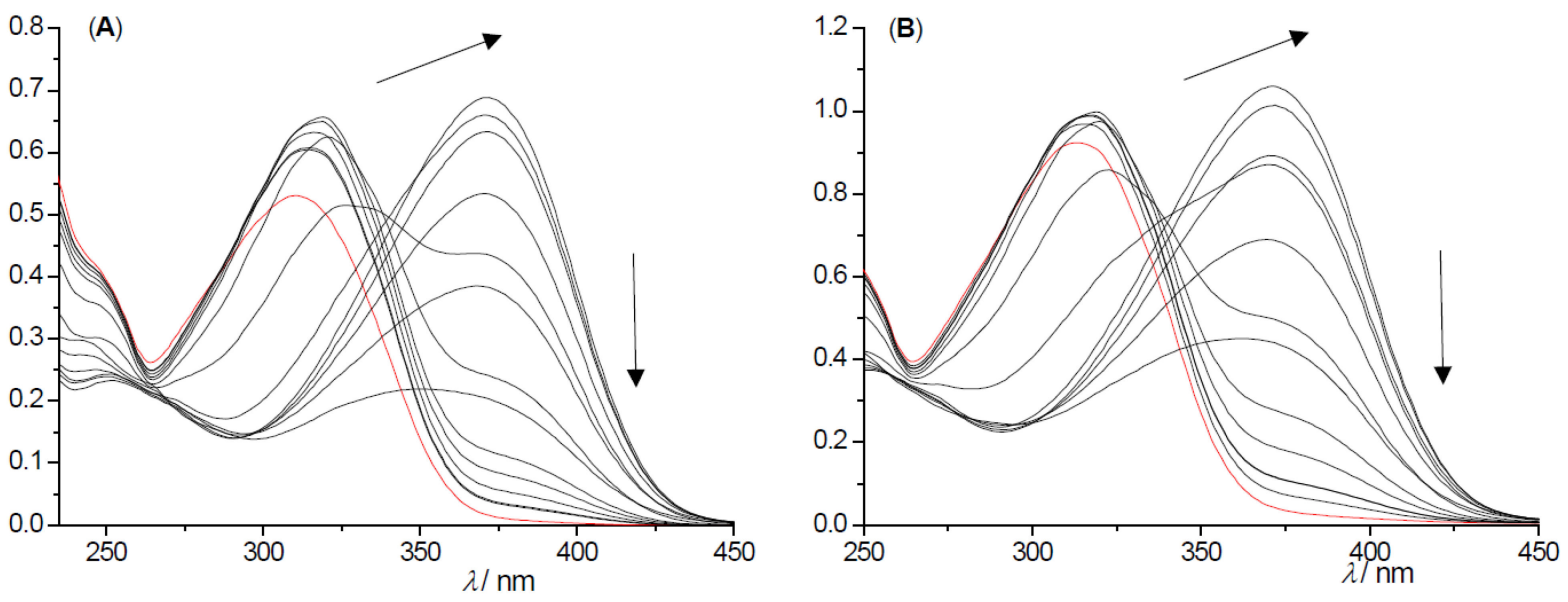
Spectral changes during the irradiation of *cis*-**6** (**A**) in ACN after 0 (red), 2, 4, 8, 16, 32, 64, 128, 256, 512, 1024, 2048 and 4096 s; (*λ*_ir_ = 313 nm, *l* = 1 cm) and *trans*-**6** (**B**) in ACN after 0 (red), 2, 4, 8, 16, 32, 64, 128, 256, 512, 1024 and 2048 s; (*λ*_ir_ = 313 nm, *l* = 1 cm).

To explore the photochemical reactivity of the new resveratrol–maltol hybrids **1**–**8**, we conducted irradiations at 313 nm of representative compounds **2** and **6** in ACN, and the course of the reaction was followed by UV-Vis spectroscopy (Figure 5 and Figure 6). Upon irradiation of the *cis*-isomer of compound **2**, the bathochromic shift was observed, without a clear isosbestic point in the spectrum (Figure 5), in the beginning, in agreement with the formation of the *trans*-isomer. Contrary to resveratrol–thiophene hybrids **II** and **III** [26], in the continuation of irradiation, the further bathochromic shift is noticed toward values of absorbance wavelengths higher than those of the *trans*-isomer, which can be attributed to secondary processes such as ring closure or phototransposition [35]. The mechanism behind these changes could be the subject of some future mechanistic computational study. It is evident that the photostability of resveratrol–maltol hybrids significantly differs from those of resveratrol–thiophene hybrids [26]. Further, the *trans*-**2** isomer upon irradiation shows the unusual isosbestic point at 235 nm and bathochromic shift besides the hypochromic one, with the same explanation of further secondary processes, such as ring closure or phototransposition (Figure 5 and Figure 6). So, for *cis*-**2,** *Z*–*E* photoisomerization is the primary photochemical reaction, but not for *trans*-**2**.

In the case of compound **6** (Figure 6), irradiating the *cis*-isomer at 313 nm again resulted in the bathochromic shift to higher wavelengths than those observed for the *trans*-isomer, but followed by the hyperchromic one as the result of the primary *Z*–*E* photoisomerization, and secondary ring closure or phototransposition process. Upon the irradiation of *trans*-**6**, the trends are very similar to those of *cis*-**6**, only without the primary *E*–*Z* photoisomerization process and clear isosbestic point compared with *trans*-**2**.

**Figure 7:**
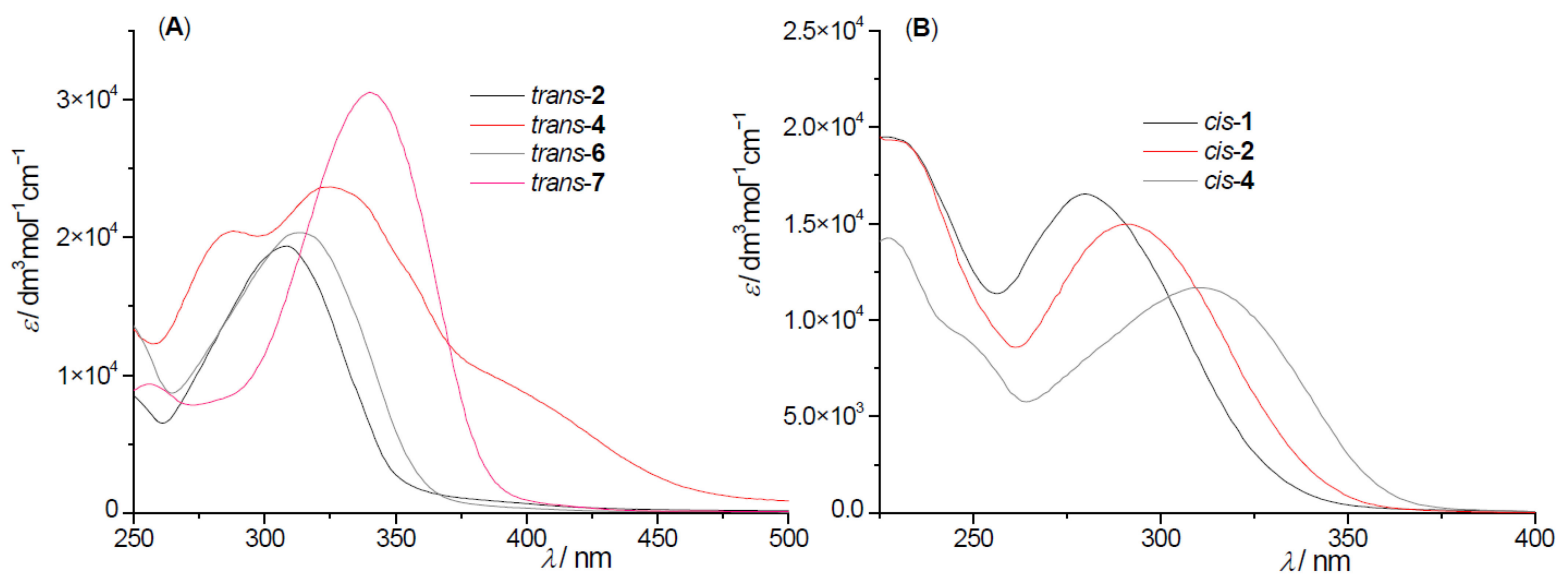
UV spectra (ACN) of representative *trans*− (**A**) and *cis*−isomers (**B**) of new resveratrol–maltol hybrids.

UV-Vis spectra of *cis*- and *trans*-isomers of **1**–**8** in ACN (Figure 7 and Experimental section) are typical for diarylethene [36,37] with the strong absorption with a maximum at about 300 nm, respectively, for the *trans*-isomers, and less intense and hypsochromically shifted bands for the *cis*-isomers (Figure 7 and Experimental section). These absorption bands correspond to the fully symmetry allowed HOMO → LUMO transition.

### 2.3. Cholinesterases Inhibitory and Antioxidative Activity of Resveratrol–Maltol and Resveratrol–Thiophene Hybrids

In continuation of the photostability testing and evidence regarding the difference between the resveratrol–maltol and resveratrol–thiophene hybrids in this sense, we tested and compared all of them (**II**, *cis*-**II**, **III,** and **1**–**8**) as reversible inhibitors of acetylcholinesterase (AChE) and butyrylcholinesterase (BChE) by Ellman et al. [38] modified method in a wide range of concentration to evaluate the inhibitor concentration that inhibits 50% of enzyme activity (IC_50_). In addition, antioxidant activity tests of the compounds have been performed using DPPH radical scavenging assay [39] to see whether they can possess both inhibitory and antioxidant activity. The half-maximal DPPH radical scavenging concentration (IC_50_) was calculated where applicable, and the results for all active target compounds are summarized in Table 1. Of the 12 tested resveratrol–maltol hybrids, only four showed inhibitory potency, while the rest did not show any inhibitory or antioxidation activity.

As with photochemical experiments, biological tests also found remarkable differences in the properties and behavior of thiophene and maltol hybrids. While resveratrol–thiophene hybrids have excellent inhibitory and antioxidant properties, maltols have been proven to be weak inhibitors and even weaker antioxidants.

The results of biological tests for resveratrol–thiophene derivatives showed the great potential of these compounds. The antioxidant effect was previously tested and compared with resveratrol [26] which showed IC_50_ values of 77.9 μM [27] and 74.0 μM [28]. These derivatives showed antioxidant activity, with **III** being the most potent antioxidant (IC_50_ = 26.8 μM) due to the presence of two OH groups. It has been revealed now that in addition to their antioxidant activity, they also have excellent inhibitory activity on both enzymes. Among the thiophene hybrids, **II** showed the highest inhibitory activity against BChE (IC_50_ = 4.6 μM), which is similar to the activity of reference drug galantamine used as a therapeutic drug for AD (IC_50_ = 7.9 μM). Derivative **III** also showed great inhibitory activity toward BChE (IC_50_ = 5.3 μM), which is still slightly better than galantamine. These derivatives performed as better potential inhibitors of BChE than AChE, where they achieved somewhat lower IC_50_ values but still in a good range of concentrations (IC_50_ = 15.7 and 46.6 μM, respectively). The combination of thiophene and phenol in *trans* geometry has proven to be convenient for biological properties. The earlier observation that the *trans* geometry is more favorable for bioactivity [29] was confirmed by examining the *cis*-isomer of compound **II**. A change in geometry to *cis*-**II** drastically decreased inhibitory activities toward AChE (IC_50_ > 500 μM), while the result for BChE was still in a very good range (IC_50_ = 18.9 μM). The configurational change was also followed by a reduction in antioxidant potency.

Of the 12 tested resveratrol–maltol hybrids, only 4 shoμwed inhibitory potency, while the rest did not show any inhibitory or antioxidation activity. These four derivatives are active only toward the BChE enzyme, with IC_50_ values at relatively high concentrations compared to the standard (Table 1). Antioxidant activity was recorded only for the *trans*-**1** derivative possessing a phenolic ring, while none of the other derivatives captured the radical (IC_50_ >> 1000 μM). Therefore, the insertion of maltol into the resveratrol structure drastically reduces inhibition potency and antioxidant activity.

Furthermore, we can compare the structure and bioactivity of the three hybrids that possess a thiophene ring with stilbene conjunction at position 2 (**II**, **III,** and **7**). While **II** and **III** showed great results, hybrid **7** completely missed any tested bioactivity. Therefore, replacing phenol with maltol completely extinguishes the biological activity of interest in this research. It is important to note that the 3-thienyl-maltol combination in the trans geometry showed some inhibitory activity toward BChE, so the position of the thiophene substitution was also crucial. Concerning these experimental results, for the resveratrol–maltol hybrids **1**–**8,** another type of biological activity should be proposed, which will be more specific to them. Although it has been shown in previous research that resveratrol–maltol hybrids show inhibition of self-induced Aβ aggregation (which is connected with AD), antioxidation, and metal chelating activity [32], the cholinesterase inhibitory activity is not as specific for them as for the resveratrol–thiophene hybrids **II** and **III**.

### 2.4. Computational Study of Resveratrol–Thiophene and Resveratrol–Maltol Hybrids as Cholinesterase Inhibitors

**Figure 8:**
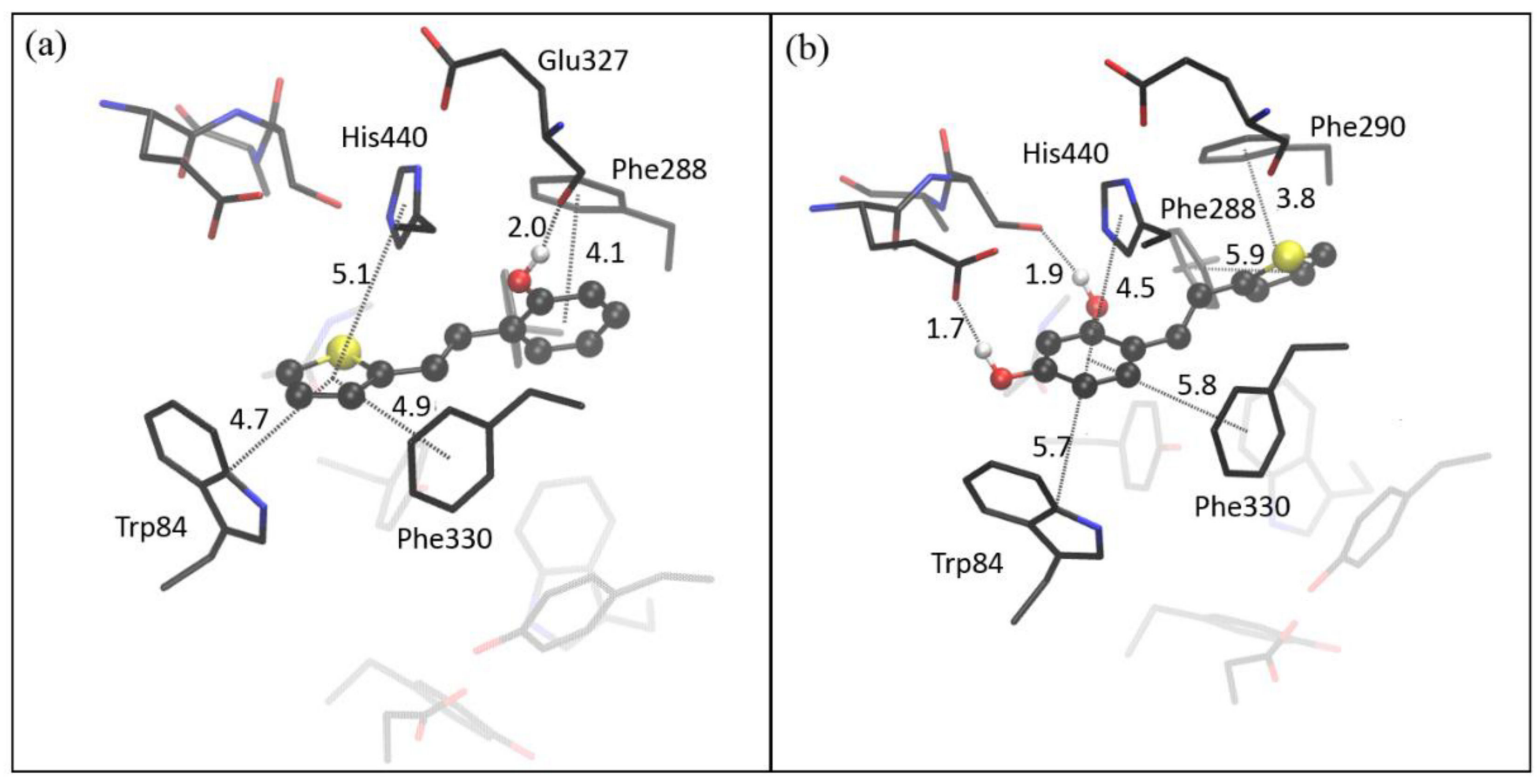
The structure of the active site of AChE docked with **II** (**a**) and **III** (**b**). Hydrogens omitted for the sake of clarity.

According to Table 1, both *trans*-isomers of resveratrol–thiophene derivatives, **II** and **III**, showed excellent inhibitory activity toward cholinesterases. Molecular docking was performed to visualize the structure and gain insight into stabilizing interactions in the complex between the tested molecule and the enzyme’s active site. The molecular structures of the most stable complexes of **II** and **III** with the active site of AChE, respectively, are shown in Figure 8.

Molecules **II** and **III** possess two aromatic cores, phenyl and thienyl, that can participate in stabilizing π−π stacking with aromatic residues of the AChE active site. According to the clustering histogram, ligand poses obtained by docking of **II** are grouped into two conformational clusters: one contains 8, and the second is populated with 17 poses (See ESI, Table S1). The structure presented in Figure 8a is the most favorably oriented binding conformation from the more populated cluster. The orientation of **II** enables T-shaped π−π stacking between thienyl and two residues of the anionic site (AS), Trp84 and Phe330, at distances of 4.7 and 4.9 Å from thienyl (measured from the centers of the rings). The phenyl unit of **II**, situated close to the acyl pocket of the active site, is engaged in π−π stacking with Phe288 at a distance of 4.1 Å.

The docking of **III** also results in two clusters of ligand poses; however, the more stable one is predominantly populated (24 poses), while the second cluster contains only one ligand pose. In contrast to the orientation of **II**, molecule **III** is docked so that its phenyl core faces the AS, and thienyl is engaged in stacking with residues belonging to the acyl pocket, Phe288 and Phe290, at distances of 3.8 and 5.9 Å, respectively (Figure 8b). Additional π−π stacking between thienyl of **II** and His440 (5.1 Å) and phenyl of **III** and His440 (4.5 Å) is observed. Resveratrol–thiophene derivatives have hydroxyl group(s) at phenyl, able to form hydrogen bonds (HB) within the active site. Compound **II** possesses one -OH group at phenyl; it forms HB with a carbonyl group of Glu327 that belongs to the esteratic subsite (Ser200, His440, Glu327). In the active site of AChE docked with **III**, one hydroxyl group of **III** makes the hydrogen bond with Glu199 (part of the anionic site), while the second -OH is approaching the Ser200, at the distance of 1.9 Å from serine oxygen.

**Figure 9:**
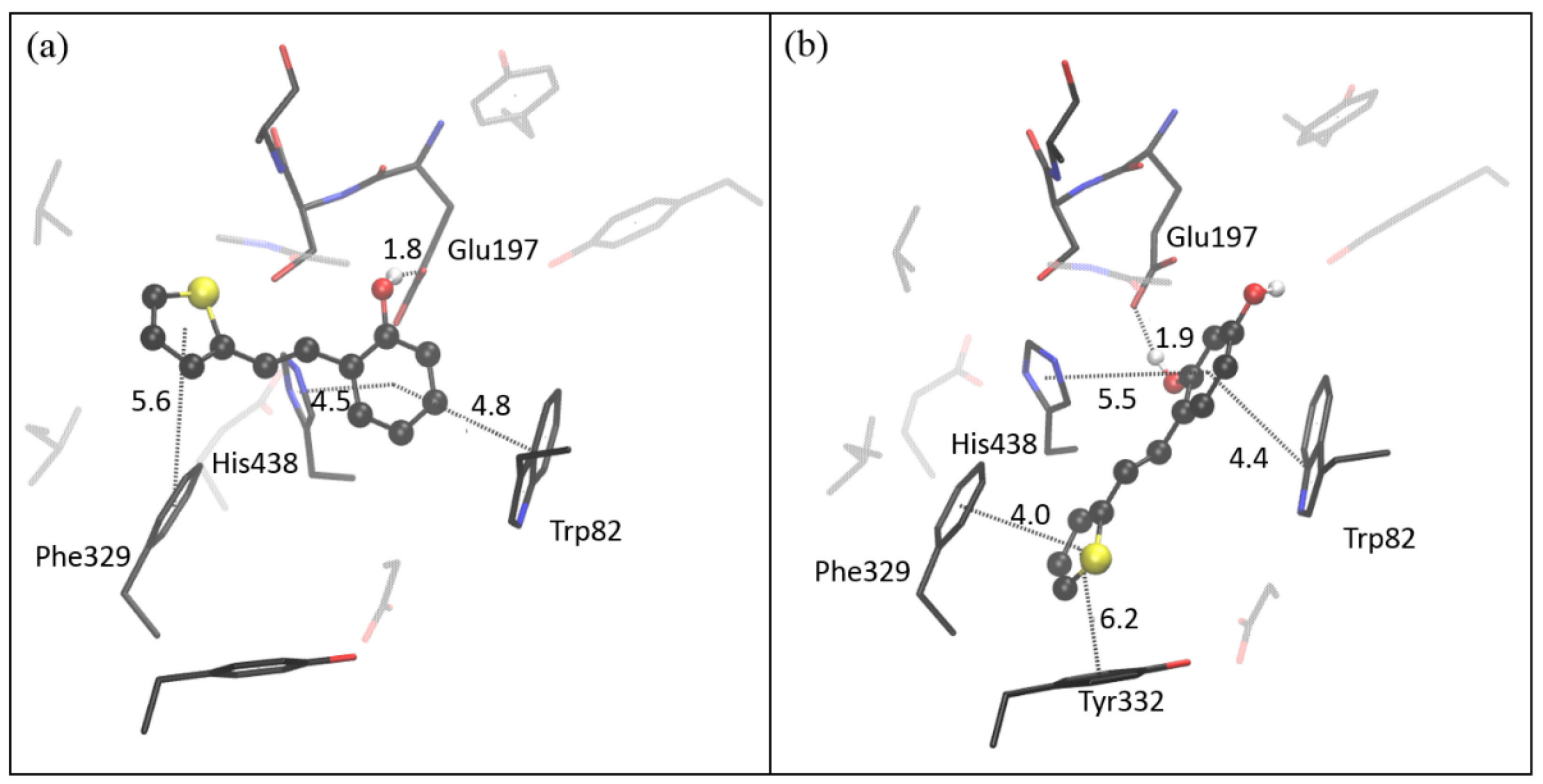
The structure of the active site of BChE docked with **II** (**a**) and **III** (**b**). Hydrogens omitted for clarity.

The molecular docking of **II** and **III** into the active site of BChE again revealed the presence of π−π interactions between ligand and aromatic residues of the enzyme. It is visible from Figure 9 that thienyl in both compounds interacts with Phe329 of the anionic site, although the orientation of the two ligands is not the same. In the structure docked with **III**, thienyl is additionally stabilized by the π−π stacking with Tyr332 (belonging to the peripheric anionic site, PAS). In contrast, **II** entered the active site deeper than **III**; thus, the interaction with PAS is not established in the case of **II**.

The phenyl core of both ligands is engaged in the π−π stacking with His438 (esteratic subsite) and Trp82 of AS. One hydrogen bond is formed between the -OH group and Glu197 in the active site of BChE docked with **II**. Although molecule **III** has two hydroxyl groups, the most favorable conformation obtained by docking results in only one H-bond; again, this is with Glu327.

**Figure 10:**
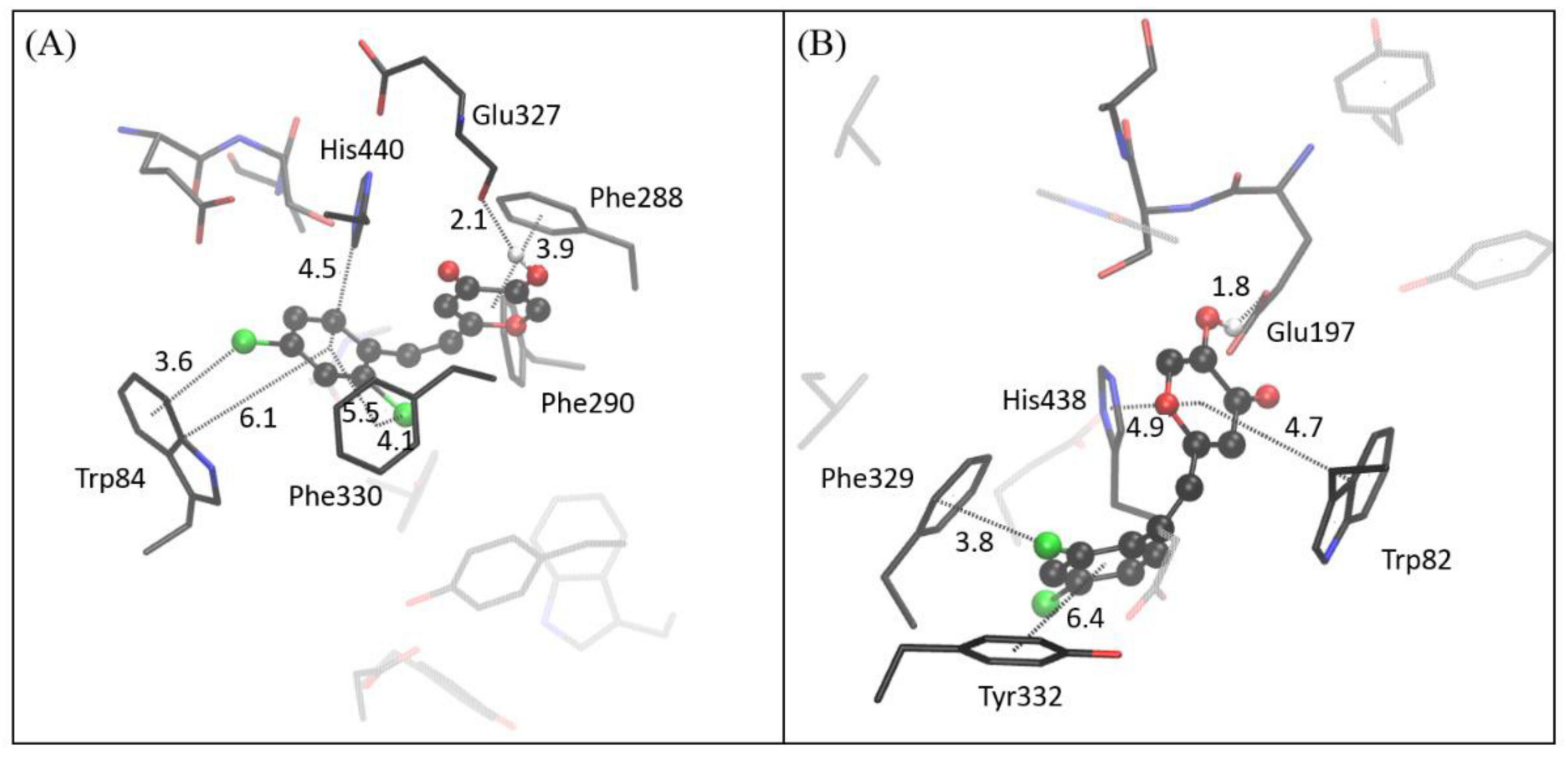
The complex obtained by docking *cis*-**4** into the active site of AChE (**A**), and by docking *cis*-**4** into the active site of BChE (**B**). Hydrogens omitted for clarity.

Among resveratrol maltol hybrids, the only compound that shows inhibitory activity toward both cholinesterases is molecule *cis*-**4** (Table 1). The most stable complexes obtained by docking *cis*-**4** into the active site of AChE and BChE, respectively, are presented in Figure 10. In the complex of *cis*-**4** docked into AChE, the π−π stacking of dichloro-substituted phenyl with residues in the anionic site, Phe330 and Trp84, occurs. In the BChE, dichloro-phenyl of the ligand interacts only with the Phe329 of the AS but also with the residue in the PAS, Tyr332. The maltol fragment is involved in π−π stacking with the residues of the acyl pocket of the AChE (Phe288, Phe190), whereas in the BChE, there is an interaction between maltol and Trp82. In AChE, histidine in the esteratic subsite (His440) interacts with the dichloro-phenyl fragment of the *cis*-**4**. In contrast, His438 from the esteratic site of BChE is situated at 4.9 Å from the maltol fragment of the ligand.

The hydrogen bond in AChE is formed between the -OH group of maltol and Glu327, while in BChE, this hydroxyl forms a hydrogen bond with Glu197. The stabilizing dispersive attraction is observed between chlorine atoms and aromatic residues: in the AChE, *para*-Cl is placed at the distance of 3.6 Å from the center of the benzene ring of Trp84 and 3.5 Å from the closest C atom of the same residue, with the dihedral angle between the chlorine atom and the plane of the benzene ring being ~80. When the difference between these two values is equal to or less than 0.3 Å, the Cl-π interaction is defined as “face-on” [40]. Similarly, the second chlorine atom at the *ortho* position of the dichloro-phenyl of *cis*-**4** is also involved in face-on Cl-π interaction with another aromatic residue in the active site, Phe330. In contrast, *ortho*-chlorine in the complex of *cis*-**4** with BChE satisfies the criteria to “edge on” Cl-π interaction with the Tyr332, where chlorine is placed at distances of 4.2 and 3.5 Å to the ring center and the closest carbon, respectively. The position of *para*-chlorine of *cis*-**4** docked into BChE enables edge-on Cl-π interaction with the residue Phe329: the center of the phenylalanine ring is placed at 4.2 Å from this chlorine atom, while the distance between the Cl and the nearest C atom of the phenylalanine ring is 3.6 Å.

**Figure 11:**
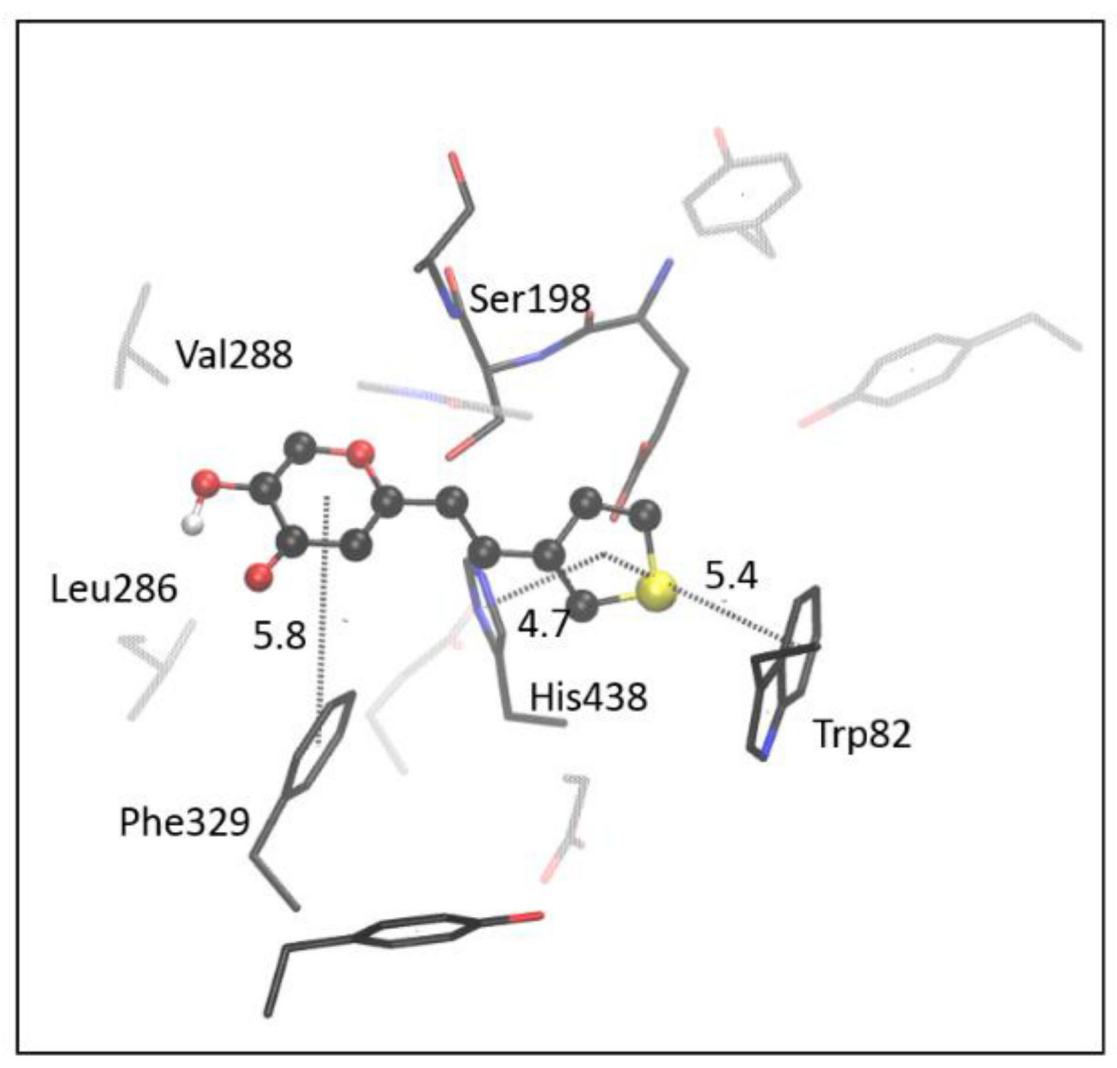
The complex obtained by docking *trans*-**6** into the active site of BChE. Hydrogens omitted for clarity.

Except for *cis*-**4**, tested resveratrol maltol hybrids did not show inhibitory activity toward AChE. However, the measurement of their activity toward BChE showed moderate inhibitory potential. The best result was achieved with compound *trans*-**6**, so we performed molecular docking to identify interactions between the ligand and the active site. Figure 11 shows the most favorable structure obtained by the molecular docking of *trans*-**6** to the active site of BChE. Interestingly, all ligand poses belong to one conformational cluster.

The thienyl fragment participates in T-shaped π-stacking with Trp82; on the other side of the ring plane, it interacts with His438. Maltol is placed in the acyl pocket (Leu286, Val288). The conformation and placement of *trans*-**6** seem to block the esteratic subsite; the double CC bond (connecting maltol and thienyl) is situated at ~3 Å from the oxygen of Ser198, thus preventing the substrate from approaching it.

The docking study has been performed without taking molecules of water into account. However, given the electronic structure of ligands and their ability to form H-bonds, it could be of interest to include relevant water molecules in the proximity of the active site of both enzymes [41,42]. Therefore, we also performed the molecular docking of tested ligands into AChE and BChE, whose active sites contain water molecules that could influence the accommodation of ligands. The results, presented in detail in Supplementary Materials, show that in AChE that includes water, compounds **II** and **III** take almost the same orientation as when waters were not present. However, the phenyl core of **II** is rotated in a way that enables interaction of its OH group with HOH611 and HOH616 (Figure S63c). In contrast, hydroxyl groups of **III** prefer the interaction with Glu199 and Ser200 (Figure S63d). Docking of **II** and **III** into the BChE in the presence of five structural water molecules result in poses that are very similar to those obtained without water; however, HOH765 interacts with the OH group of maltol fragment of **III**, being placed at the distance of 2.2 Å (Figure S64d). The docking of di-chlorinated molecule *cis*-**4** in the active site of AChE and BChE in the presence of the relevant water molecules results in ligand conformations that are similar to those obtained without water; the only interaction with water can be identified between HOH715 in BChE and carbonyl oxygen at maltol (Figure S65D). Finally, compound *trans*-**6** docked in the active site of BChE containing structural waters took the opposite orientation compared to BChE without water, which resulted in the formation of H-bond between carbonyl O of maltol and HOH715 (Figure S66b).

Although the docking study provides the approximate conformations of the ligands, these results offer insight into possibilities for main interactions between the new ligands and the enzymes, helping to rationalize inhibitor activities observed by the experiment. Both enzymes accommodate tested ligands mainly through π–π stacking interactions.

### 2.5. Biometal Chelating Capability of Cholinesterase Inhibitory Active Pyranones

In the case of the resveratrol–maltol hybrids proven to demonstrate cholinesterase inhibitory activity, it was reasonable to investigate the complex formation with a potential biometal ion such as Fe^3+^. Hence, the following compounds as ligands were studied in this respect: *trans*-**1**, *cis*-**2**, *cis*-**4**, and *trans*-**6**. Since Fe^3+^ ions strongly hydrolyze, acidic solutions were applied for the spectrophotometric titrations to determine the complex formation constants. Both types of titrations were carried out: increasing the iron(III) concentration at constant ligand concentration and increasing ligand concentration at constant C_Fe(III)_. The previous type of titration proved to be more informative, causing a change in the spectral characteristics, while in the latter case, the increasing absorbance of the ligand was dominant in the whole spectrum.

**Figure 12:**
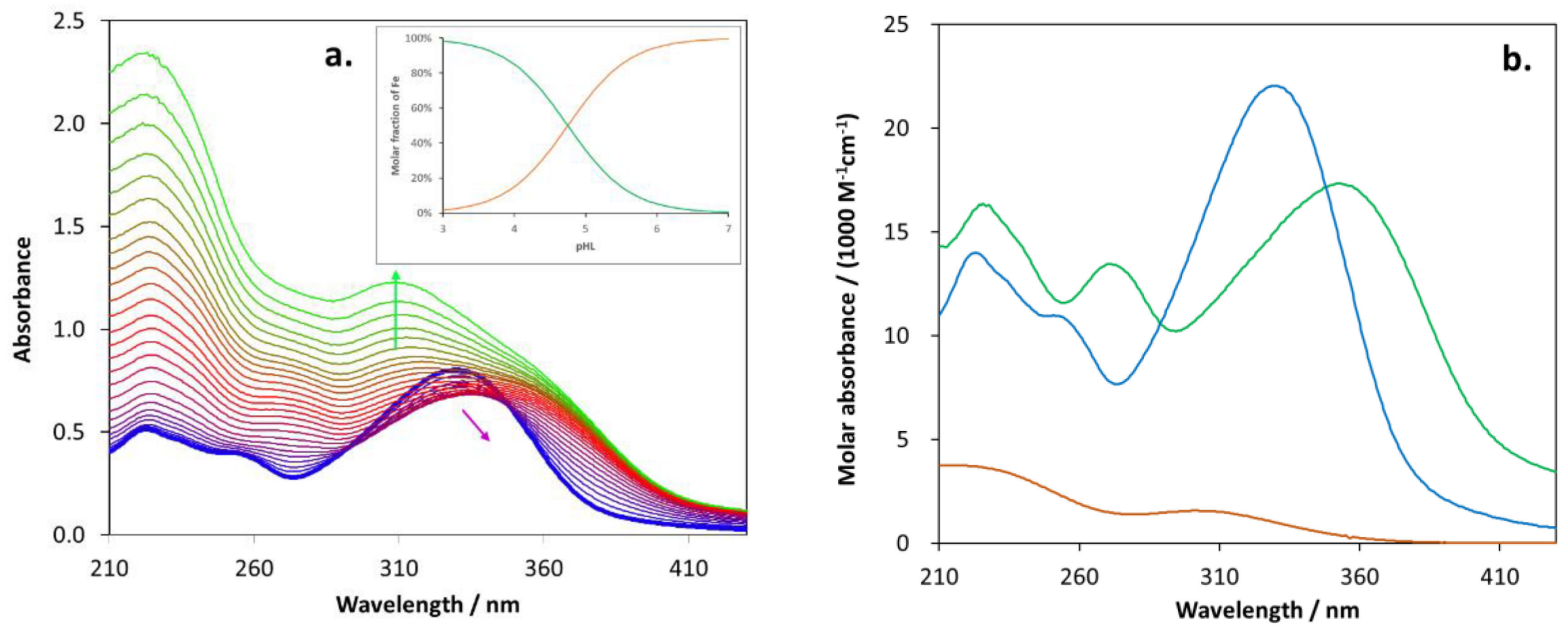
The measured absorption spectra (**a**) at constant total ligand (*trans*-**6**) concentration (3.5·10^−5^ M) and various Fe^3+^ total concentration (from 0 M (blue) to 4.7·10^−4^ M (green)). The inset shows the molar fraction of free Fe^3+^ (orange) and 1:1 complex (FeL^2+^) (green) as functions of the protonated ligand concentration (pHL). Subfigure (**b**) shows the molar absorbance of Fe^3+^ (orange), ligand (blue), and FeL^2+^ adduct (green).

**Figure 13:**
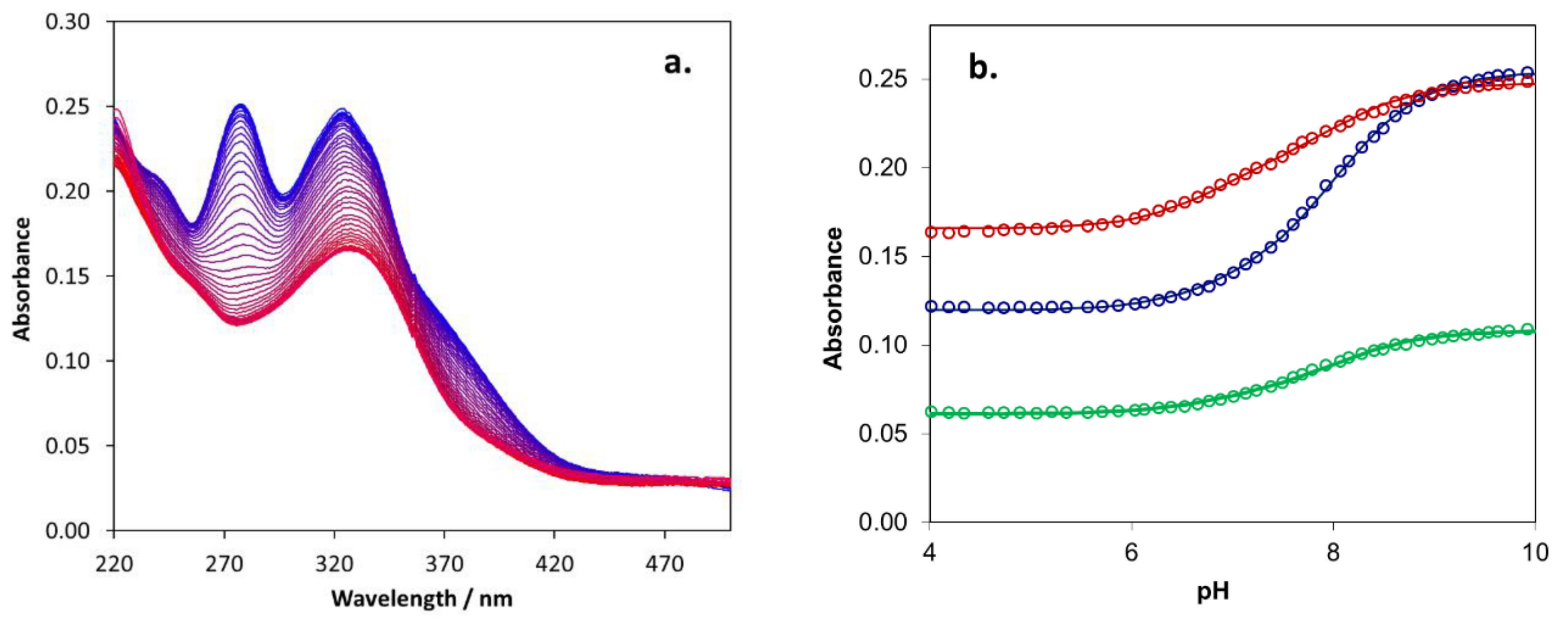
The measured absorption spectra (**a**) during potentiometric titration of ligand (*trans*-**6**). The pH changes from 10.0 (blue) to 4.0 (red). Subfigure (**b**) shows the measured (circles) and the calculated absorbance (lines) at three characteristic wavelengths (277 nm (blue), 324 nm (red), and 380 nm (green)).

Figure 12a well demonstrates a characteristic spectral change for the titration of *trans*-**6** with iron(III). The spectral series clearly indicates that the spectra cannot be the linear combinations of only two absorbing species (i.e., the organic ligand (HL) and the Fe^3+^ ion). The third one in this equilibrium system must be a complex between the previous two species. Detailed fitting studies by application of the PSEQUAD program confirmed the existence of a complex with a 1:1 (FeL^2+^) composition. Furthermore, the apparent formation constant of these species (for the reaction Fe^3+^ + HL ⇌ FeL^2+^ + H^+^) and the individual spectrum of the species could be estimated by this procedure. Figure 12b shows the spectrum of the complex compared to those of the ligand (HL) and Fe^3+^. It is clearly visible that the absorbance of Fe^3+^ cannot disturb the determination of the spectrum of the complex species, which significantly differs from that of the ligand. The apparent formation constant determined from this spectrum series and that regarding the titration with the ligand was lgK’_FeL_ = 4.75 ± 0.10. Using this apparent formation constant, the molar fractions of the free Fe^3+^ and the 1:1 complex could be calculated as functions of the (protonated) ligand concentration (pHL), as shown by the inset of Figure 12a. Since the spectrophotometric titration was carried out in an acidic solution, in order to estimate the formation constant for the Fe^3+^ + L^−^ ⇌ FeL^2+^ reaction, the protonation constant of the deprotonated ligand (L^−^) was also determined (by potentiometric titration, starting from the fully deprotonated form at pH = 10, Figure 13), lgK_1_ = 7.81 ± 0.15. The sum of the apparent formation constant and the protonation constant gives the equilibrium constant regarding the reaction above (lgK_FeL_ = 12.56 ± 0.15).

The protonation and complex formation constants were determined similarly for the other three compounds. The corresponding figures can be found in the Supplementary Materials (Figures S67 and S68 for *cis*-**2**, Figures S69 and S70 for *cis*-**4**, and Figures S71 and S72 for *trans***-1**). The protonation and complex formation constants obtained for the four compounds are summarized in Table 2. In the case of *trans*-**1,** two protonation steps were observed due to the two OH groups. For calculating the formation constant, lgK_2_ was taken into account, corresponding to the proton in the OH group on the pyranone ring. This can be exchanged by the Fe^3+^ ion easier than the other proton in the phenolic moiety.

The similar values of the formation constants (lgK = 11.6–12.6) suggest that Fe^3+^ binds to the common hydroxy-pyranone moiety, and the other different aromatic part of the ligands hardly influences the coordination of the metal center. In accordance with this conclusion, the characteristic bands in the individual absorption spectra of the complexes are uniformly red-shifted compared to those in the corresponding ligand spectra.

### 2.6. Crystal Structure and Packing of Resveratrol–Maltol Hybrids

**Figure 14:**
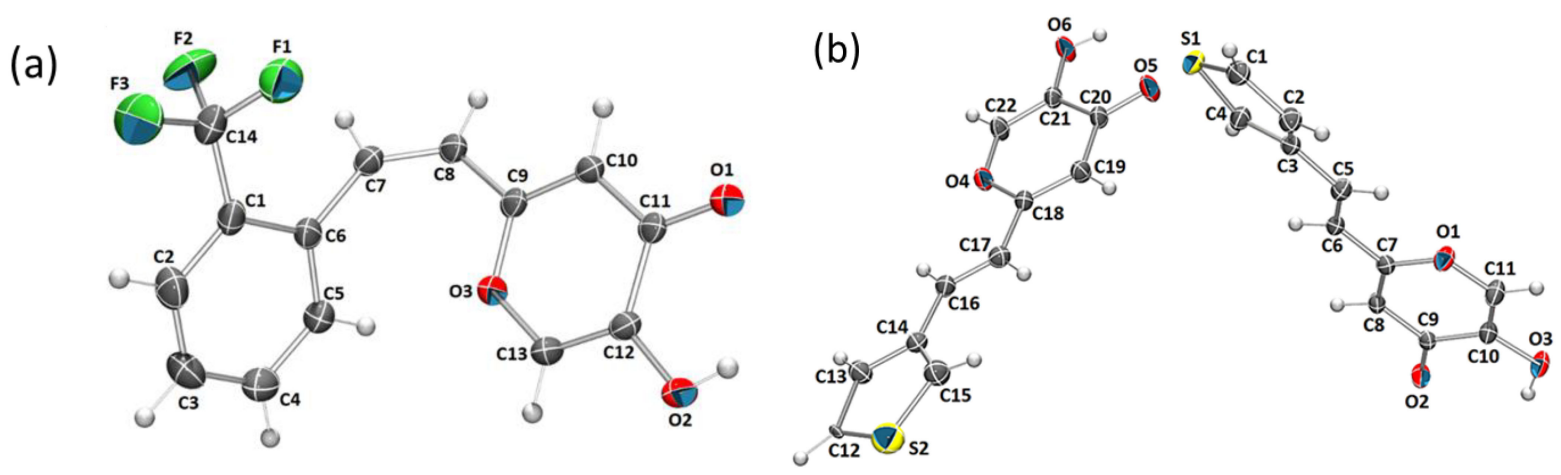
Molecular structure of compounds *cis*-**2** (**a**) and *trans*-**6** (**b**). Displacement ellipsoids are drawn for the probability of 25%, and hydrogen atoms are shown as spheres of arbitrary radii.

The molecular structures of two resveratrol–maltol hybrids, *cis*-**2** and *trans*-**6,** are shown in Figure 14. Although the crystallographic analyses of *cis*-**2** and *trans*-**6** are solid, they represent useful additional information about proven active compounds that can be informative in the context of intermolecular interaction study.

The crystal packing of compounds depends on a subtle interplay of weak interaction and steric effects. The molecules are held together by weak intermolecular interactions: hydrogen bonds, C–H⋯*π* interactions, and dispersion interactions.

**Figure 15:**
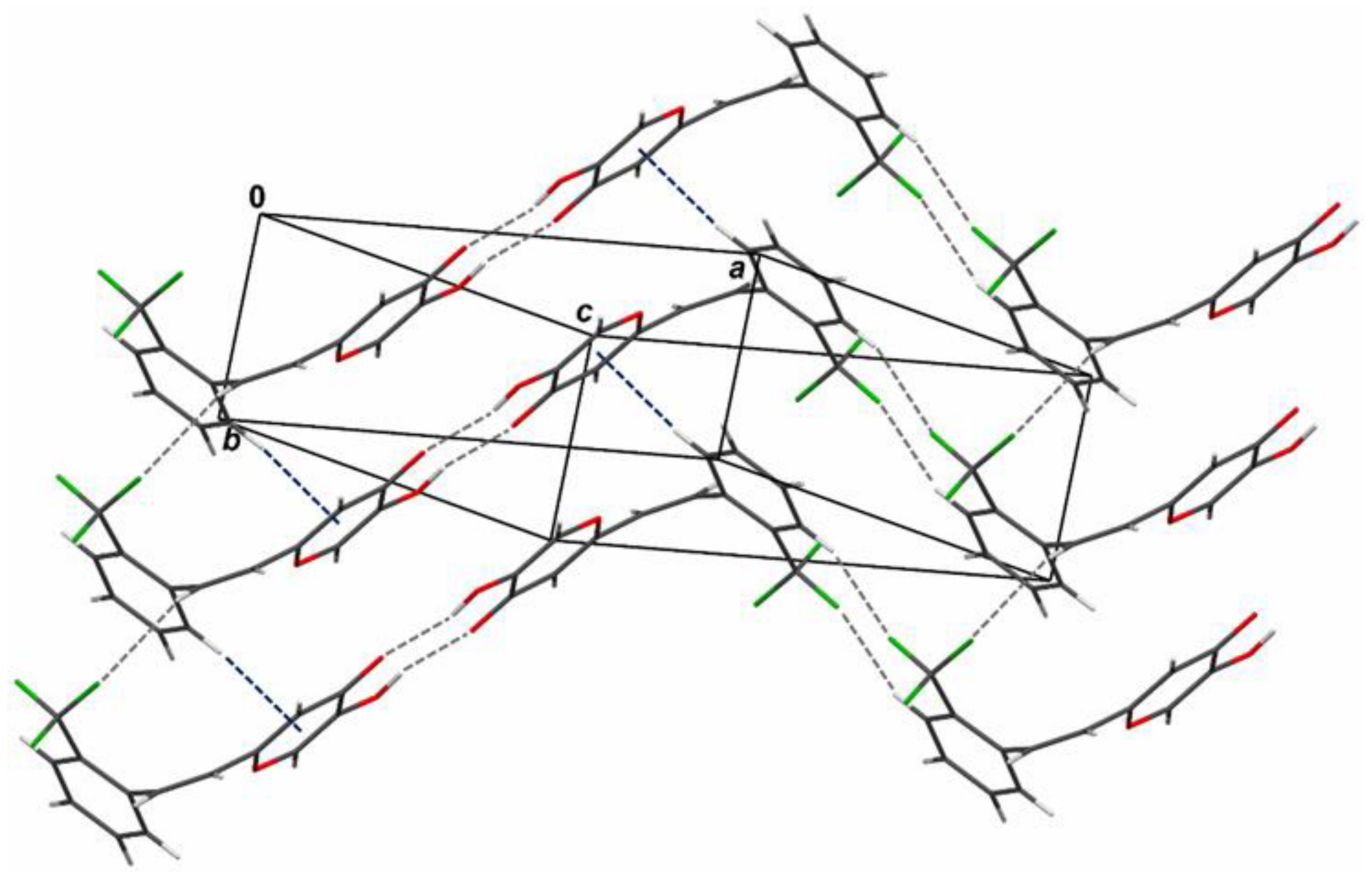
Crystal packing of *cis*-**2**. The chains are generated by C–H⋯F and O–H⋯O hydrogen bonds parallel to [1,2,3,4,5,6,7,8,9,10]. Hydrogen bond C7–H7⋯F1 and C–H⋯*π* interactions extend the chains in the direction [010].

In crystal packing of *cis*-**2** intermolecular hydrogen bonds (C2–H2⋯F3 and O2–H2A⋯O1) link two molecules into zig-zag chains parallel to [1,2,3,4,5,6,7,8,9,10] (Figure 15 and Table S5). The chains are linked by hydrogen bond C7–H7⋯F1 and C–H⋯*π* interactions (C5–H5⋯O3→C13) extending in the direction [010], which produces a 3D structure (Figure 15 and Table S6).

**Figure 16:**
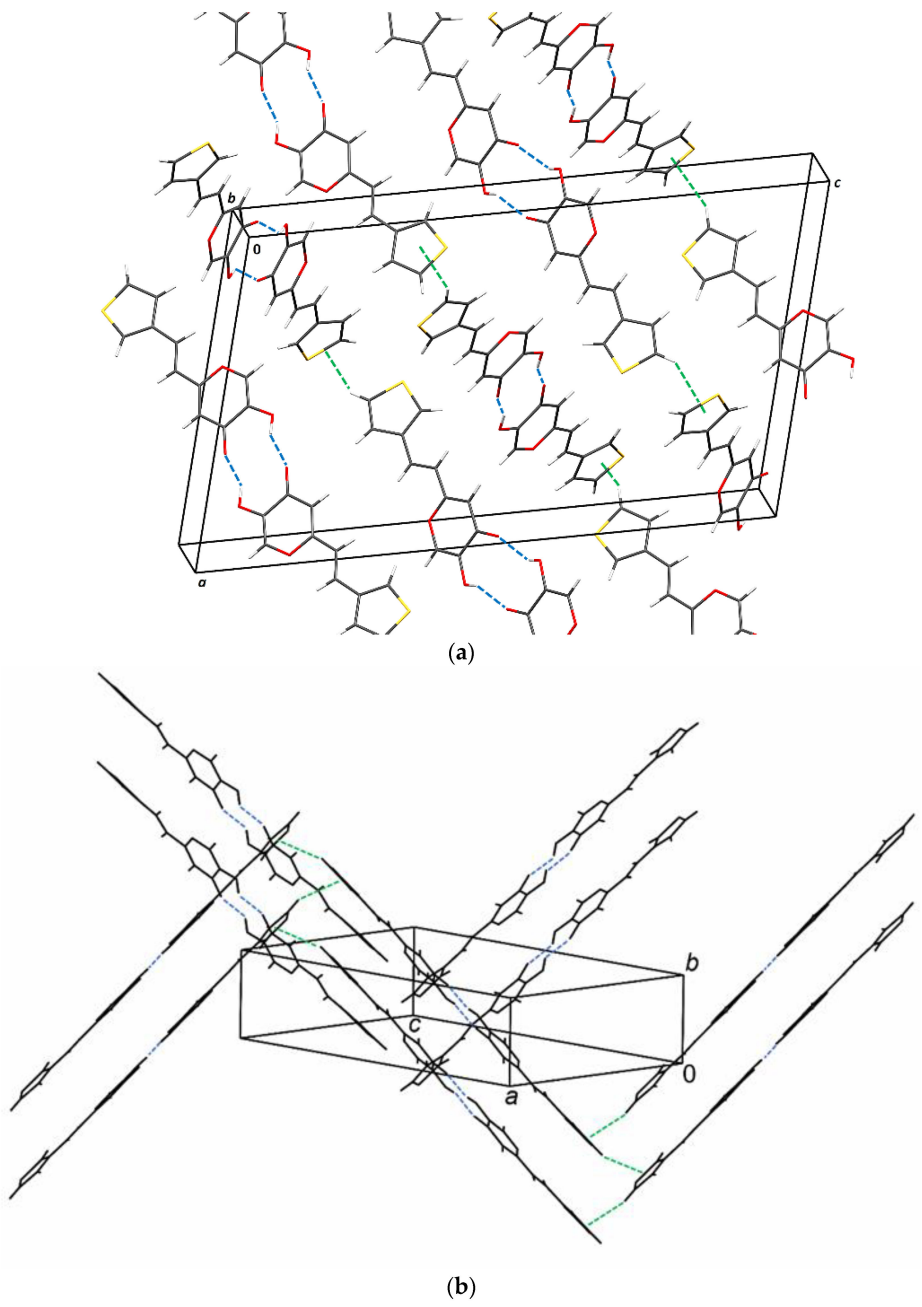
Crystal packing of *trans*-**6**: (**a**) layers generated by hydrogen bonds (O–H⋯O) and C–H⋯*π* interactions; (**b**) zig-zag chains in the direction [–1–10].

The crystal packing of *trans*-**6** is characterized by intermolecular hydrogen bonds (O–H⋯O) and C–H⋯*π* interactions (C5–H5⋯O3→C13), which generate zig-zag chains running in the direction [–1–10] (Figure 16 and Tables S5 and S6) 3D packing is achieved through stacking interactions (Table S7).

## 3. Materials and Methods

### 3.1. General Remarks

Solvents such as acetonitrile (ACN), chloroform (CHCl_3_), dichloromethane (DCM), diethylether (E), ethanol (EtOH), ethyl acetate (EtOAc), methanol (MeOH), and toluene were used. All enumerated solvents were purified by distillation and were commercially available. Iron(III) sulfate hexahydrate (Fe_2_(SO_4_)_3_·6H_2_O), sodium tetraborate decahydrate (Na_2_B_4_O_7_·10H_2_O), disodium hydrogen phosphate heptahydrate (Na_2_HPO_4_·7H_2_O), and sodium acetate trihydrate (CH_3_COONa·3H_2_O) were purchased from Reanal (Budapest, Hungary), and sodium hydroxide > 98% (NaOH) and 98% sulphuric acid from Acros Organics (Geel, Belgium). The water applied was cleaned by Millipure Elix equipment (Millipore S.A.S., Molsheim, France) and completed with a Milli-Q 50 purification system (Millipore S.A.S., Molsheim, France). The column chromatography was performed using silica gel (60 Å, technical grade). Thin-layer chromatography was performed using plates coated with silica gel (0.2 mm, Kiselgel 60 F_254_). Nuclear magnetic resonance (NMR) spectroscopic data for ^1^H and ^13^C nuclei were recorded at room temperature on a spectrometer at Bruker Avance 300 MHz and 600 MHz. Deuterated chloroform, CDCl_3_, deuterated methanol, CD_3_OD, and deuterated dimethyl sulfoxide, DMSO with tetramethylsilane as standard were used for recording NMR spectra. Chemical shifts were reported in parts per million. The ^1^H NMR spectra for four compounds were also recorded at 5, 15, 25, 35, 45, and 55 °C on a Bruker Advance 600 MHz spectrometer. UV spectra were recorded by UV/Vis spectrophotometer (Varian Cary 50 UV/Vis, Lexington, MA, USA) in ACN. Photochemical irradiations were carried out with 3.0 mL solutions in 1 mL cells at room temperature in photochemical reactor Luzchem equipped with 8 UV lamps at 313 nm. Spectral changes during the irradiation of pyranones were recorded in HPLC grade ACN. Before irradiation, the reaction mixtures were purged with nitrogen, N_2,_ for 15 min. IR spectra were acquired using the Bruker Vertex 70 Fourier transform infrared spectrometer set on attenuated total reflectance (ATR) mode. The samples were pressed on a diamond, and the absorbance data were collected between 400 and 5000 cm^−1^ with a spectral resolution of 1 cm^−1^ and an average of 32 scans. HRMS analyses were carried out on a mass spectrometer (MALDI TOF/TOF analyzer), equipped with an Nd:YAG laser operating at 355 nm with a fitting rate of 200 Hz in the positive (H+) or negative (-H) ion reflector mode. All solvents were removed from the solutions by rotary evaporator under reduced pressure.

### 3.2. General Procedure for the Synthesis of 4-Pyranone Phosphonium Salt

The 4-pyranone phosphonium salt was synthesized in a three-necked flask (0.25 L) by preparing corresponding pyranone chloride. Thionyl chloride, SOCl_2_ (0.09 mol) was added dropwise to a cooled (0 °C) reaction flask solution of kojic acid (0.05 mol) and DCM (82 mL). The reaction mixture was stirred for 5 h, and the rest of the solvent was evaporated. Crude yellow powder was dissolved in toluene, and triphenylphosphine (0.05 mol) was added and stirred for 3 days and 4 nights at reflux temperature. The reaction mixture was filtrated under reduced pressure, and the beige salt was dried under pressure in an exicator for 12 h. Dried phosphonium salt was used in all further experiments.

### 3.3. General Procedure for the Synthesis of 4-Pyranone Heterostilbenes ***1***–***8***

Compounds **1**–**8** were obtained as mixtures of *cis*- and *trans*-isomers (except 1 and 5). The reaction apparatus was purged with N_2_ for 15 min before adding the reactants. The reaction is carried out in a three-necked flask (100 mL) equipped with a chlorine-calcium tube and an N_2_ balloon connected. Phosphonium salt (5 mmol) was added to the 40 mL of EtOH, and the mixture was stirred with a magnetic stirrer. The solutions of sodium ethoxides (5 mmol, 1.1 eq of Na dissolved in 10 mL of absolute ethanol) were added in strictly anhydrous conditions under nitrogen dropwise. The aldehyde (5 mmol) was then added to the reaction mixture, and the reaction mixture was allowed to stir for 24 h at room temperature. The reaction mixture was evaporated on a vacuum evaporator and dissolved in toluene. The mixture was then extracted with toluene (3 × 15 mL). The organic layers were dried under anhydrous magnesium sulfate, MgSO_4_. Products 1–8 were isolated by repeated column and thin layer chromatography on silica gel using PE/E, PE/DCM, and DCM/E solvent systems. The first isomer to eluate is *cis*- and then *trans*-isomer in the last fractions. Spectroscopic characterization of new heterostilbene derivatives of 4-pyranone is given below. The solubility of pyranone derivatives was, in some cases low, especially for corresponding *trans*-isomers.

5-hydroxy-2-(2-hydroxystyryl)-4*H*-pyran-4-one (1) (63%). Column chromatography on silica gel using EtOAc/MeOH (50%) afforded 500 mg of a mixture of *trans*-isomer and triphenylphosphine oxide. Repeated column chromatography on silica gel using EtOAc/MeOH (50%) afforded pure *trans*-isomer.


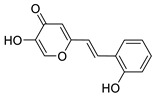


(*E*)-5-hydroxy-2-(2-hydroxystyryl)-4*H*-pyran-4-one (*trans*-1): 88 mg (isolated), orange powder, *R_f_*(E) = 0.12; m.p. 185–186 °C; UV (ACN with small amount of MeOH) *λ_max_*/nm (*ε*/dm^3^mol^−1^cm^−1^) 353 (29371); IR *v*_max_/cm^−1^ (NaCl): 3411, 3248, 1630, 1552, 1421, 1321, 1248, 1040, 963, 939; ^1^H NMR (CD_3_OD, 600 MHz) *δ*/ppm: 7.83 (s, 1H), 7.70 (d, *J* = 16.6 Hz, 1H), 7.51 (d, *J* = 7.4 Hz, 1H), 7.17 (t, *J* = 8.0 Hz, 1H), 7.00 (d, *J* = 16.3 Hz, 1H), 6.84 (t, *J* = 7.5 Hz, 3H); ^13^C NMR (DMSO, 75 MHz) *δ*/ppm: 144.2 (s), 136.4 (s), 131.9 (s), 130.9 (s), 128.5 (d), 124.5 (d), 124.2 (d), 119.6 (d), 116.3 (d), 116.2 (d), 105.1 (s), 107.6 (d), 99.4 (d); MS (ESI) *m/z* (%, fragment): 231 (100); HRMS (*m/z*) for C_13_H_10_O_4_: [M+H]^+^_calcd_ = 230.0579, [M+H]^+^_measured_ = 230.0583.

5-hydroxy-2-(2-(trifluoromethyl)styryl)-4*H*-pyran-4-one (2) (59%). Column chromatography on silica gel using PE/E (0–100%) afforded a mixture of isomers of 2 (*cis*-2: *trans*-2 = 1:1.5). Repeated thin-layer chromatography on silica gel using PE/E (30%) as eluent afforded pure *cis*-2 in the first fractions and *trans*-2 in the last fractions.


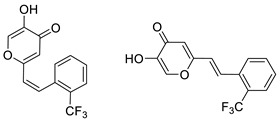


(*Z*)-5-hydroxy-2-(2-(trifluoromethyl)styryl)-4*H*-pyran-4-one (*cis*-2): 14.2 mg (isolated), white powder, *R_f_*(E) = 0.45; m.p. 113–115 °C; UV (ACN) *λ_max_*/nm (*ε*/dm^3^mol^−1^cm^−1^) 280 (11679); IR *v*_max_/cm^−1^ (NaCl): 3230, 2920, 2850, 1650, 1580, 1450, 1380, 1250, 930, 860, 770, 690, 580; ^1^H NMR (CDCl_3_, 600 MHz) *δ*/ppm: 7.72 (d, *J* = 8.4 Hz, 1H), 7.50 (t, *J* = 7.1 Hz, 1H), 7.50 (s, 1H), 7.45 (t, *J* = 7.6 Hz, 2H), 7.14 (d, *J* = 12.3 Hz, 1H), 6.35 (d, *J* = 12.3 Hz, 1H), 6.21 (s, 1H); ^13^C NMR (CDCl_3_, 150 MHz) *δ*/ppm: 173.7 (s), 161.8 (s), 145.7 (d), 137.0 (d), 135.1 (d), 134.7 (s), 131.6 (d), 130.3 (d), 128.6 (d), 126.0 (s), 125.9 (s), 123.5 (d), 112.5 (d); MS (ESI) *m/z* (%, fragment): 283 (100);

(*E*)-5-hydroxy-2-(2-(trifluoromethyl)styryl)-4*H*-pyran-4-one (*trans*-2): 21.6 mg (isolated), white powder, *R_f_*(E) = 0.45; m.p. 117–120 °C; UV (ACN) *λ_max_*/nm (*ε*/dm^3^mol^−1^cm^−1^) 308 (8706); IR *v*_max_/cm^−1^ (NaCl): 3230, 2920, 2850, 1650, 1580, 1450, 1380, 1250, 930, 860, 770, 690, 580; ^1^H NMR (CDCl_3_, 600 MHz) *δ*/ppm: 7.90 (s, 1H), 7.78 (d, *J* = 15.9 Hz, 1H), 7.72 (d, *J* = 7.2 Hz, 2H), 7.61 (t, *J* = 7.2 Hz, 1H), 7.51 (t, *J* = 7.6 Hz, 1H), 6.71 (d, *J* = 15.9 Hz, 1H), 6.49 (s, 1H); ^13^C NMR (CDCl_3_, 150 MHz) *δ*/ppm: 174.2 (s), 161.8 (s), 145.6 (s), 136.9 (d), 132.3 (d), 131.9 (d), 129.1 (d), 127.3 (d), 126.3 (s), 126.2 (s), 123.4 (d), 111.3 (d); MS (ESI) *m/z* (%, fragment): 283 (100);

HRMS (*m/z*) for C_14_H_9_F_3_O_3_ (obtained for the pure mixture of geometrical isomers): [M+H]^+^_calcd_ = 282.0504, [M+H]^+^_measured_ = 282.0505.

4-(2-(5-hydroxy-4-oxo-4*H*-pyran-2-yl)vinyl)benzonitrile (3) (44%). Column chromatography on silica gel using PE/E (80%) afforded a pure mixture of isomers of 3 (41 mg; *cis*-3:*trans*-3 = 1:3) without triphenylphosphine-oxide. Repeated column chromatography on silica gel using PE/E (0–70%) as eluent did not afford pure *cis*- and *trans*-isomers due to the very similar R*_f_* values.


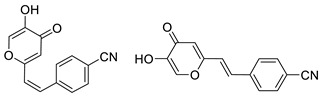


(*Z*)-4-(2-(5-hydroxy-4-oxo-4*H*-pyran-2-yl)vinyl)benzonitrile (*cis*-3): ^1^H NMR (DMSO-d6, 600 MHz) *δ*/ppm: 8.93 (s, 1H), 7.74 (d, *J* = 8.2 Hz, 2H), 7.4 (d, *J* = 8.1 Hz, 2H), 7.10 (d, *J* = 12.5 Hz, 1H), 6.84 (d, *J* = 12.1 Hz, 1H), 6.79 (s, 1H); MS (ESI) *m/z* (%, fragment): 240 (100).

(*E*)-4-(2-(5-hydroxy-4-oxo-4*H*-pyran-2-yl)vinyl)benzonitrile (*trans*-3): ^1^H NMR (DMSO-d6, 600 MHz) *δ*/ppm: 9.27 (s, 1H), 7.86 (d, *J* = 8.5 Hz, 2H), 7.76 (d, *J* = 8.5 Hz, 2H), 7.68 (d, *J* = 16.2 Hz, 1H), 7.15 (d, *J* = 16.2 Hz, 1H), 6.67 (s, 1H); MS (ESI) *m/z* (%, fragment): 240 (100);

IR *v*_max_/cm^−1^ (NaCl) (obtained for the pure mixture of geometrical isomers): 3050, 2910, 2840, 2250, 1715, 1570, 1480, 950, 840, 550; HRMS (*m/z*) for C_14_H_9_NO_3_ (obtained for the pure mixture of geometrical isomers): [M+H]^+^_calcd_ = 239.0582, [M+H]^+^_measured_ = 239.0588.

2-(2,4-dichlorostyryl)-5-hydroxy-4*H*-pyran-4-one (4) (74%). Column chromatography was performed on silica gel using PE/E (0–70%) as eluent. Repeated column chromatography on silica gel (PE/E (0–70%)) afforded pure *cis*-4 in the first fractions and *trans*-4 in the last fractions.


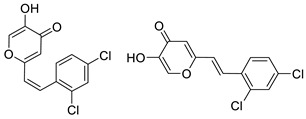


(*Z*)-2-(2,4-dichlorostyryl)-5-hydroxy-4*H*-pyran-4-one (*cis*-4): 250 mg (isolated), white powder, *R_f_*(E) = 0.51; m.p. 149–151 °C; UV (ACN) *λ_max_*/nm (*ε*/dm^3^mol^−1^cm^−1^) 291 (14966); IR *v*_max_/cm^−1^ (NaCl): 3200, 3050, 1600, 1550, 1490, 1380, 1250, 1150, 950, 750, 700; ^1^H NMR (CDCl_3_, 600 MHz) *δ*/ppm: 7.58 (s, 1H), 7.45 (d, *J* = 2.0 Hz, 1H), 7.21 (d, *J* = 2.0 Hz, 1H), 6.90 (d, *J* = 12.3 Hz, 1H), 6.36–6.31 (m, 2H); ^13^C NMR (CDCl_3_, 75 MHz) *δ*/ppm: 173.7 (s), 161.8 (s), 145.7 (s), 136.7 (d), 135.1 (s), 134.1 (s), 133.9 (d), 132.8 (s), 130.8 (d), 129.4 (d), 126.9 (d), 123.1 (d), 112.5 (d); MS (ESI) *m/z* (%, fragment): 283 (100).

(*E*)-2-(2,4-dichlorostyryl)-5-hydroxy-4*H*-pyran-4-one (*trans*-4): 40 mg (isolated), white powder, *R_f_*(E) = 0.51; m.p. 103–105 °C; UV (ACN) *λ_max_*/nm (*ε*/dm^3^mol^−1^cm^−1^) 325 (23654), 288 (20467); ^1^H NMR (CDCl_3_, 600 MHz) *δ*/ppm: 8.06 (s, 1H), 7.93 (s, 1H), 7.74 (d, *J* = 16.2 Hz, 1H), 7.58 (d, *J* = 7.7 Hz, 1H), 7.57 (d, *J* = 8.4 Hz, 1H), 6.68 (d, *J* =16.2 Hz, 1H), 6.56 (s, 1H); ^13^C NMR (CDCl_3_, 75 MHz) *δ*/ppm: 174.1 (s), 161.8 (s), 145.7 (s), 137.5 (s), 135.2 (s), 131.1 (d), 130.2 (d), 130.0 (d), 128.4 (d), 127.7 (d), 127.6 (d), 122.1 (s), 111.3 (d); MS (ESI) *m/z* (%, fragment): 283 (100);

HRMS (*m/z*) for C_13_H_8_Cl_2_O_3_ (obtained for the pure mixture of geometrical isomers): [M+H]^+^_calcd_ = 281.9850, [M+H]^+^_measured_ = 281.9852.

*N*-(4-(2-(5-hydroxy-4-oxo-4*H*-pyran-2-yl)vinyl)phenyl)acetamide (5) (53%). Repeated column chromatography was performed on silica gel with solvent system EtOAc/MeOH (20%). This derivative gave only *cis*-isomer as a product of the Wittig reaction, but repeated chromatography was necessary to divide the triphenylphosphine-oxide from *cis*-5.


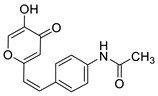


(*Z*)-*N*-(4-(2-(5-hydroxy-4-oxo-4*H*-pyran-2-yl)vinyl)phenyl)acetamide (*cis*-5): 100 mg (isolated), orange powder, *R_f_*(DCM:MeOH = 7:3) = 0.35; m.p. 154–156 °C; UV (ACN) *λ_max_*/nm (*ε*/dm^3^mol^−1^cm^−1^) 338 (10321); IR *v*_max_/cm^−1^ (NaCl): 3372, 1639, 1582, 1534, 1321, 1210, 1178, 1040, 970, 930, 825; ^1^H NMR (CD_3_OD, 600 MHz) *δ*/ppm: 6.28 (s, 1H), 6.04 (d, *J* = 8.2 Hz, 3H), 5.76 (d, *J* = 8.0 Hz, 2H), 5.40 (d, *J* = 12.3 Hz, 1H), 4.73 (d, *J* = 12.3 Hz, 1H), 3.34 (s, 3H); ^13^C NMR (CD_3_OD, 75 MHz) *δ*/ppm: 174.4 (s), 166.9 (s), 141.7 (s), 139.8 (s), 134.9 (s), 133.2 (d), 132.0 (d), 123.6 (d), 123.2 (d), 121.7 (d), 116.6 (d), 114.6 (s), 26.5 (q); MS (ESI) *m/z* (%, fragment): 272 (100);

HRMS (*m/z*) for C_15_H_13_NO_4_: [M+H]^+^_calcd_ = 271.0845, [M+H]^+^_measured_ = 271.0850.

5-hydroxy-2-(2-(thiophen-3-yl)vinyl)-4*H*-pyran-4-one (6) (38%). Repeated column chromatography was performed on silica gel using PE/DCM (0–90%), DCM/E (30%), and DCM/MeOH (10%) as solvent systems in combination with preparative thin-layer chromatography in a solvent system PE/E (30%). Pure *cis*-6 was isolated in the first fractions and *trans*-6 in the last fractions.


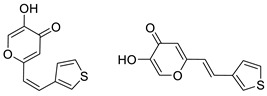


(*Z*)-5-hydroxy-2-(2-(thiophen-3-yl)vinyl)-4*H*-pyran-4-one (*cis*-6): 7 mg (isolated), white powder, *R_f_*(E) = 0.55; m.p. 143–146 °C; UV (ACN) *λ_max_*/nm (*ε*/dm^3^mol^−1^cm^−1^) 311 (11679); IR *v*_max_/cm^−1^ (NaCl): 3220, 2925, 2840, 1740, 1650, 1580, 1470, 1435, 1380, 950, 825, 770, 725; ^1^H NMR (CDCl_3_, 600 MHz) *δ*/ppm: 7.77 (s, 1H), 7.39 (dd, *J* = 2.9 Hz, 1H), 7.29 (dd, *J* = 3.0 Hz, 1H), 7.09 (dd, *J* = 5.0 Hz, 1H), 6.81 (d, *J* = 12.6 Hz, 1H), 6.47 (s, 1H), 6.10 (d, *J* = 12.5 Hz, 1H); ^13^C NMR (CDCl_3_, 150 MHz) *δ*/ppm: 173.9 (s), 162.9 (s), 145.9 (s), 136.7 (d), 136.3 (s), 132.8 (d), 127.8 (d), 127.4 (d), 125.9 (d), 119.2 (d), 111.9 (d); MS (ESI) *m/z* (%, fragment): 221 (100).

(*E*)-5-hydroxy-2-(2-(thiophen-3-yl)vinyl)-4*H*-pyran-4-one (*trans*-6): 9.1 mg (isolated), yellow powder, *R_f_*(E) = 0.55; m.p. 111–115 °C; UV (ACN) *λ_max_*/nm (*ε*/dm^3^mol^−1^cm^−1^) 314 (20340); IR *v*_max_/cm^−1^ (NaCl): 3220, 2925, 2840, 1740, 1650, 1580, 1470, 1435, 1380, 950, 825, 770, 725; ^1^H NMR (CDCl_3_, 600 MHz) *δ*/ppm: 7.83 (s, 1H), 7.48 (d, *J* = 16.3 Hz, 1H), 7.37 (dd, *J* = 5.2 Hz, 1H), 7.32 (dd, *J* = 5.2 Hz, 1H), 7.29 (dd, *J* = 5.4 Hz, 1H), 6.53 (d, *J* = 16.3 Hz, 1H), 6.48 (s, 1H); ^13^C NMR (CDCl_3_, 150 MHz) *δ*/ppm: 174.2 (s), 162.8 (s), 145.5 (s), 138.0 (s), 136.8 (d), 130.3 (d), 127.2 (d), 126.9 (d), 124.8 (d), 119.1 (d), 110.1 (d); MS (ESI) *m/z* (%, fragment): 221 (100);

HRMS (*m/z*) for C_11_H_8_O_3_S (obtained for the pure mixture of geometrical isomers): [M+H]^+^_calcd_ = 220.0194, [M+H]^+^_measured_ = 220.0199.

5-hydroxy-2-(2-(thiophen-2-yl) vinyl)-4*H*-pyran-4-one (7) (42%). Repeated column chromatography was performed on silica gel with solvent system PE/E (0–50%). After that, repeated thin-layer chromatography of some enriched fractions was performed with a solvent system E/CHCl_3_ (80%). Only the isolation of the pure *trans*-7 in the last fractions was successful, while the obtained pure mixture of isomers (13 mg) without triphenylphosphine-oxide confirmed the formation of *cis*-7 (*cis*-7:*trans*-7 = 1:1).


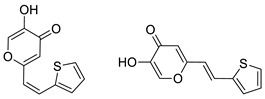


(*Z*)-5-hydroxy-2-(2-(thiophen-2-yl)vinyl)-4*H*-pyran-4-one (*cis*-7): ^1^H NMR (CDCl_3_, 600 MHz) *δ*/ppm: 7.91 (s, 1H), 7.44 (d, *J* = 5.2 Hz, 1H), 7.25 (d, *J* = 3.5 Hz, 1H), 7.06 (dd, *J* = 5.2 Hz, 1H), 6.90 (d, *J* = 12.8 Hz, 1H), 6.47 (s, 1H), 6.0 (d, *J* = 12.8 Hz, 1H); MS (ESI) *m/z* (%, fragment): 221 (100).

(*E*)-5-hydroxy-2-(2-(thiophen-2-yl)vinyl)-4*H*-pyran-4-one (*trans*-7): 8 mg (isolated), white powder, *R_f_*(E) = 0.35; m.p. 113–116 °C; UV (ACN) *λ_max_*/nm (*ε*/dm^3^mol^−1^cm^−1^) 340 (30518); IR *v*_max_/cm^−1^ (NaCl): 3210, 2920, 2840, 1730, 1660, 1580, 1470, 1435, 1390, 1210, 950, 850, 760, 680; ^1^H NMR (CDCl_3_, 600 MHz) *δ*/ppm: 7.83 (s, 1H), 7.52 (d, *J* = 16.5 Hz, 1H), 7.36 (d, *J* = 5.7 Hz, 1H), 7.22 (d, *J* = 3.5 Hz, 1H), 7.05 (dd, *J* = 5.0 Hz, 1H), 6.50 (d, *J* = 16.5 Hz, 1H), 6.39 (s, 1H); ^13^C NMR (CDCl_3_, 150 MHz) *δ*/ppm: 174.2 (s), 162.8 (s), 145.5 (s), 140.1 (s), 136.8 (d), 129.9 (d), 129.3 (d), 128.2 (d), 127.2 (d), 118.3 (d), 110.1 (d); MS (ESI) *m/z* (%, fragment): 221 (100);

HRMS (*m/z*) for C_11_H_8_O_3_S (obtained for the pure mixture of geometrical isomers): [M+H]^+^_calcd_ = 220.0194, [M+H]^+^_measured_ = 220.0200.

5-hydroxy-2-(2-(quinoxalin-2-yl)vinyl)-4*H*-pyran-4-one (8) (35%). Repeated column and thin-layer chromatographies (E/EtOAc (0–70%) and EtOAc/MeOH (0–90%)) were performed on silica gel. The pure mixture of isomers was obtained (12 mg) without the presence of triphenylphosphine-oxide (*cis*-8:*trans*-8 = 1.3:1).


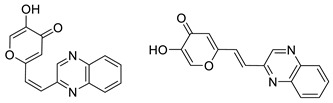


(*Z*)-5-hydroxy-2-(2-(quinoxalin-2-yl)vinyl)-4*H*-pyran-4-one (*cis*-8): ^1^H NMR (DMSO-d6, 600 MHz) *δ*/ppm: 8.93 (s, 1H), 8.13–8.08 (m, 2H), 7.92–7.86 (m, 3H), 6.84 (d, *J* = 12.7 Hz, 1H), 6.79 (d, *J* = 12.7 Hz, 1H), 6.79 (s, 1H); MS (ESI) *m/z* (%, fragment): 267 (100).

(*E*)-5-hydroxy-2-(2-(quinoxalin-2-yl)vinyl)-4*H*-pyran-4-one (*trans*-8): ^1^H NMR (DMSO-d6, 600 MHz) *δ*/ppm: 9.27 (s, 1H), 8.13–8.08 (m, 2H), 8.02 (d, *J* = 9.5 Hz, 1H), 7.92–7.86 (m, 2H), 7.75 (d, *J* = 16.0 Hz, 1H), 7.68 (d, *J* = 16.0 Hz, 1H), 6.67 (s, 1H); MS (ESI) *m/z* (%, fragment): 267 (100);

IR *v*_max_/cm^−1^ (NaCl) (obtained for the pure mixture of geometrical isomers): 3320, 2959, 2917, 2847, 1584, 1568, 1377, 1261, 1088, 1014, 794, 749; HRMS (*m/z*) for C_15_H_10_N_2_O_3_ (obtained for the pure mixture of geometrical isomers): [M+H]^+^_calcd_ = 266.0691, [M+H]^+^_measured_ = 266.0697.

### 3.4. Cholinesterase Inhibitory Activity

Acetylcholinesterase (AChE, E.C. from electric eel), butyrylcholinesterase (BChE, E.C. from equine serum), Tris-HCl buffer, acetylthiocholine iodide (ATChI), *S*-butyrylthiocholine iodide (BTChI) and galantamine were purchased from Sigma-Aldrich (St. Louis, MO, USA), 5,50-dithiobis-(2-nitrobenzoic acid) (Ellman’s reagent, DTNB) was purchased from Zwijndrecht (Belgium). The AChE/BChE inhibitory potentials were determined using modified Ellman’s method [38]. Galantamine was used as a reference standard. Briefly, 180 µL Tris HCl buffer (50 mM, pH 8.0), 10 µL of AChE/BChE (final concentration 0.03 U/mL, prepared in 20 mM Tris HCl buffer, pH 7.5), and 10 µL tested solution (final concentrations, depending on solubility and activity, in a range 1–1000 μM) were mixed and pre-incubated for 5 min at 4 °C. The reaction was then initiated with the addition of 10 μL of DTNB (final concentration 0.3 mM prepared in Tris buffer) and 10 μL of ATChI/BTChI (final concentration of 0.5 mM prepared in Tris buffer). AChE/BChE activity was measured using a 96-well microplate reader (IRE 96, SFRI Medical Diagnostics, Bordeaux, France) at room temperature at 405 nm over 6 min. Non-enzymatic hydrolysis was measured as blank for control measurement without inhibitors. The non-enzymatic hydrolysis reaction with added inhibitor was used as a blank for the samples. An equivalent buffer amount replaced the enzyme. The experiment was run in triplicate. Percentage enzyme inhibition was calculated according to the equation:

*Inhibition* (%) = [(*A*_C_ − *A*_T_)/*A*_C_] × 100 (1)

where *A*_C_ is the enzyme activity without the test sample, and A_T_ is the enzyme activity with the test sample. Data were used to calculate the IC_50_ value by a nonlinear fit of compound concentration (log) values vs. response. The results are represented as mean values ± standard deviation. Samples were dissolved in ethanol. The inhibitory activity of ethanol was measured, and its contribution to inhibition was subtracted.

Antioxidant activity of the tested compounds was measured in terms of hydrogen donating or radical scavenging ability using the stable radical 2,2′-diphenyl-1-picrylhydrazyl (DPPH) [39]. The 50 μL of various concentrations of tested solutions (5 μM–1000 μM) were placed in a cuvette, 1 mL of ethanolic solution of DPPH was added, and the final DPPH concentration was kept constant (*c* = 8 × 10^−4^ M). The mixture was incubated for 30 min at 25 °C. The absorbance was measured at 517 nm (UV-1800 UV/Vis Spectrophotometer, Shimadzu, Kyoto, Japan). All determinations were performed in triplicate. The concentrations of tested compounds were expressed as final concentrations. Inhibition of DPPH expressed in percentage was calculated according to the equation:

(2) $$Inhibition\;\left(\%\right)=\frac{A_{C\left(0\right)}-A_{C\left(t\right)}}{A_{C\left(0\right)}}\cdot 100$$

where *A*_C(0)_ is the absorbance of the control at *t* = 0 min, and *A*_C(t)_ is the absorbance of the antioxidant at *t* = 30 min. Data were used to calculate the IC_50_ value by a nonlinear fit of compound concentration values vs. inhibition percentage. The results are represented as mean values ± standard deviation.

### 3.5. Molecular Docking

The conformations of selected ligands were examined at the M06-2X/6-31G(d) level of theory using the Gaussian16 program suite [43]. The optimized structures of the most stable conformers were then used for a molecular docking study. Molecular docking was performed using the Autodock program package [44], with crystal structures of cholinesterases taken from the Protein Data Bank: for AChE, crystal structure 1EEA.pdb [45], and for BChE, crystal structure 1P0I.pdb [46] was used. The docking results were obtained using the Lamarckian Genetic Algorithm, with 25 requested genetic algorithm dockings with 25 binding poses for each ligand.

### 3.6. Metal Chelating Affinity

Two titration series were used to determine the stability constants of equilibrium systems containing Fe^3+^ and ligand. The iron(III) content was increased at constant ligand content, the ligand concentration was increased at constant Fe(III) content, and the spectral changes were recorded with a Specord S600 spectrophotometer (Analytic Jena GmbH, Jena, Germany) in the 190–900 nm wavelength range. Since the molar absorption coefficient of the ligand is significantly higher than that of Fe(III), the former series provides more information for the determination of the spectra of the new complex formed, while the latter series is necessary for a more accurate determination of the stability constant. The two series were evaluated together using PSEQUAD software [47] in the 210–430 nm range.

A 0.01–0.01 M phosphate–borate–acetate buffer solution was used for potentiometric titration. A 50 cm^3^ beaker was filled with 25 cm^3^ of buffer solution containing the appropriate amount of ligand, and the pH was adjusted to 10.0 by adding 2 M NaOH solution. The pH was determined using a Consort C3010 multi-parameter analyzer (Consort bvba, Turnhout, Belgium) equipped with a combined glass electrode. The solution was circulated with an IKA PA-SF (IKA Labortechnik, Staufen, Germany) peristaltic pump through a Hellma (176.000-QS 10 mm) flow through the cuvette. Titration was carried out with 6 M H_2_SO_4_ solution. The spectra were recorded with a Specord S600 spectrophotometer after pH stabilization.

### 3.7. X-Ray Crystallography

Single crystal measurements were performed on an XtaLAB Synergy diffractometer, using micro-focus sealed X-ray tube Cu*K_α_* (1.54184 Å) radiation at room temperature [293(2) K]. The CrysAlisPRO package (Rigaku OD, 2018) was used for data reduction and numerical absorption correction. The structure was solved with SHELXS97 [48] and refined with SHELXL2018 [49]. The model was refined using the full matrix least squares refinement; all non-hydrogen atoms were refined anisotropically. Hydrogen atoms were located in a difference Fourier map and refined as a mixture of free and riding entities. Molecular geometry calculations were performed by PLATON [50], and the molecular graphics were prepared using ORTEP-3 [51] and CCDC-Mercury [52]. Crystallographic and structure refinement data for the structure reported in this paper are shown in Table 3. Supplementary crystallographic data for this paper can be obtained free of charge via www.ccdc.cam.ac.uk/conts/retrieving.html accessed on 2 September 2022 (or from the Cambridge Crystallographic Data Centre, 12, Union Road, Cambridge CB2 1EZ, UK; fax: +44 1223 336033; or deposit@ccdc.cam.ac.uk). CCDC-2204641 and CCDC-2204642 contain supplementary crystallographic data for this paper.

## 4. Conclusions

New resveratrol–thiophene and resveratrol–maltol hybrids were synthesized as cholinesterase inhibitors, metal-chelating agents, and antioxidants. As photostability is the favorable property of resveratrol derivatives, the aim of this study was also to investigate changes in the UV spectra of compounds when exposed to UV light, as well as the capability to complex with biometals. As with photostability experiments, biological tests also found remarkable differences in the properties and behavior of thiophene and maltol hybrids. While resveratrol–thiophene hybrids have excellent inhibitory and antioxidant properties (similar to the activity of the reference drug galantamine), maltols have been proven to be weaker inhibitors and antioxidants. The results of the docking study indicate that all docked ligands are accommodated by both enzymes, AChE and BChE, mainly through π–π stacking interactions. The compounds proved to be active cholinesterase inhibitors, also prone to coordination with the Fe^3+^ ion as biometals. In the crystal packing, they are held together by weak intermolecular interactions: hydrogen bonds, C–H⋯*π* interactions, and dispersion interactions.

## Figures and Tables

**Table 1:** ChE inhibitory effects and antioxidant activities for resveratrol–thiophene and the active resveratrol–maltol hybrids.

Resveratrol-Thiophene/ Resveratrol–Maltol Hybrids	AChE IC_50_ (μM)	BChE IC_50_ (μM)	DPPH IC_50_ (μM)
II	15.7 ± 1.0	4.6 ± 0.1	158.8 *
*cis*-II	~500	18.9 ± 3.4	782.1 *
III	46.6 ± 8.3	5.3 ± 1.8	26.8 *
*trans*-1	-	600.7 ± 34.5	251.3 ± 45.6
*cis*-2	-	458.1 ± 30.2	-
*cis*-4	1003.0 ± 10.1	638.1 ± 25.8	-
*trans*-6	-	426.3 ± 41.8	-
Galantamine	0.15	7.9	

* [26].

**Table 2:** Protonation constants for the deprotonated compounds (L^−^ or L^2−^) and the constants for the formation of FeL^2+^ or FeHL^2+^ complexes.

Compounds	Protonation Constants		Formation Constants (lgK_FeL_ or lgK_FeHL_^a, b^)	
(HL or H_2_L)	lgK_1_	lgK_2_	Fe^3+^ + HL ⇌ FeL^2+^ + H^+^	Fe^3+^ + L^−^ ⇌ FeL^2+^
*trans*-1	8.9 ± 0.20	7.25 ± 0.20	4.38 ± 0.20 ^a^	11.6 ± 0.2 ^b^
*cis*-2	7.95 ± 0.10		4.38 ± 0.20	11.8 ± 0.2
*cis*-4	7.99 ± 0.15		4.16 ± 0.10	12.15 ± 0.15
*trans*-6	7.81 ± 0.15		4.75 ± 0.10	12.56 ± 0.15

^a^ lgK_FeHL_ for Fe^3+^ + H_2_L ⇌ FeHL^2+^ + H^+^, ^b^ for Fe^3+^ + HL^−^ ⇌ FeHL^2+^.

**Table 3:** Crystallographic data and structure refinement details for *cis*-**2** and *trans*-**6**.

Compound	*cis*-2	*trans*-6
Empirical formula	C_14_H_9_F_3_O_3_	C_11_H_8_O_3_S
Formula wt./g mol^−1^	282.21	220.23
Crystal dimensions/mm	0.4 × 0.25 × 0.2	0.4 × 0.3 × 0.25
Space group	*P*2_1_/*c*	*P*2_1_/*c*
*a*/Å	18.0019 (10)	15.3582 (2)
*b*/Å	4.8342 (3)	5.14770 (10)
*c*/Å	14.7412 (8)	25.9362 (5)
*α*/°	90	90
*β*/°	107.425 (6)	102.554 (2)
*γ*/°	90	90
Z	4	8
*V*/Å^3^	1223.98 (13)	2001.48 (6)
*D*_calc_/g cm^−3^	1.531	1.462
*μ/*mm^−1^	1.2	2.748
*Θ* range/°	5.15–79.384	3.492–79.837
*T*(K)	293 (2)	299 (2)
Radiation wavelength	1.54184 (Cu*K_α_*)	1.54184 (Cu*K_α_*)
Diffractometer type	XtaLAB Synergy, Dualflex, HyPix	XtaLAB Synergy, Dualflex, HyPix
Range of *h*, *k*, *l*	−22 > *h* > 19; −5 > *k* > 5; −18 > *l* > 18	−19 > *h* > 16; −6 > *k* > 5; −31 > *l* > 33
Reflections collected	6269	13048
Independent reflections	2389	4146
Observed reflections (*I* ≥ 2*σ*)	1886	3366
*R_int_*	0.0839	0.039
*R (F)*	0.1161	0.099
*R_w_ (F^2^)*	0.3576	0.3512
No. of parameters, restraints	181, 0	272, 0
Goodness of fit	1.495	1.539
Δ*ρ*_max_, Δ*ρ*_min_ (eÅ^−3^)	0.654; −0.38	2.09; −1.034

## Data Availability

The data presented in this study are available on request from the corresponding author. The data are not publicly available due to privacy.

## Supplementary Materials

The following supporting information can be downloaded at: https://www.mdpi.com/article/10.3390/molecules27196379/s1, Figures S1–S34: NMR spectra of pure compounds; Figures S35–S46: UV spectra of pure compounds; Figures S47–S54: IR ATR spectra of pure compounds; Figures S55–S62: HRMS analyses, Figures S63–S66: Data for molecular docking; Figures S67–S72: Biometal chelating capability of cholinesterase inhibitory active pyranones; Tables S1–S4: Data for molecular docking; Tables S5 and S6: Crystal data. Table S7: Geometric parameters of π interactions in the compound trans-6. [53,54].

## Abbreviations

Aβ	aggregation of β-amyloid
AChE	Acetylcholinesterase
AD	Alzheimer’s disease
ACN	acetonitrile
AS	anionic site
BChE	butyrylcholinesterase
DCM	dichloromethane
DPPH	2,2′-diphenyl-1-picrylhydrazyl
HB	hydrogen bond
E	ether
EtOH	ethanol
NMR	nuclear magnetic resonance
EtOAc	ethyl acetate
MeOH	methanol
PAS	peripheric anionic site
PE	petroleum ether,
UV	ultraviolet spectrophotometry
s	singlet
d	doublet
t	triplet
q	quartet
dd	doublet of doublets
m	multiplet
