# Supplementary material for: Resveratrol–Maltol and Resveratrol–Thiophene Hybrids as Cholinesterase Inhibitors and Antioxidants: Synthesis, Biometal Chelating Capability and Crystal Structure
Source: Molecules. 2022 Sep 27;27(19):6379. doi: 10.3390/molecules27196379 (PMC9573353; doi:10.3390/molecules27196379)
Supplement: Supplementary file 1 [file molecules-27-06379-s001.zip › molecules-1923628-supplementary.pdf]

Electronic Supporting Information

Resveratrol-Maltol and Resveratrol-Thiophene Hybrids as Cholinesterase Inhibitors and Antioxidants: Synthesis, Biometal Chelating Capability and Crystal Structure

Milena Mlakić[1§], Lajos Fodor[2§], Ilijana Odak[3*], Ottó Horváth[2], Marija Jelena Lovrić[4], Danijela Barić[4], Valentina Milašinović[5], Krešimir Molčanov[5], Željko Marinić[6], Zlata Lasić[7] and Irena Škorić[1*]

[1]Department of Organic Chemistry, Faculty of Chemical Engineering and Technology, University of Zagreb, Marulićev trg 19, HR-10 000 Zagreb, Croatia

[2]Department of General and Inorganic Chemistry, Institute of Chemistry, Faculty of Engineering, University of Pannonia, P.O.B. 158, Veszprém H-8201, Hungary

[3]Department of Chemistry, Faculty of Science and Education, University of Mostar, Matice hrvatske bb, 88 000 Mostar, Bosnia and Herzegovina

[4]Group for Computational Life Sciences, Division of Physical Chemistry, Ruđer Bošković Institute, Bijenička cesta 54, HR-10 000 Zagreb, Croatia

[5]Division of Physical Chemistry, Rudjer Bošković Institute, Bijenička cesta 54, HR-10 000 Zagreb, Croatia

[6]NMR Center, Rudjer Bošković Institute, Bijenička cesta 54, HR-10 000 Zagreb, Croatia

[7]Teva api Chemical R&D, Pliva, Prilaz Baruna Filipovića 25, HR-10 000, Zagreb, Croatia

*Corresponding authors: Prof Ilijana Odak; ilijana.odak@fpmoz.sum.ba; Prof Irena Škorić; iskoric@fkit.hr

[§]These authors contributed equally.

Content:

1. NMR spectra of pure compounds (Figures S1-S34)
2. UV spectra of pure compounds (Figures S35-S46)
3. IR ATR spectra of pure compounds (Figures S47-S54)
4. HRMS analyses (Figures S55-S62)
5. Molecular docking (Tables S1-S4, Figures S63-S66)
6. Biometal chelating capability of cholinesterase inhibitory active pyranones (Figures S67-S72)

7. Crystal data (Tables S5-S7)

1. NMR spectra of pure compounds

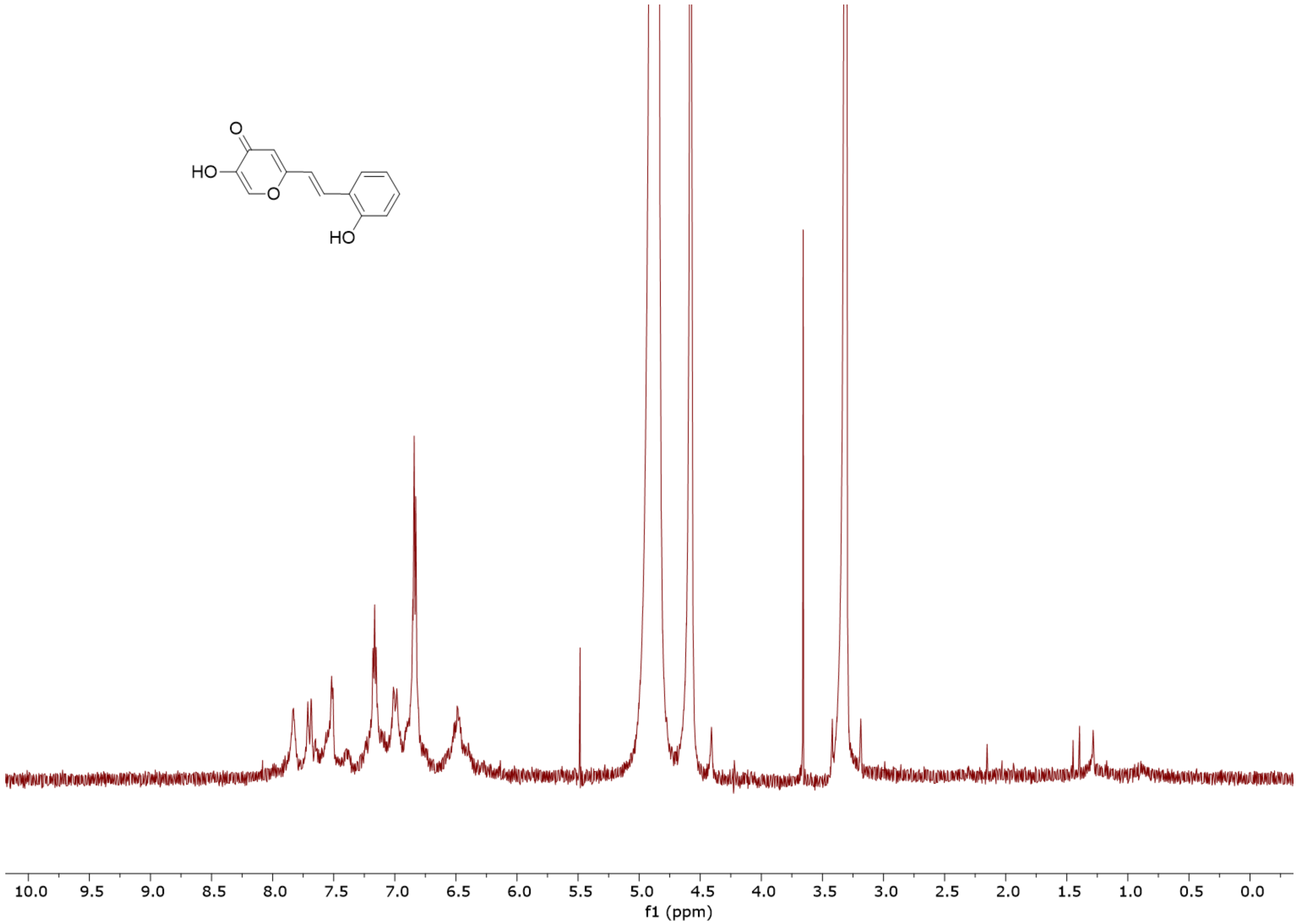

Figure S1. ^{1}H NMR spectrum (CD_3OD) of (*E*)-5-hydroxy-2-(2-hydroxystyryl)-4*H*-pyran-4-one (*trans*-**1**).

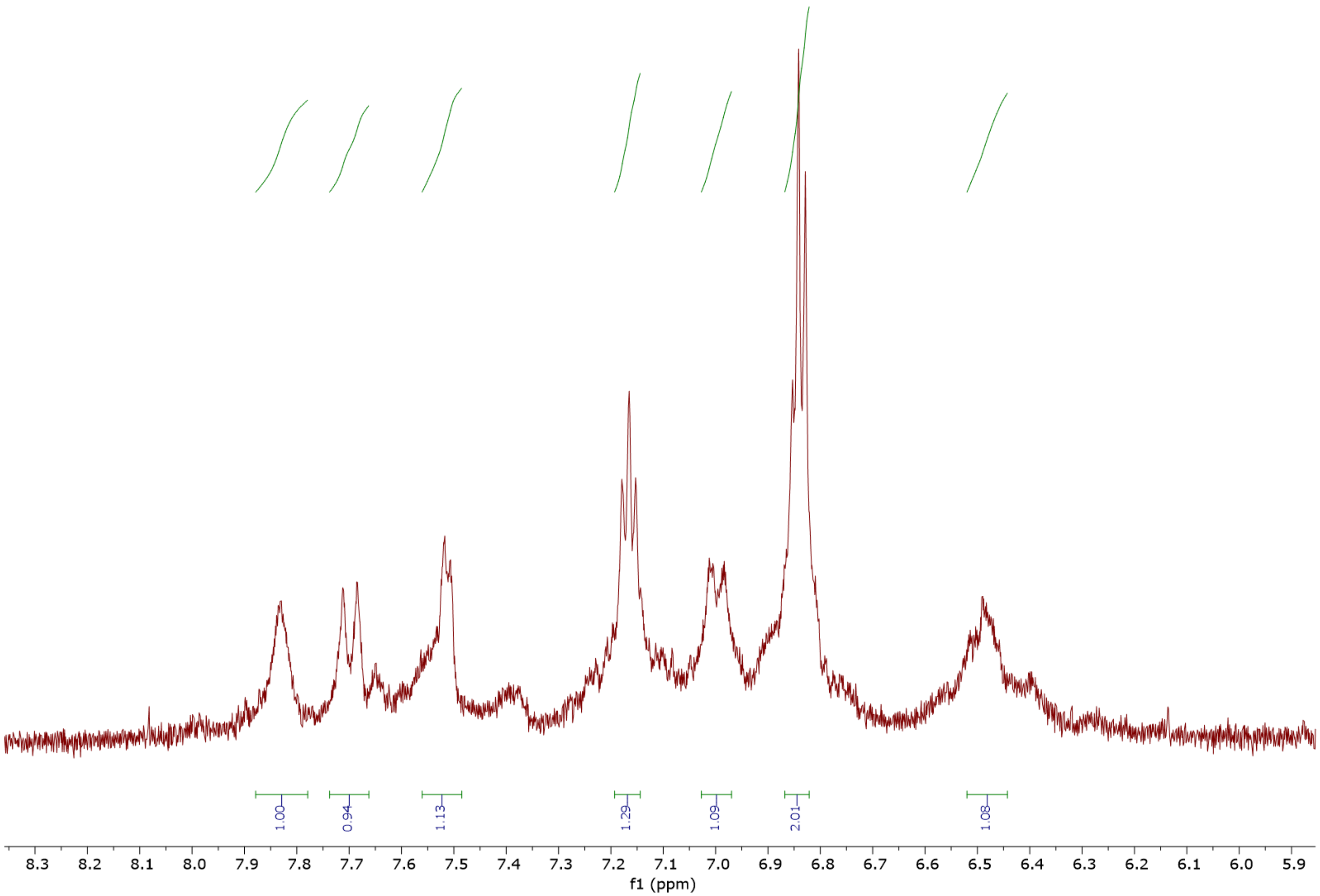

Figure S2. Part of the ^{1}H NMR spectrum (CD_3OD) of (*E*)-5-hydroxy-2-(2-hydroxystyryl)-4*H*-pyran-4-one (*trans*-**1**).

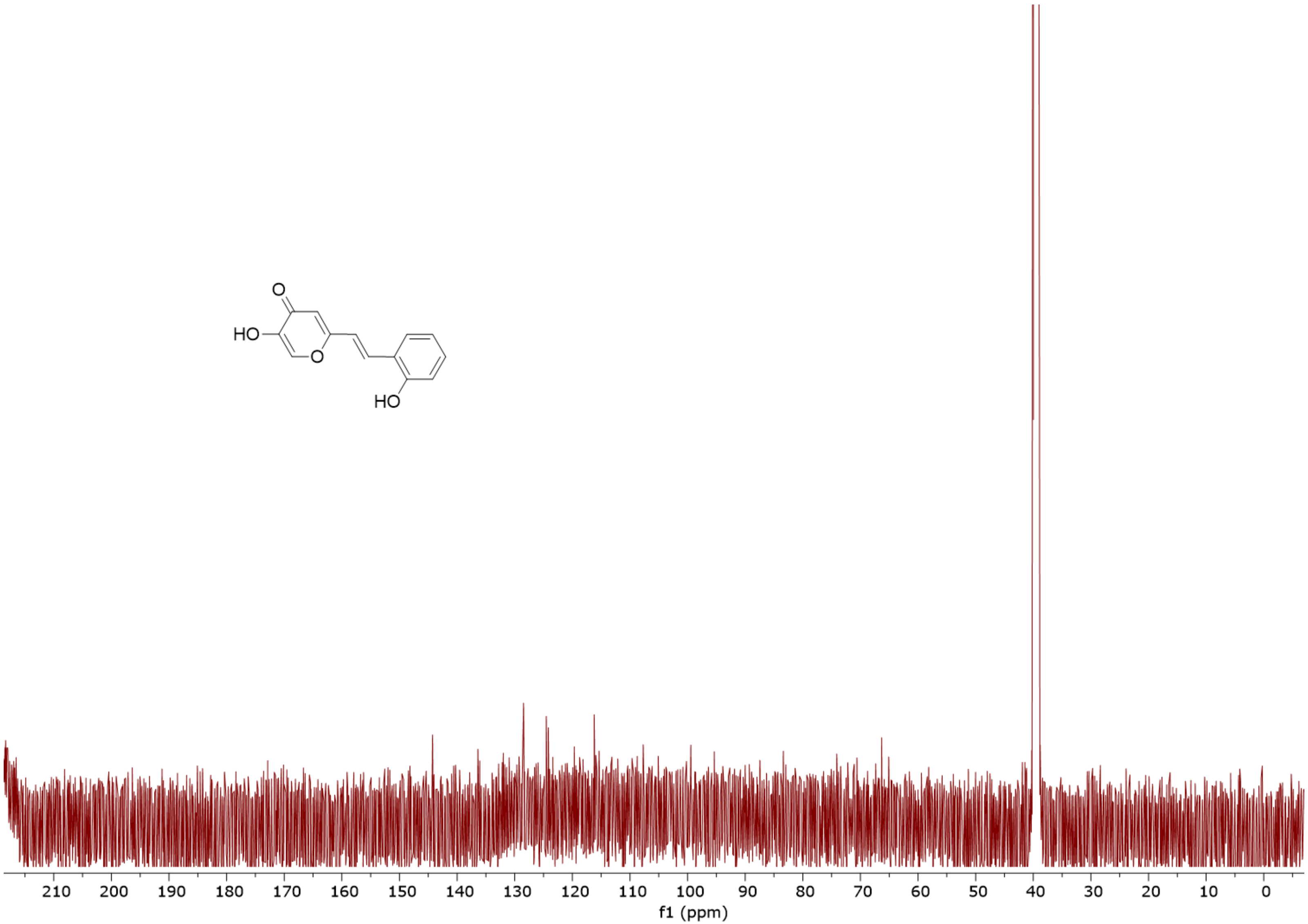

Figure S3. ^{13}C NMR spectrum (DMSO-d6) of (*E*)-5-hydroxy-2-(2-hydroxystyryl)-4*H*-pyran-4-one (*trans*-**1**).

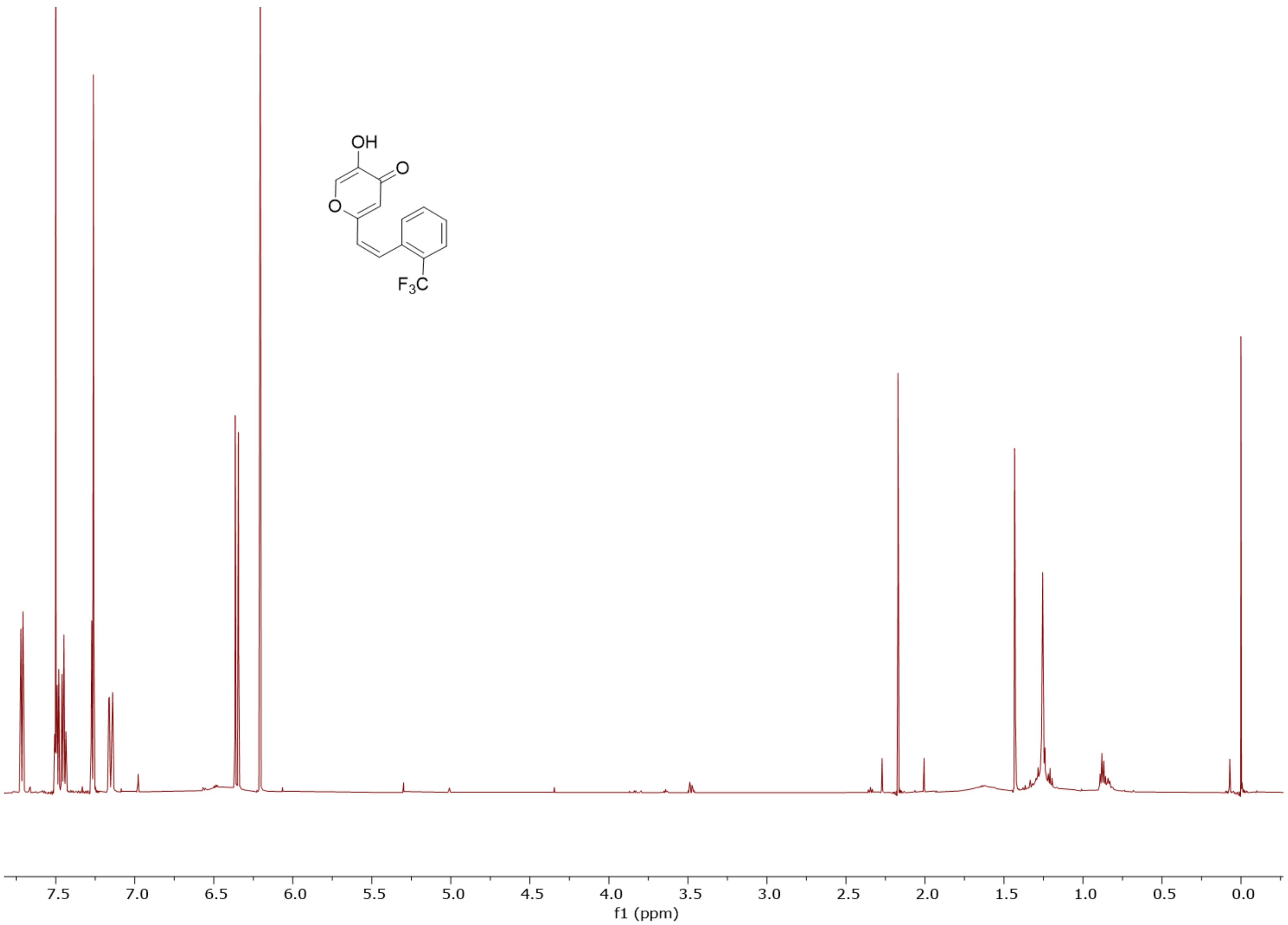

Figure S4. ^{1}H NMR spectrum ($CDCl_3$) of (*Z*)-5-hydroxy-2-(2-(trifluoromethyl)styryl)-4*H*-pyran-4-one (*cis*-**2**).

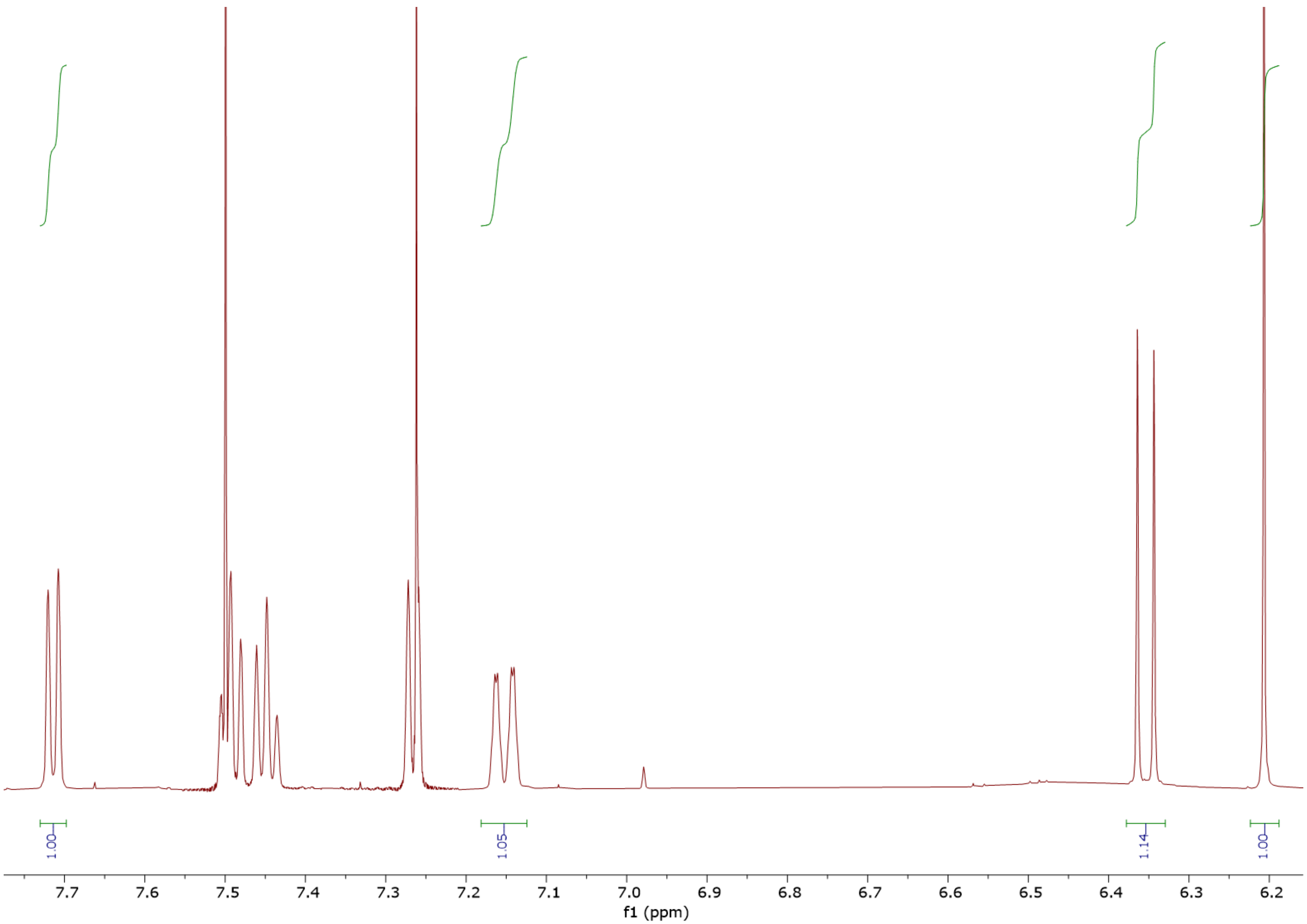

Figure S5. Part of the ^{1}H NMR spectrum ($CDCl_3$) of (*Z*)-5-hydroxy-2-(2-(trifluoromethyl)styryl)-4*H*-pyran-4-one (*cis*-**2**).

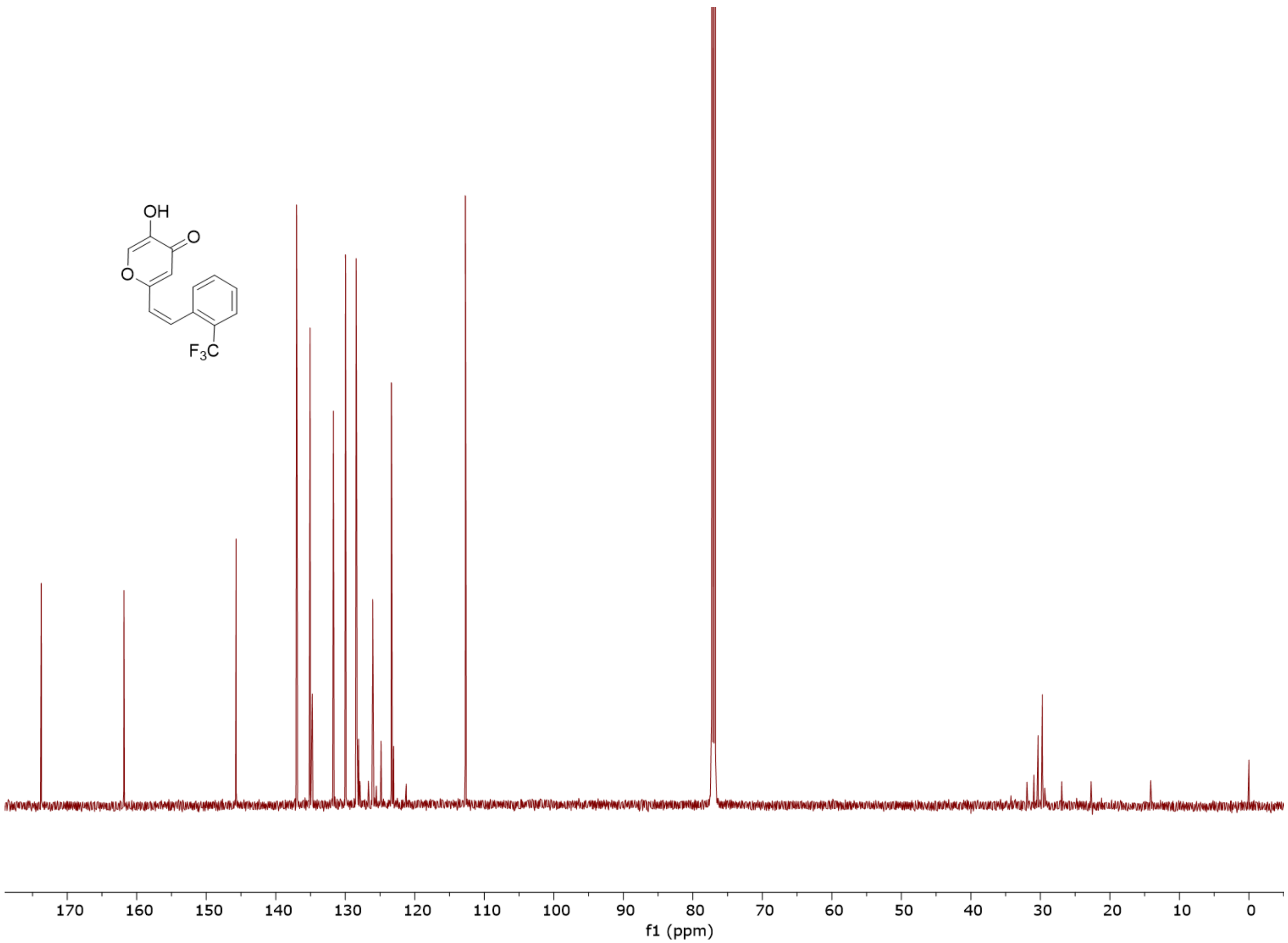

Figure S6. ^{13}C NMR spectrum ($CDCl_3$) of (*Z*)-5-hydroxy-2-(2-(trifluoromethyl)styryl)-4*H*-pyran-4-one (*cis*-**2**).

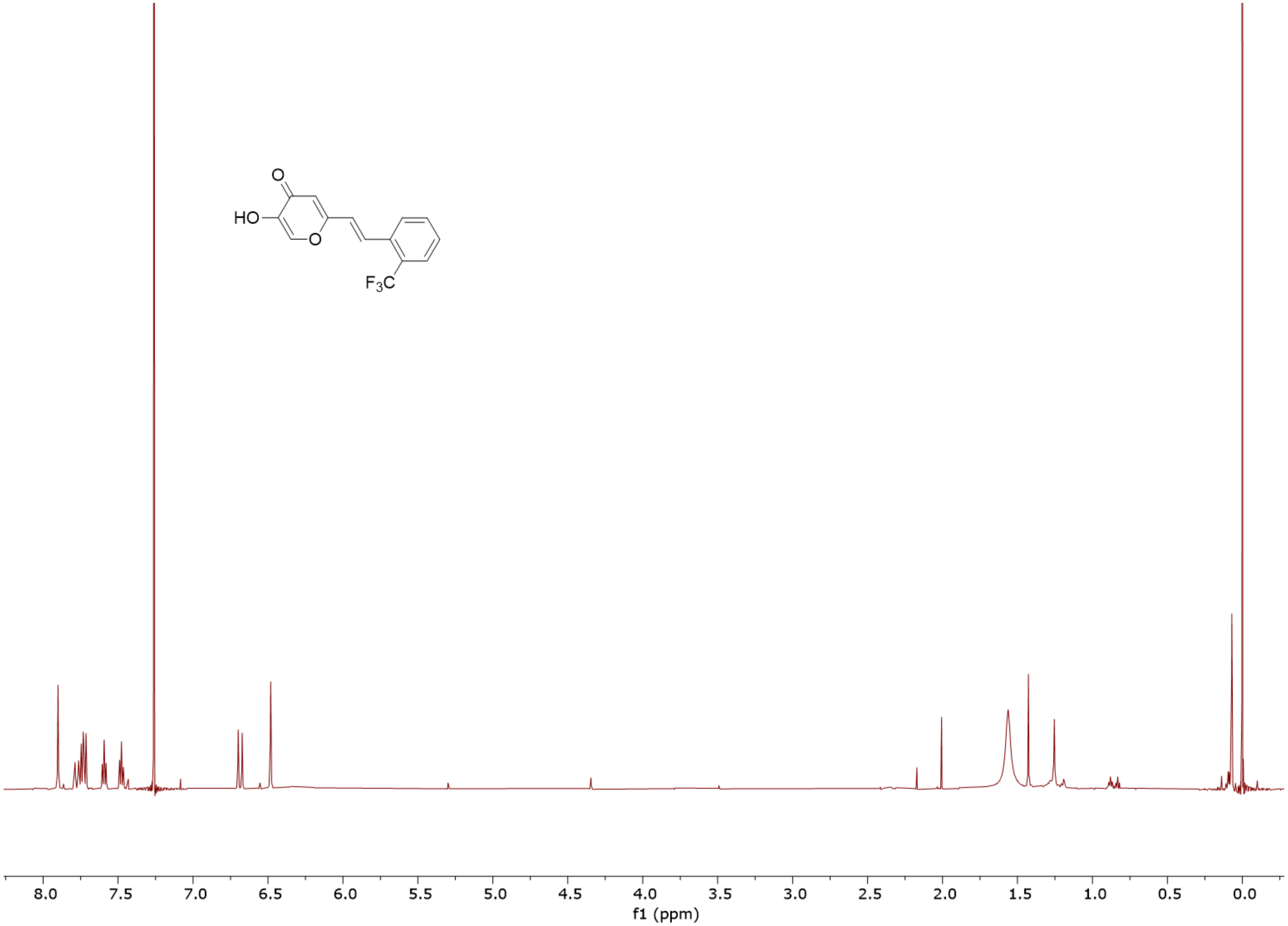

Figure S7. ^{1}H NMR spectrum ($CDCl_3$) of (*E*)-5-hydroxy-2-(2-(trifluoromethyl)styryl)-4*H*-pyran-4-one (*trans*-**2**).

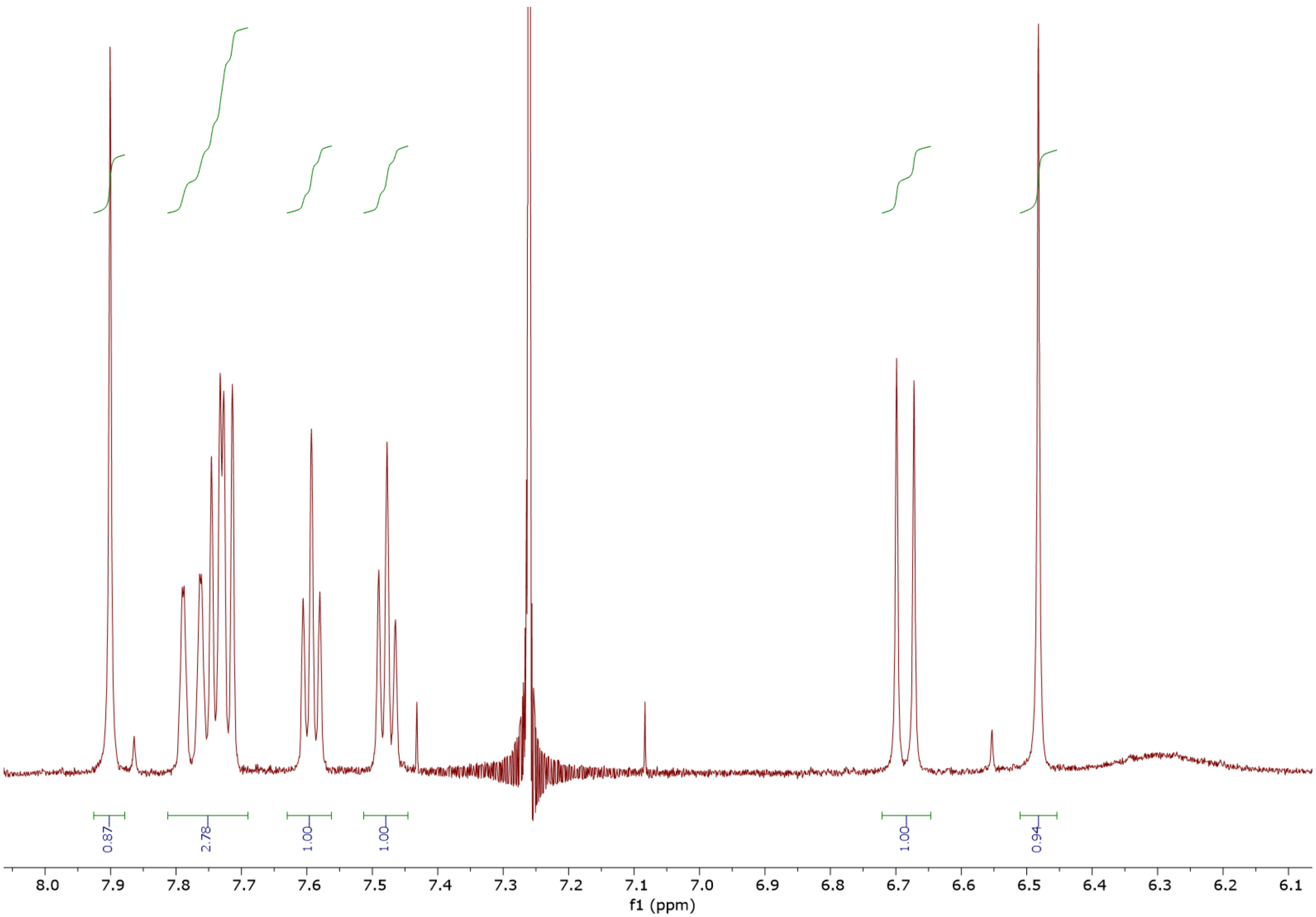

Figure S8. Part of the ^{1}H NMR spectrum ($CDCl_3$) of (*E*)-5-hydroxy-2-(2-(trifluoromethyl)styryl)-4*H*-pyran-4-one (*trans*-**2**).

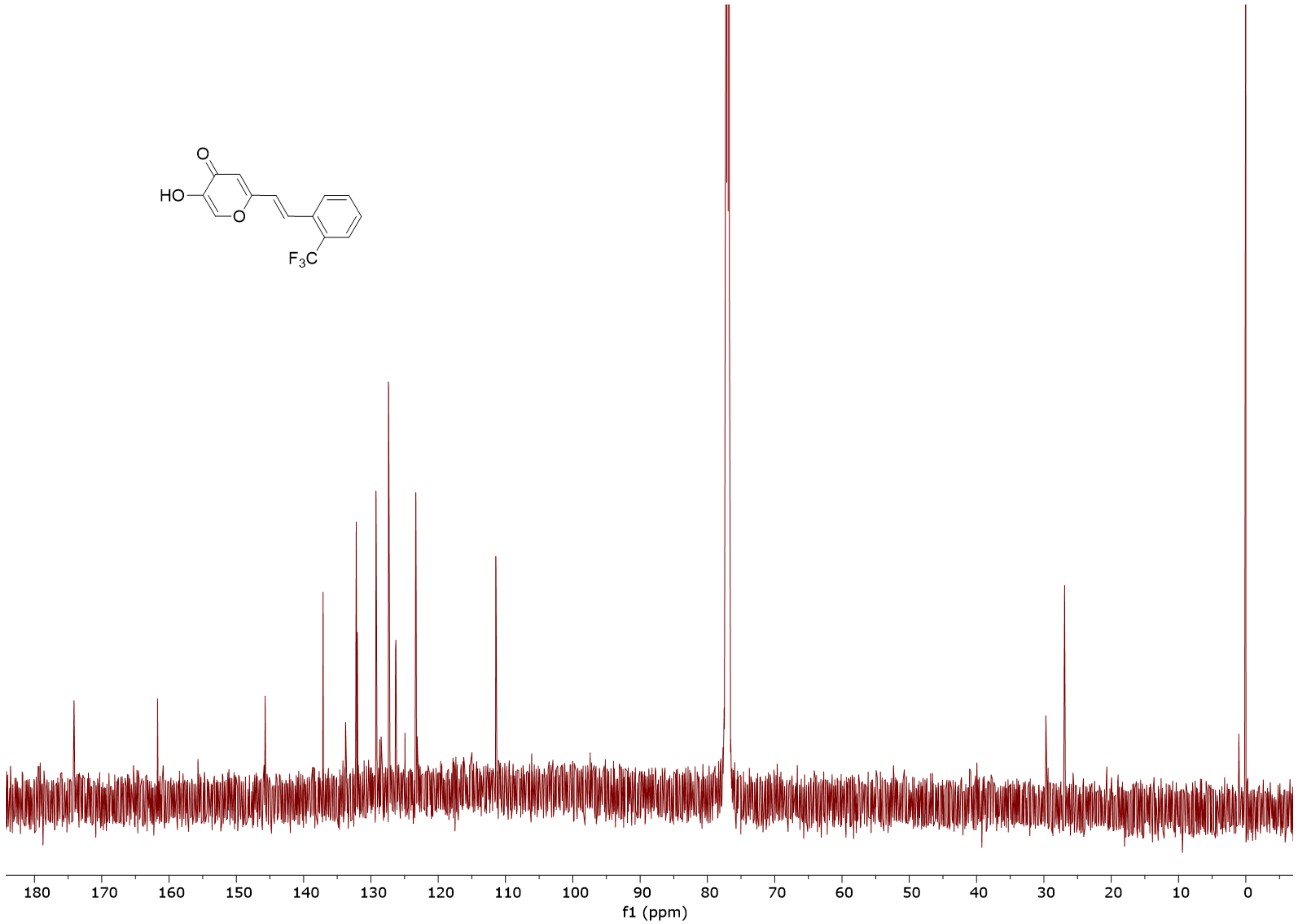

Figure S9. ^{13}C NMR spectrum ($CDCl_3$) of (*E*)-5-hydroxy-2-(2-(trifluoromethyl)styryl)-4*H*-pyran-4-one (*trans*-**2**).

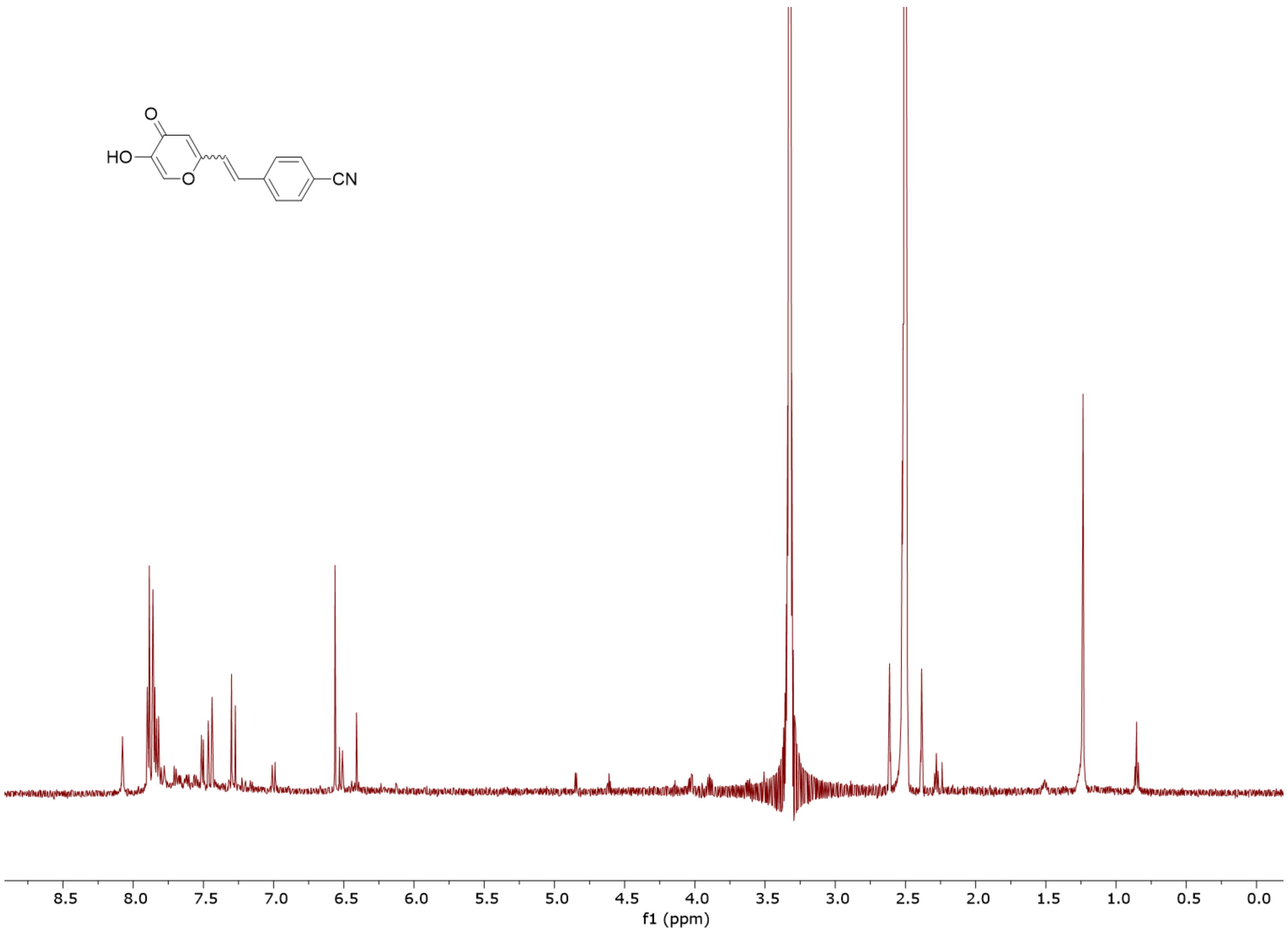

Figure S10. ^{1}H NMR spectrum (DMSO-d6) of 4-(2-(5-hydroxy-4-oxo-4*H*-pyran-2-yl)vinyl)benzonitrile **3** (mixture of isomers).

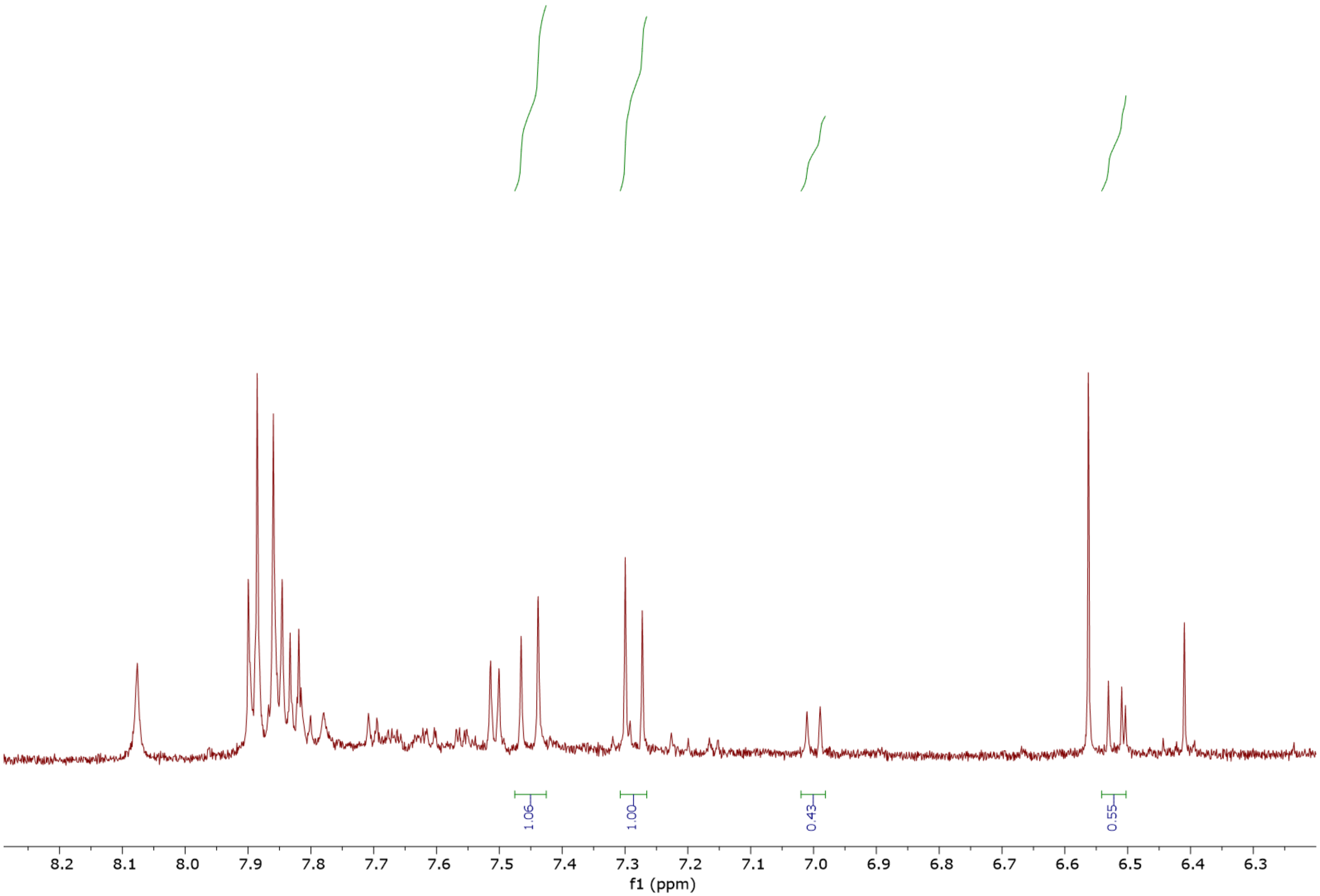

Figure S11. Part of the ^{1}H NMR spectrum (DMSO-d6) of 4-(2-(5-hydroxy-4-oxo-4*H*-pyran-2-yl)vinyl)benzonitrile **3** (mixture of isomers).

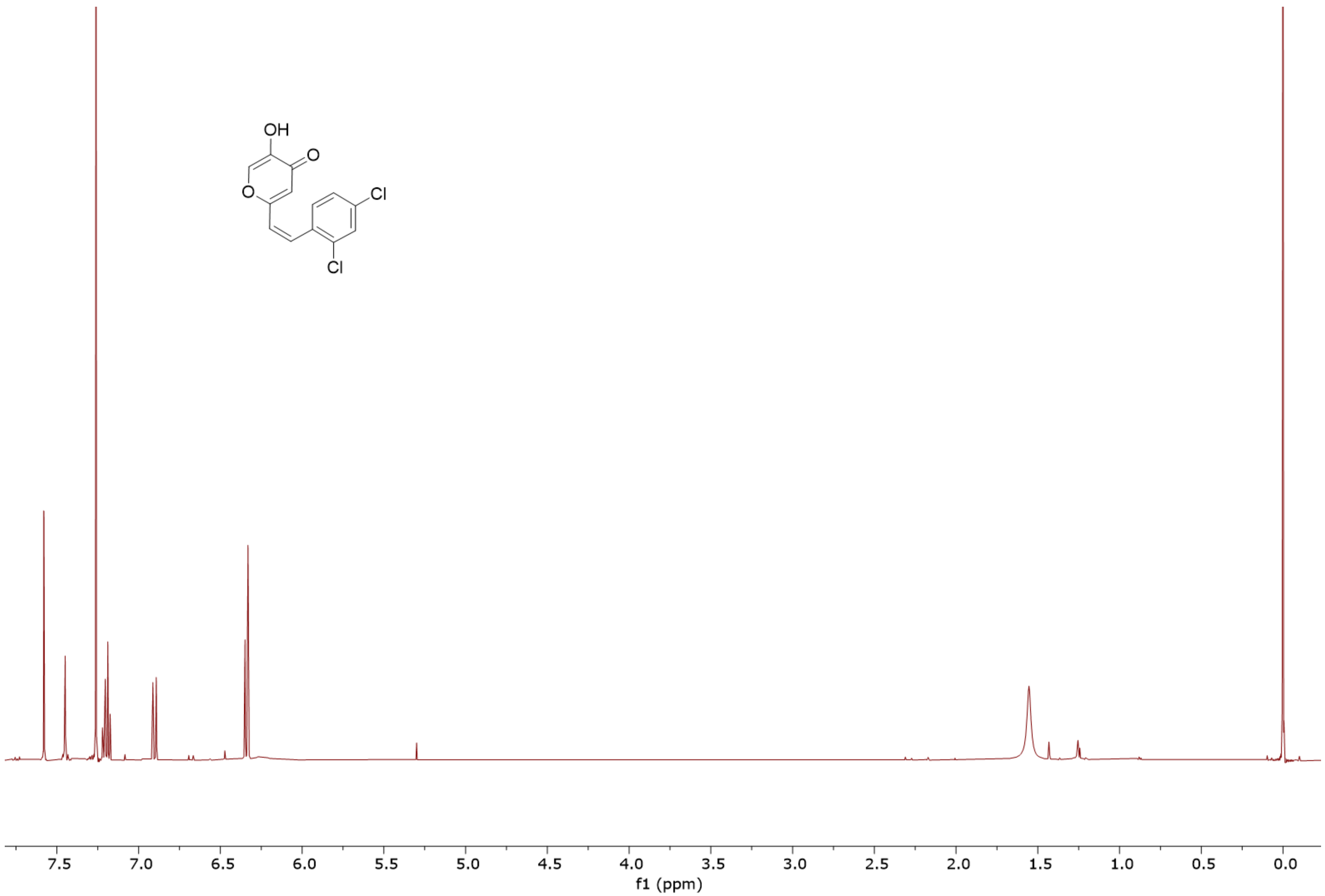

Figure S12. ^{1}H NMR spectrum ($CDCl_3$) of (*Z*)-2-(2,4-dichlorostyryl)-5-hydroxy-4*H*-pyran-4-one (*cis*-**4**).

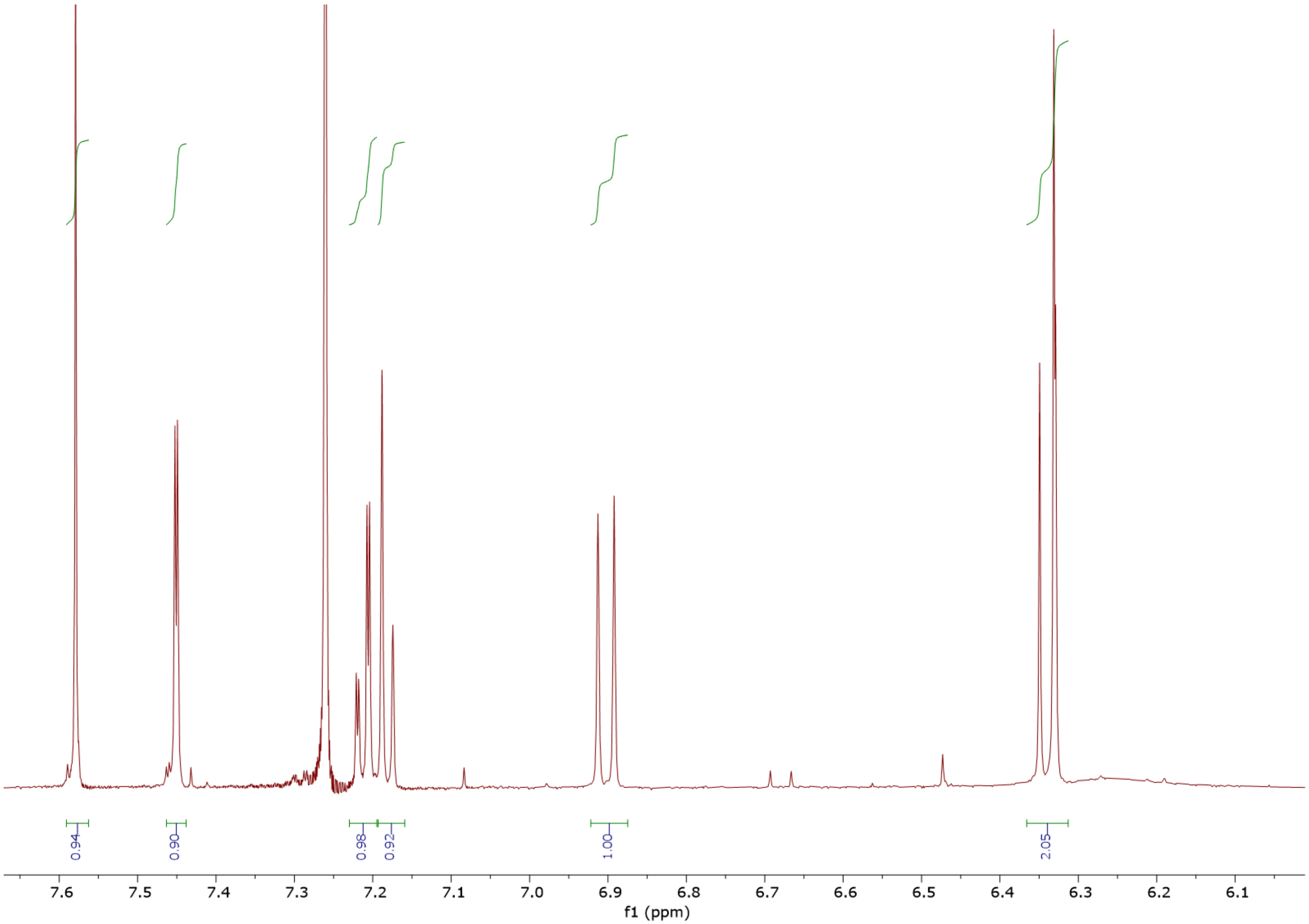

Figure S13. Part of the ^{1}H NMR spectrum ($CDCl_3$) of (*Z*)-2-(2,4-dichlorostyryl)-5-hydroxy-4*H*-pyran-4-one (*cis*-**4**).

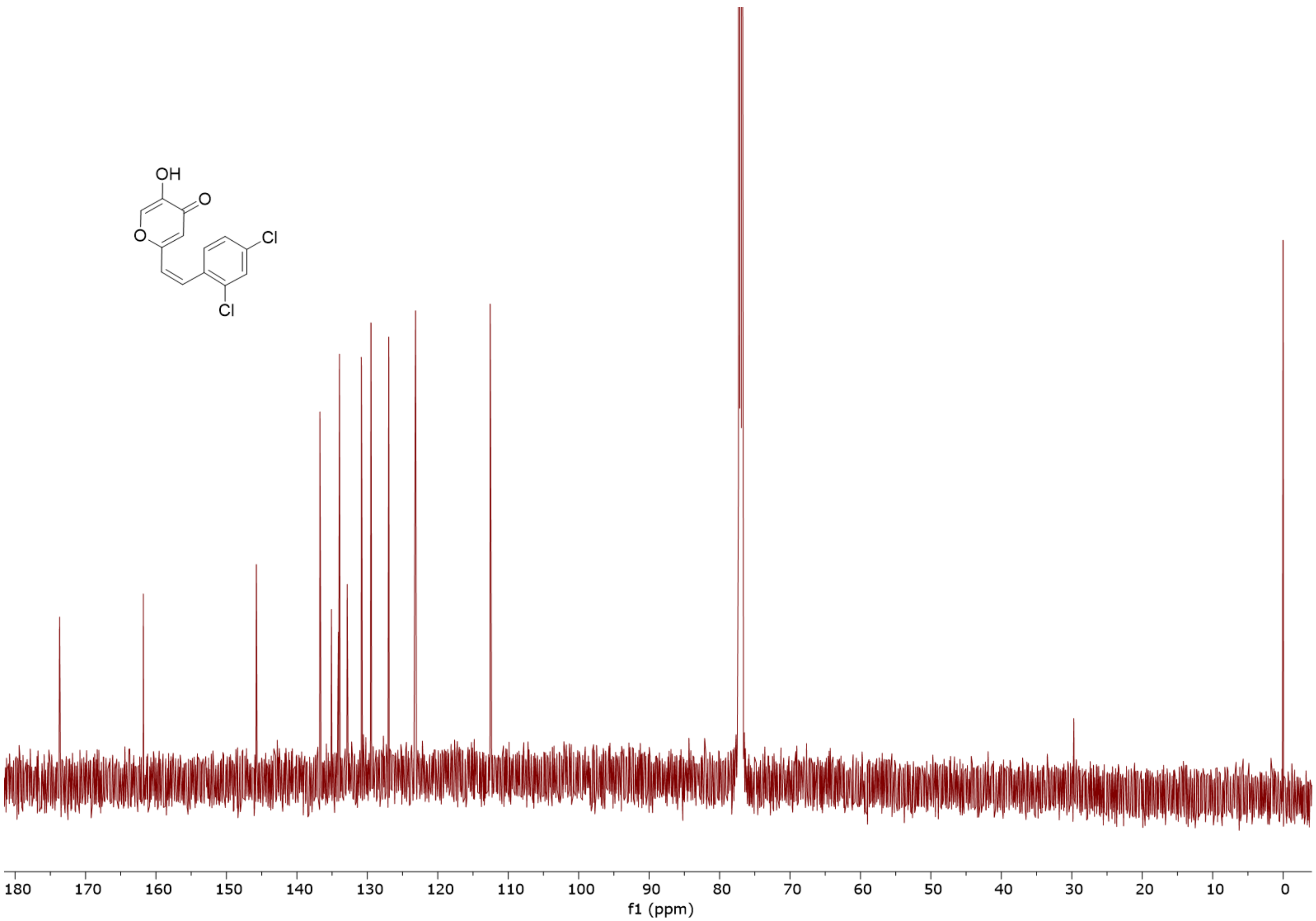

Figure S14. ^{13}C NMR spectrum ($CDCl_3$) of (*Z*)-2-(2,4-dichlorostyryl)-5-hydroxy-4*H*-pyran-4-one (*cis*-**4**).

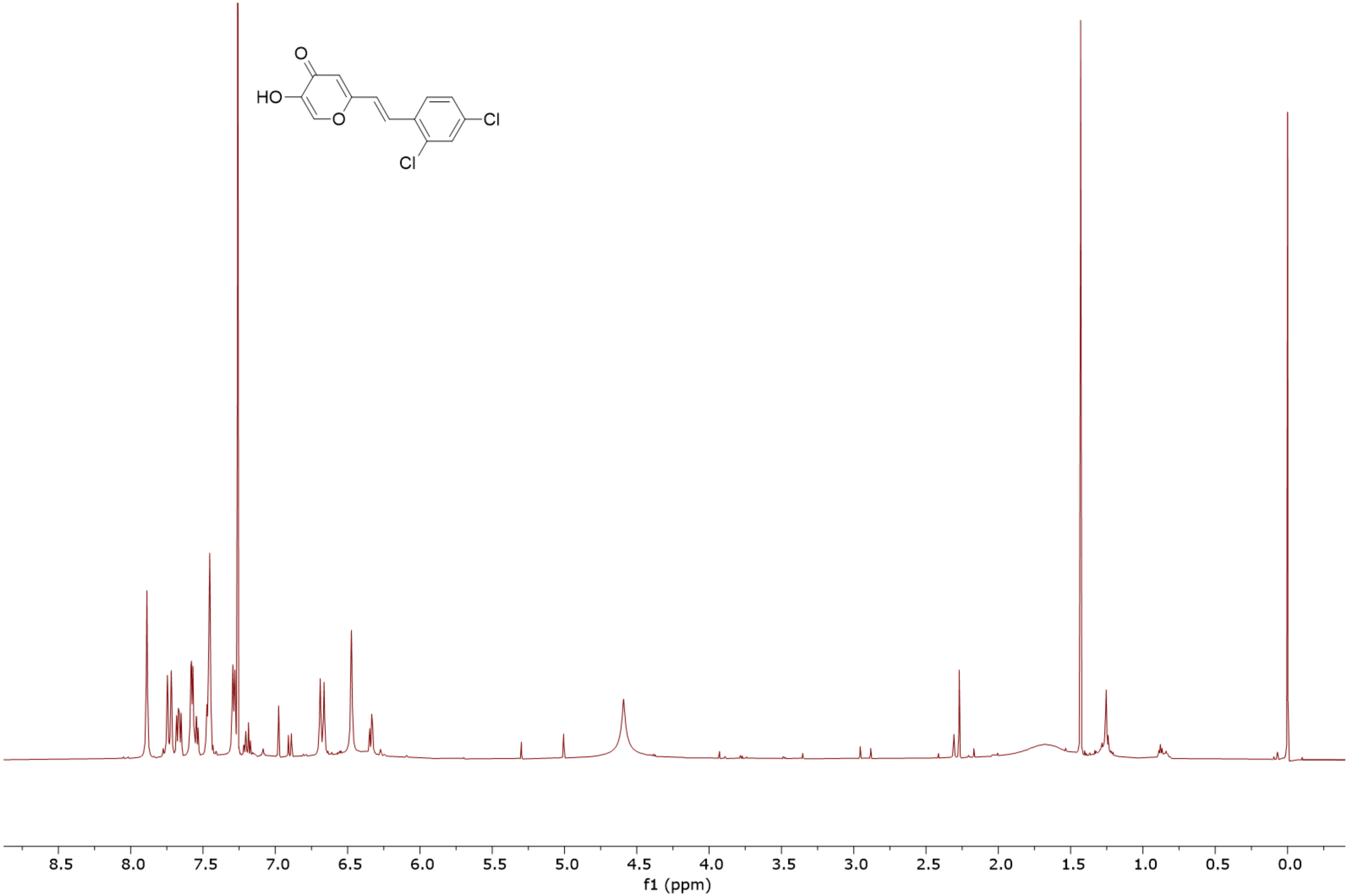

Figure S15. ^{1}H NMR spectrum ($CDCl_3$) of (*E*)-2-(2,4-dichlorostyryl)-5-hydroxy-4*H*-pyran-4-one (*trans*-**4**).

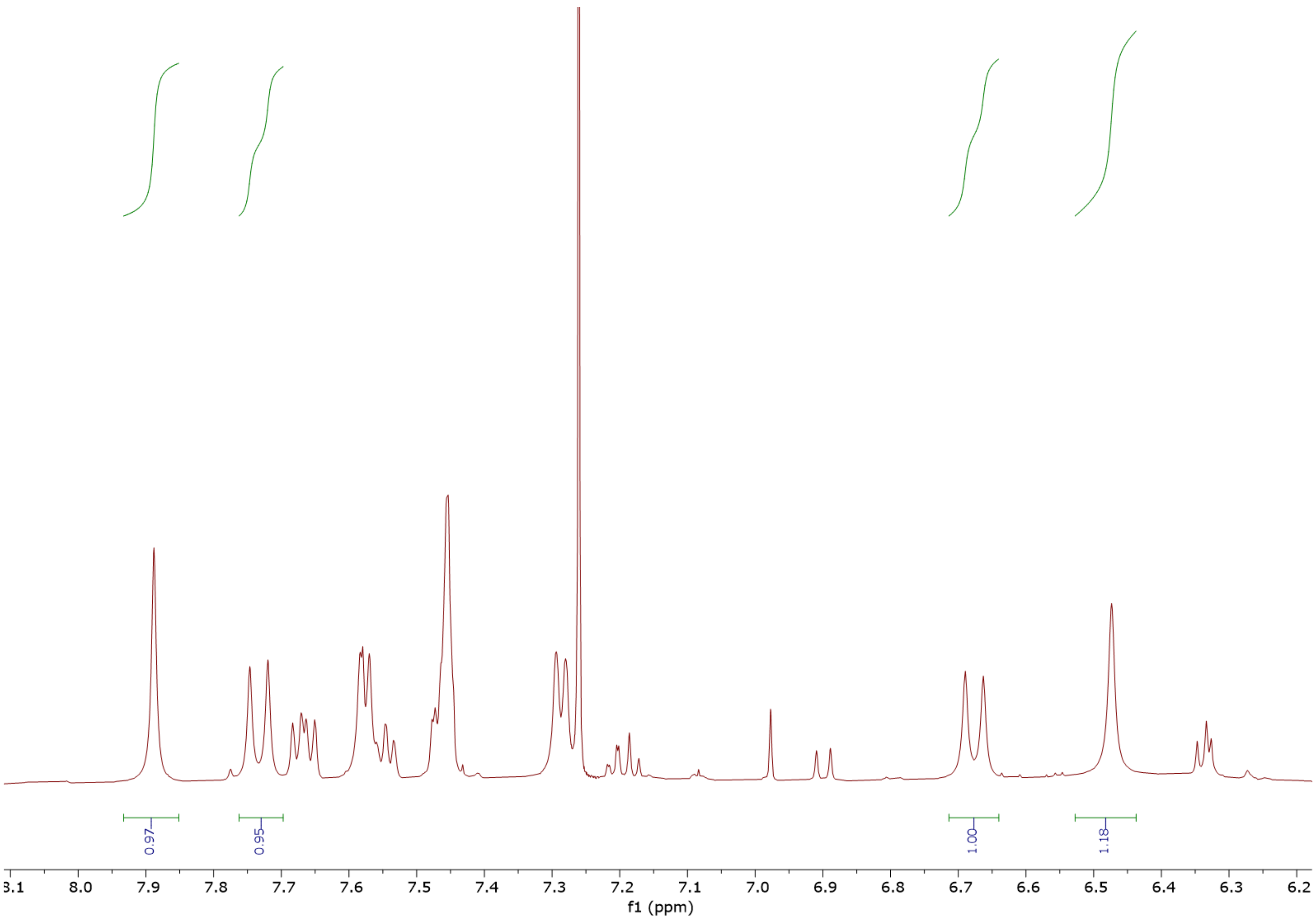

Figure S16. Part of the ^{1}H NMR spectrum ($CDCl_3$) of (*E*)-2-(2,4-dichlorostyryl)-5-hydroxy-4*H*-pyran-4-one (*trans*-**4**).

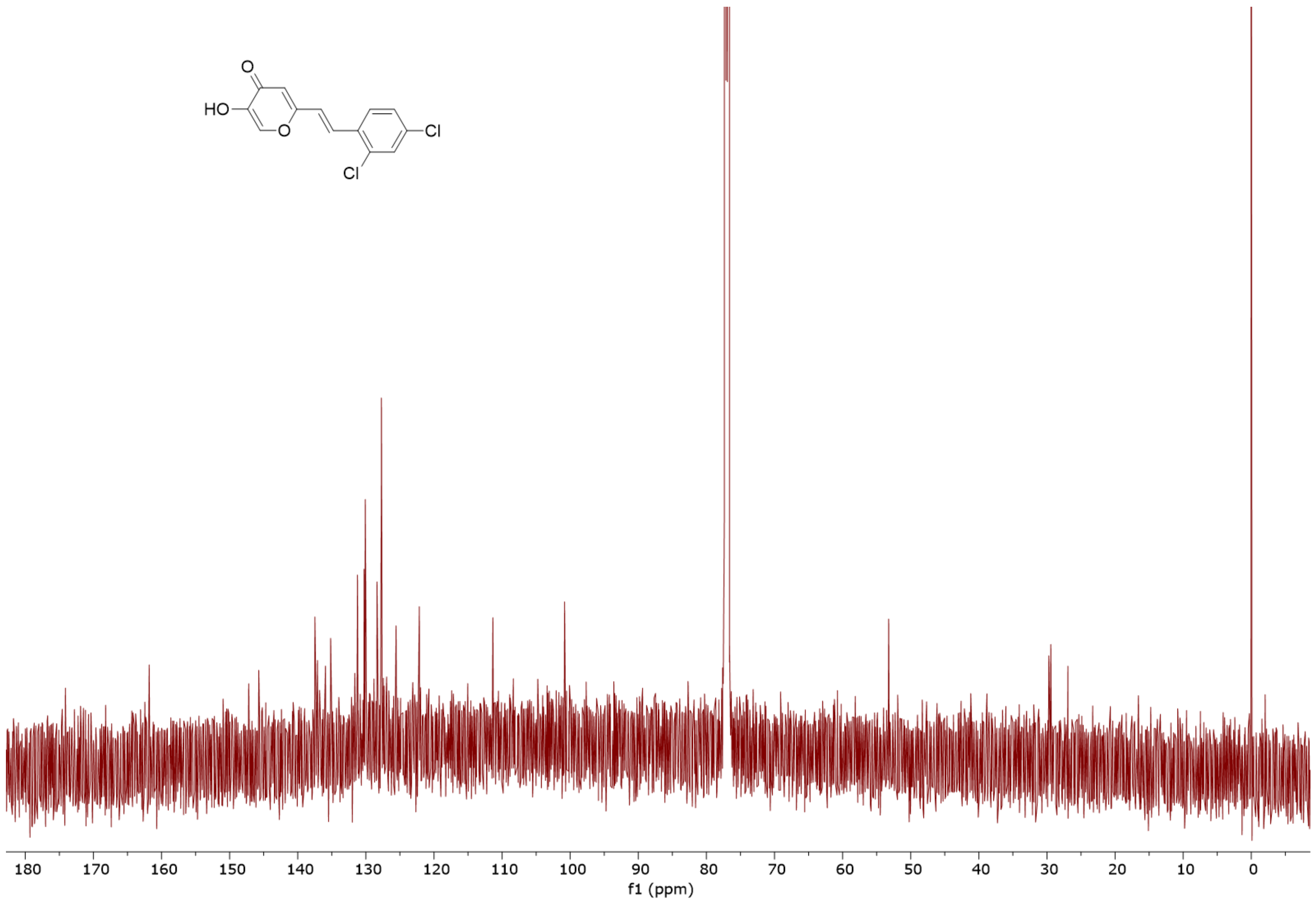

Figure S17. ^{13}C NMR spectrum ($CDCl_3$) of (*E*)-2-(2,4-dichlorostyryl)-5-hydroxy-4*H*-pyran-4-one (*trans*-**4**).

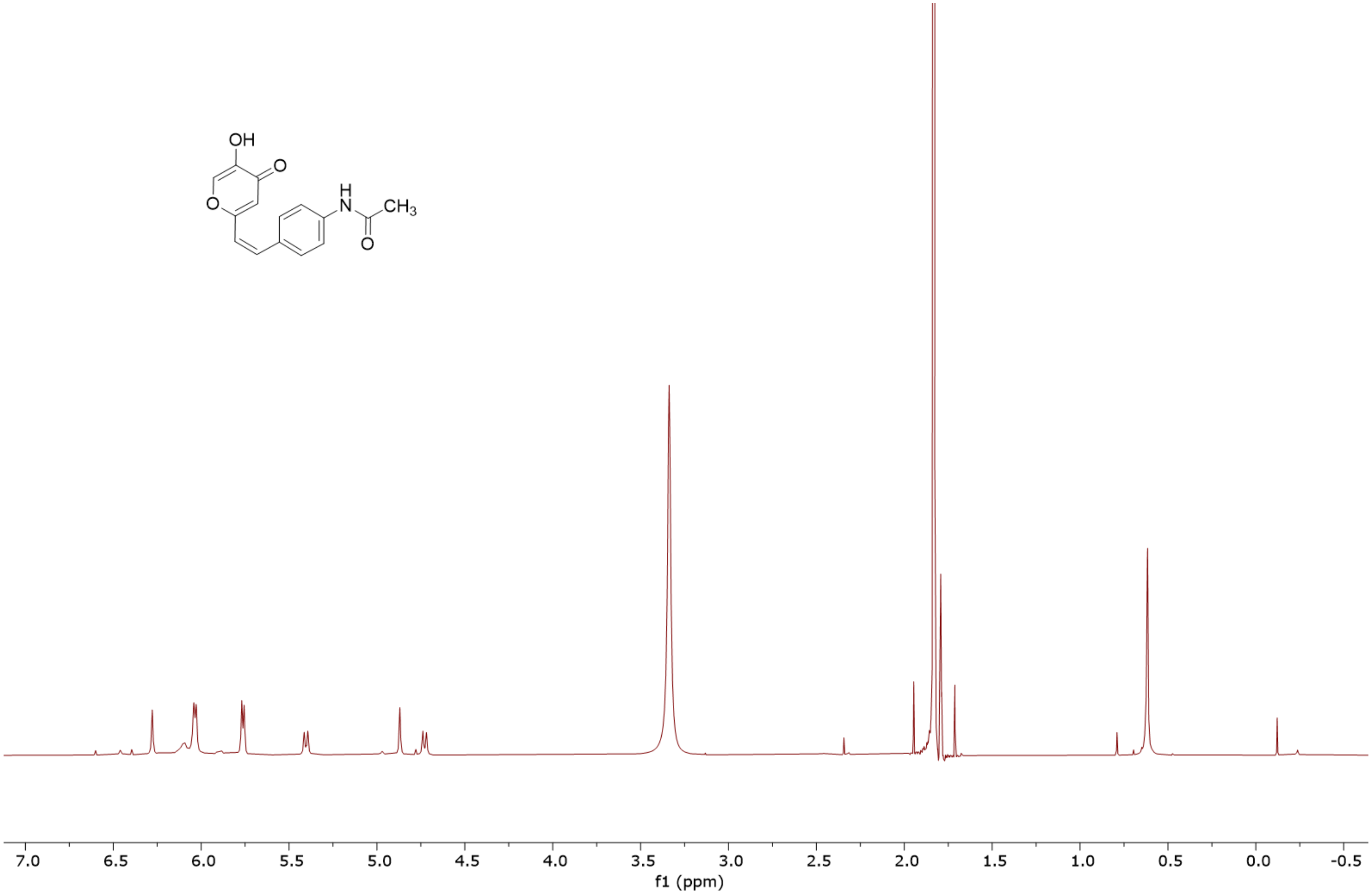

Figure S18. ^{1}H NMR spectrum (CD$_3$OD) of (*Z*)-*N*-(4-(2-(5-hydroxy-4-oxo-4*H*-pyran-2-yl)vinyl)phenyl)acetamide (*cis*-**5**).

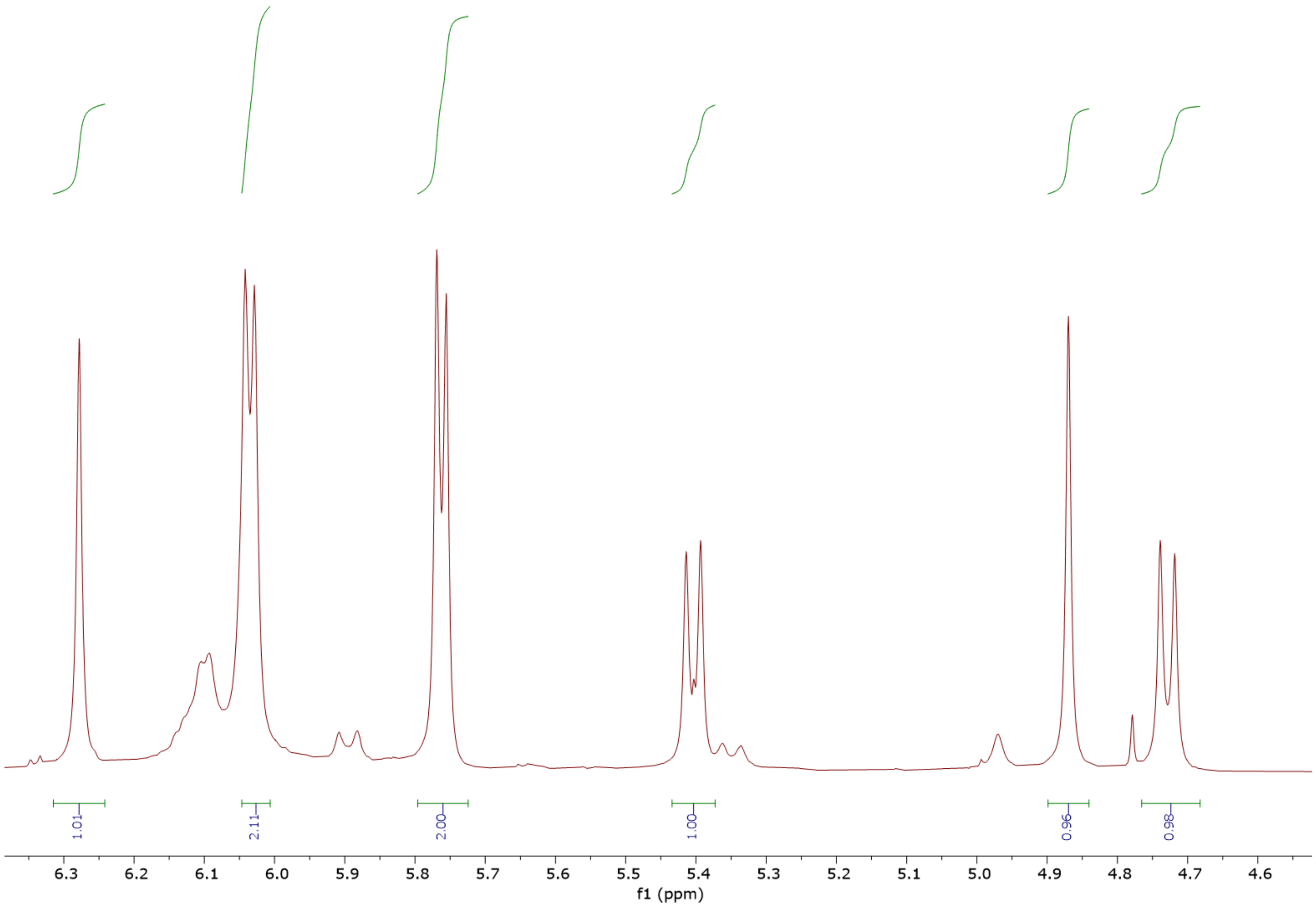

Figure S19. Part of the ^{1}H NMR spectrum (CD_3OD) of (*Z*)-*N*-(4-(2-(5-hydroxy-4-oxo-4*H*-pyran-2-yl)vinyl)phenyl)acetamide (*cis*-**5**).

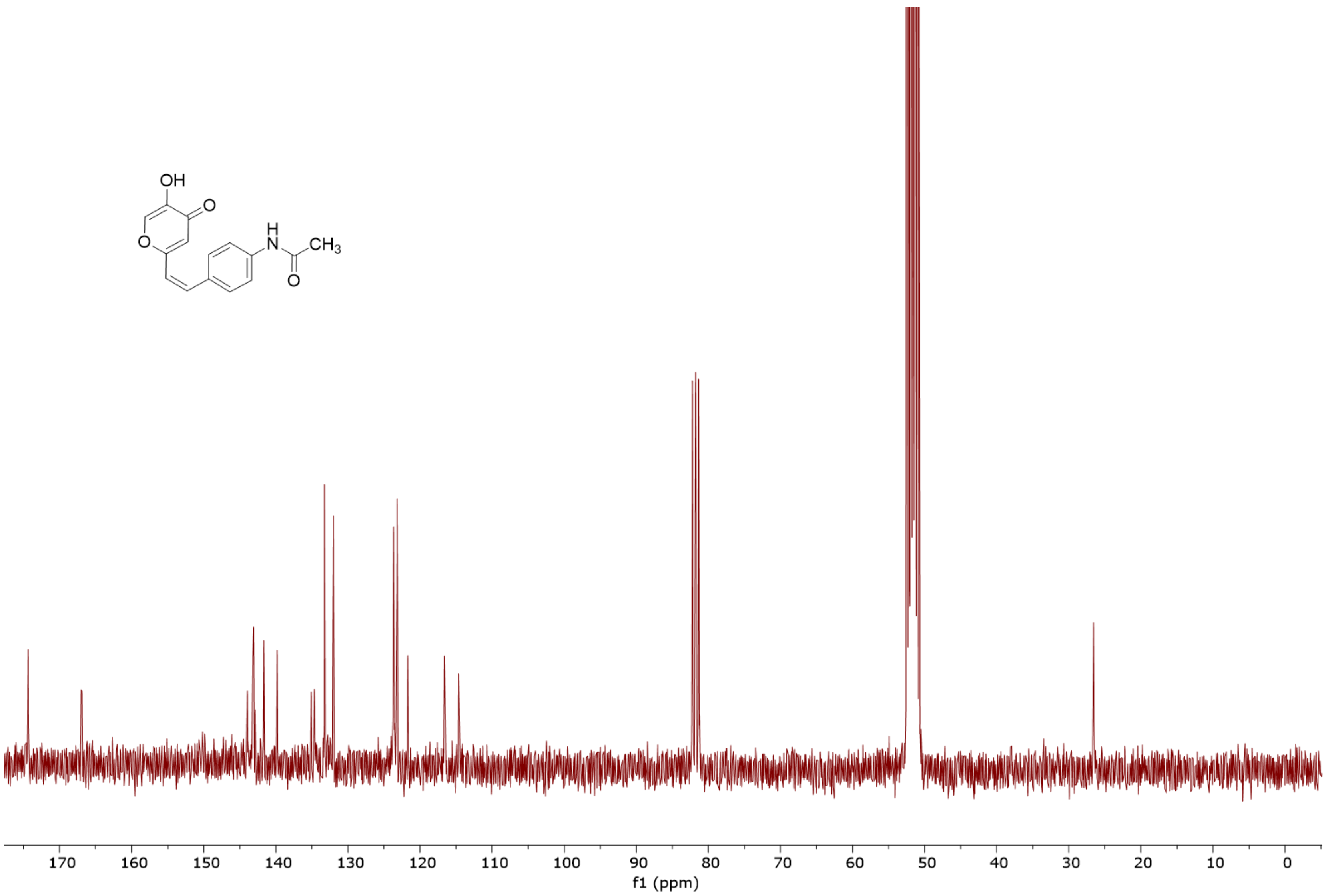

Figure S20. ^{13}C NMR spectrum (CD_3OD) of (*Z*)-*N*-(4-(2-(5-hydroxy-4-oxo-4*H*-pyran-2-yl)vinyl)phenyl)acetamide (*cis*-**5**).

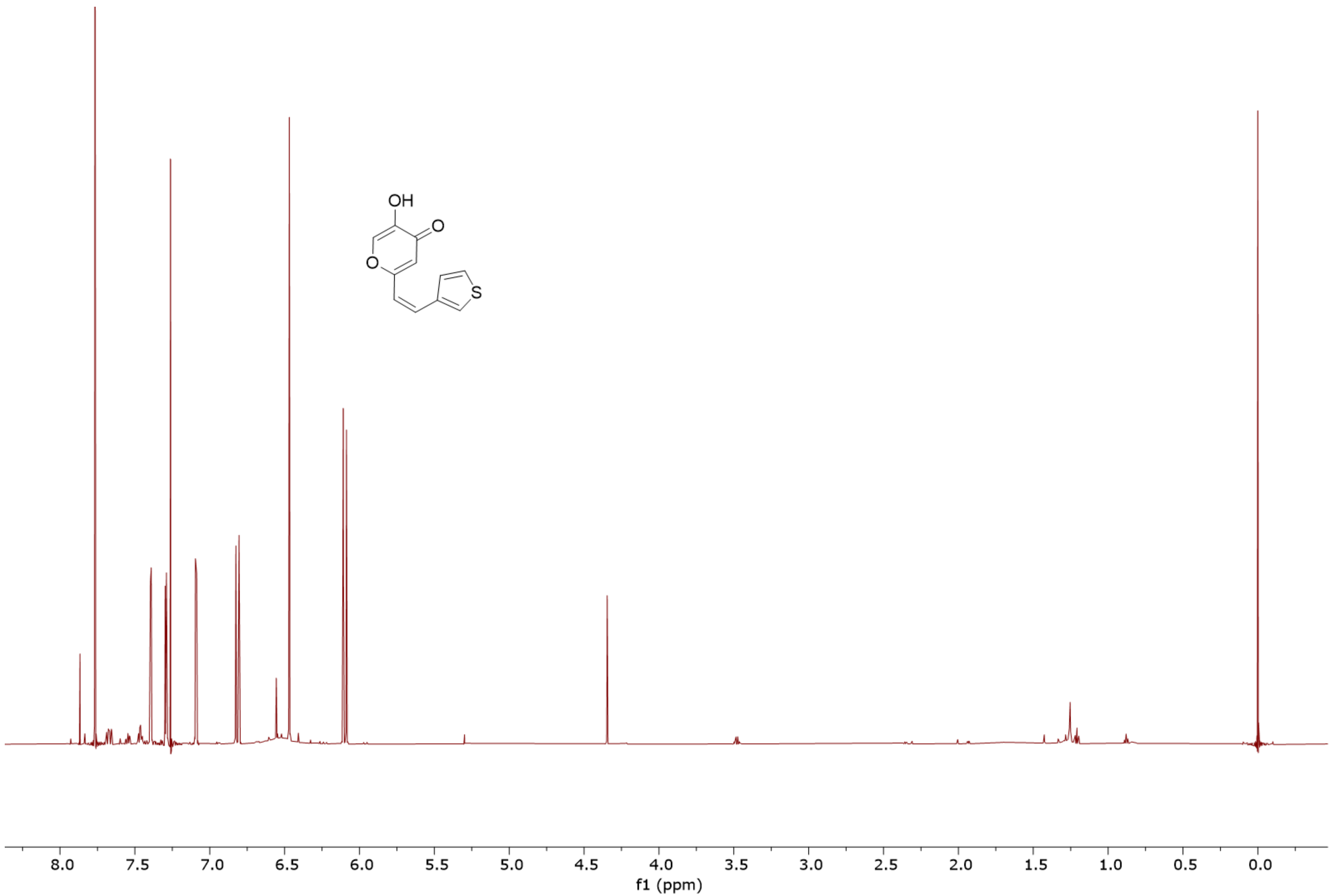

Figure S21. ^{1}H NMR spectrum ($CDCl_3$) of (*Z*)-5-hydroxy-2-(2-(thiophen-3-yl)vinyl)-4*H*-pyran-4-one (*cis*-**6**).

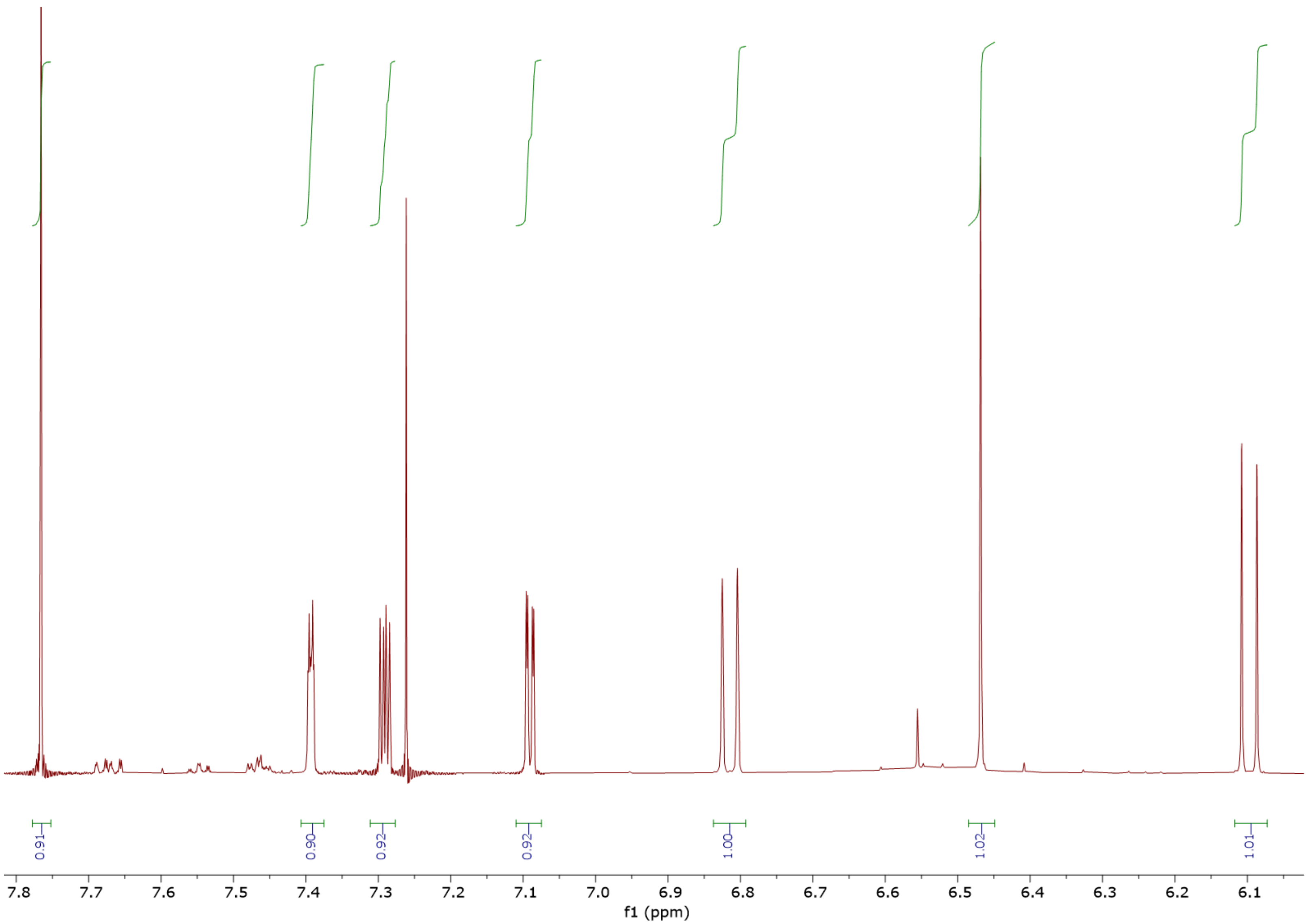

Figure S22. Part of the ^{1}H NMR spectrum ($CDCl_3$) of (*Z*)-5-hydroxy-2-(2-(thiophen-3-yl)vinyl)-4*H*-pyran-4-one (*cis*-**6**).

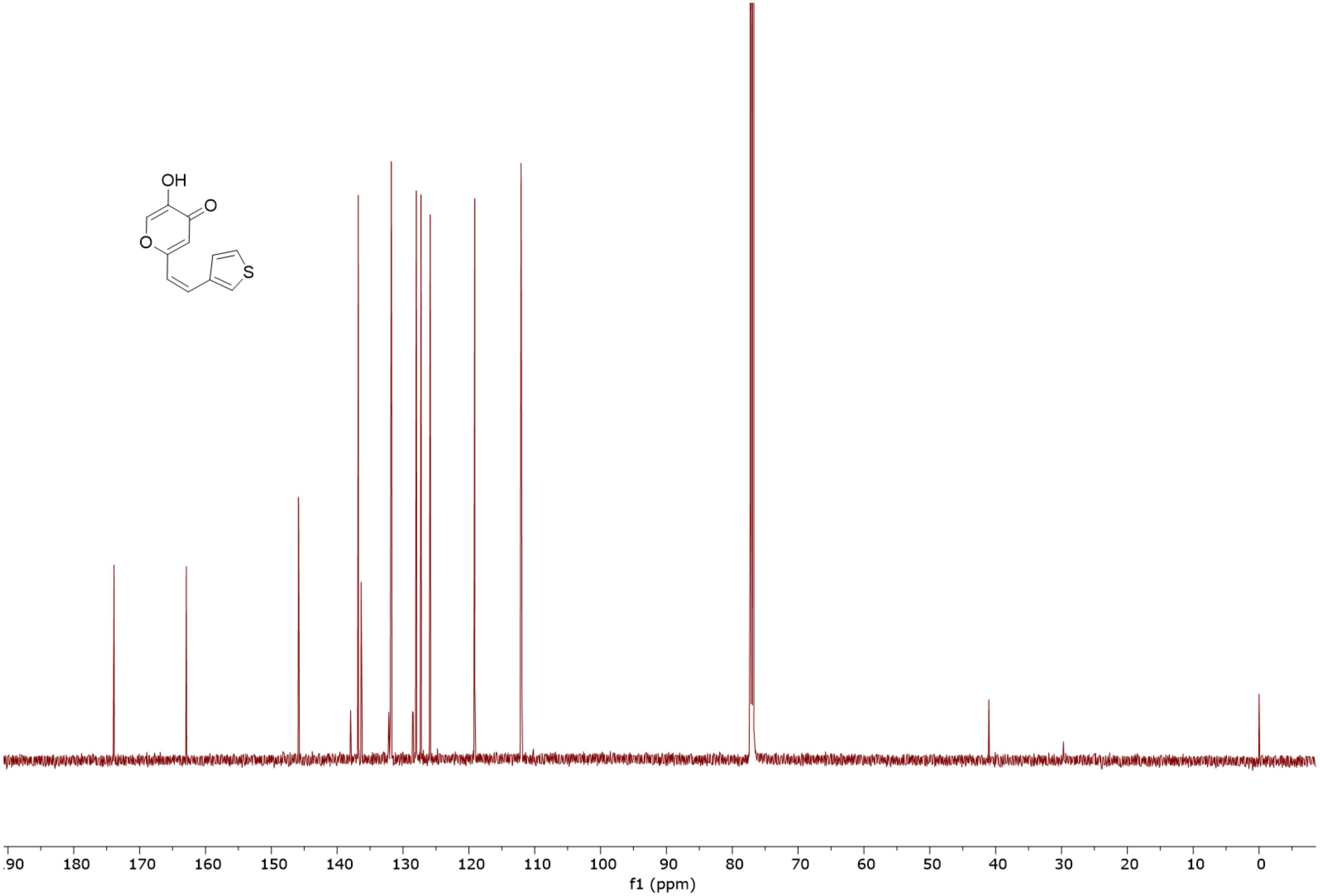

Figure S23. ^{13}C NMR spectrum ($CDCl_3$) of (*Z*)-5-hydroxy-2-(2-(thiophen-3-yl)vinyl)-4*H*-pyran-4-one (*cis*-**6**).

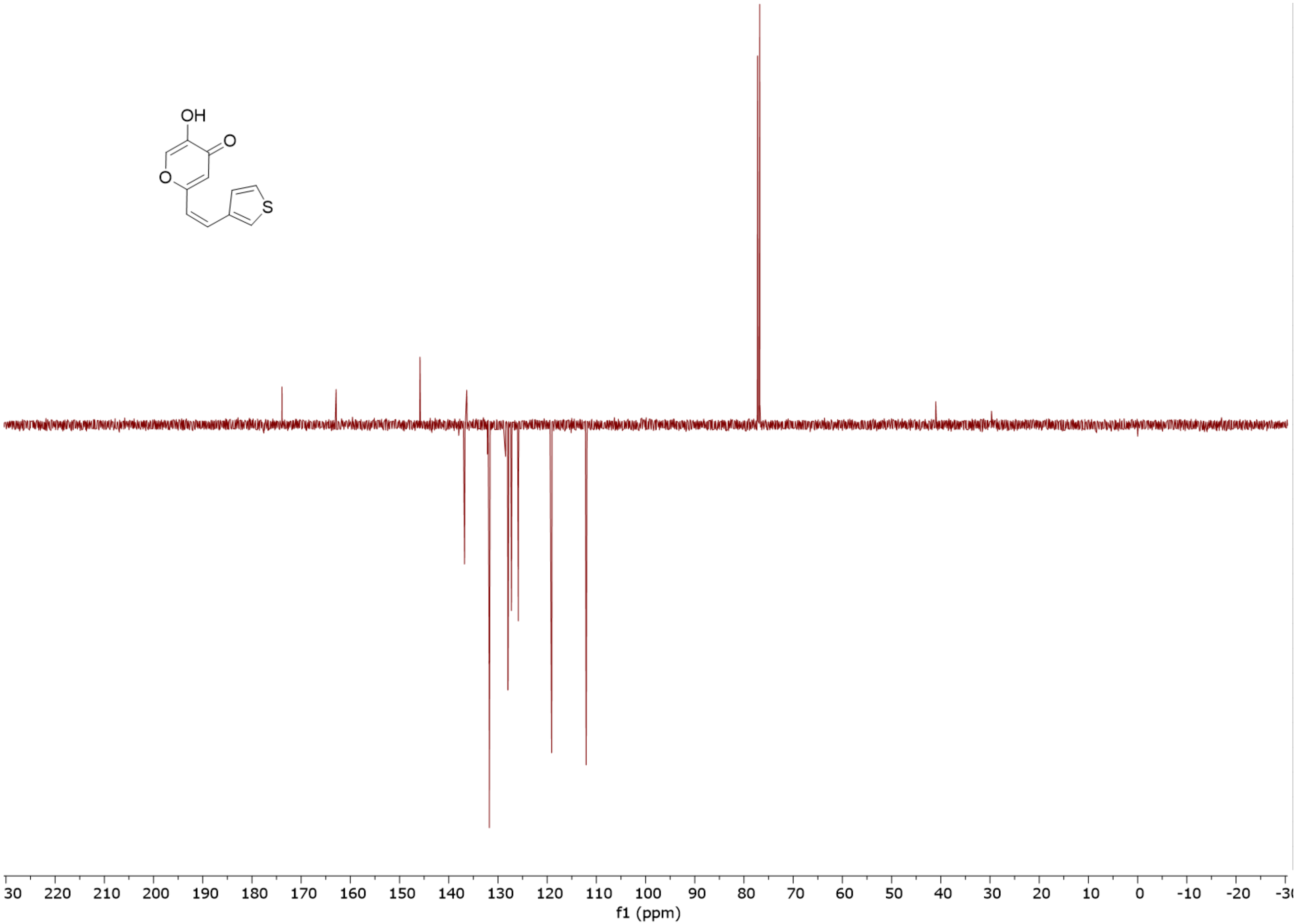

Figure S24. ^{13}C APT NMR spectrum ($CDCl_3$) of (*Z*)-5-hydroxy-2-(2-(thiophen-3-yl)vinyl)-4*H*-pyran-4-one (*cis*-**6**).

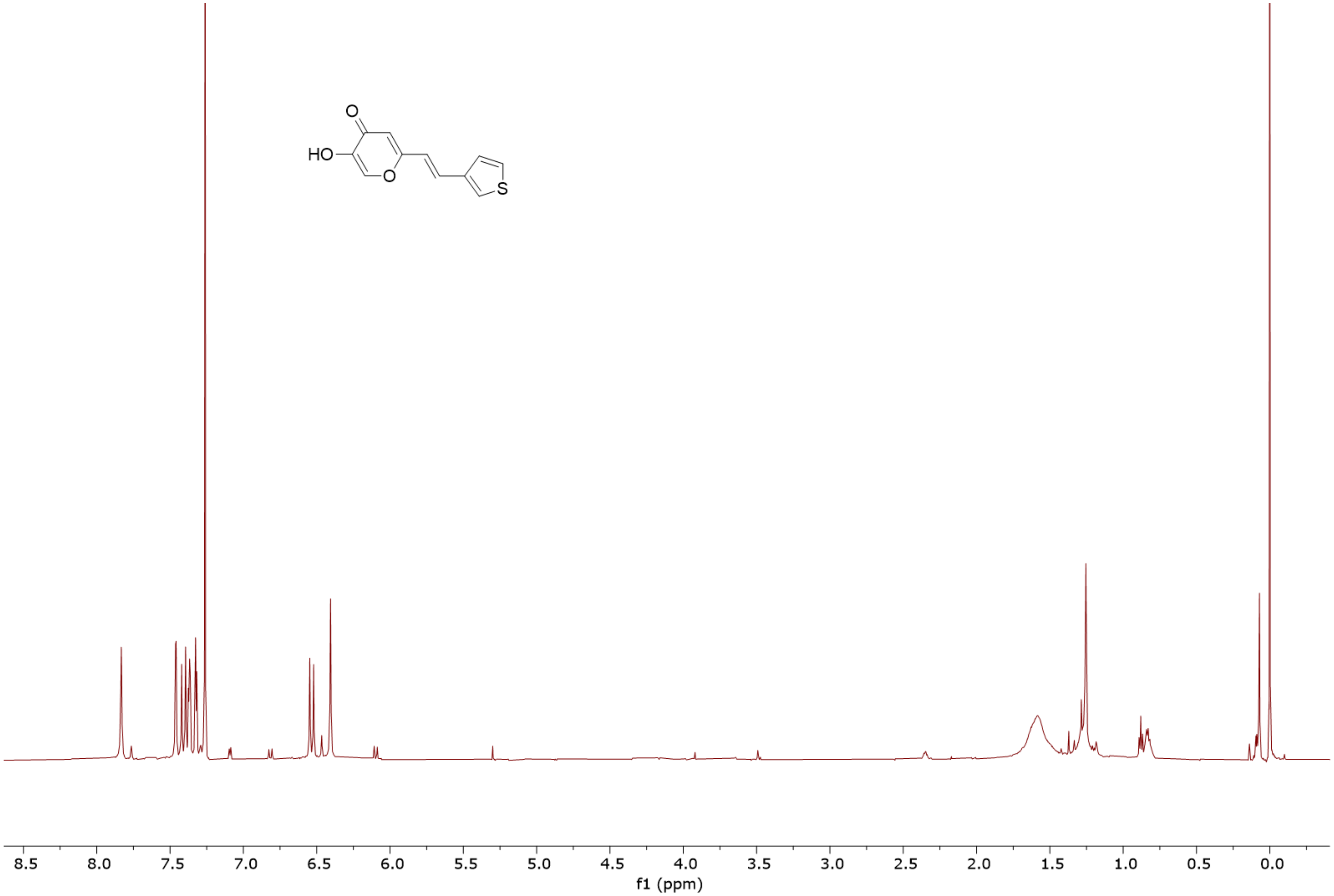

Figure S25. ^{1}H NMR spectrum ($CDCl_3$) of (*E*)-5-hydroxy-2-(2-(thiophen-3-yl)vinyl)-4*H*-pyran-4-one (*trans*-**6**).

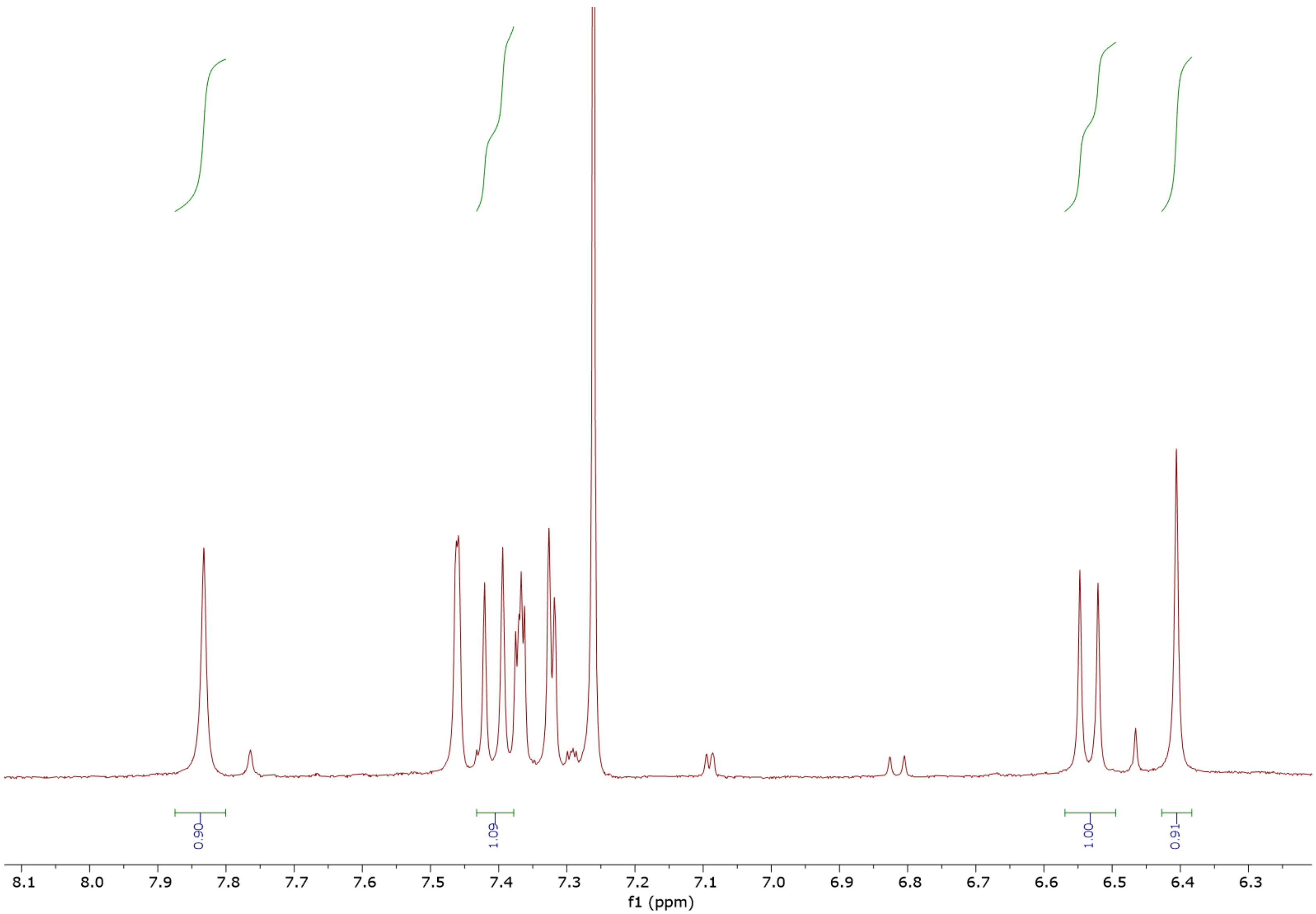

Figure S26. Part of the ^{1}H NMR spectrum ($CDCl_3$) of (*E*)-5-hydroxy-2-(2-(thiophen-3-yl)vinyl)-4*H*-pyran-4-one (*trans*-**6**).

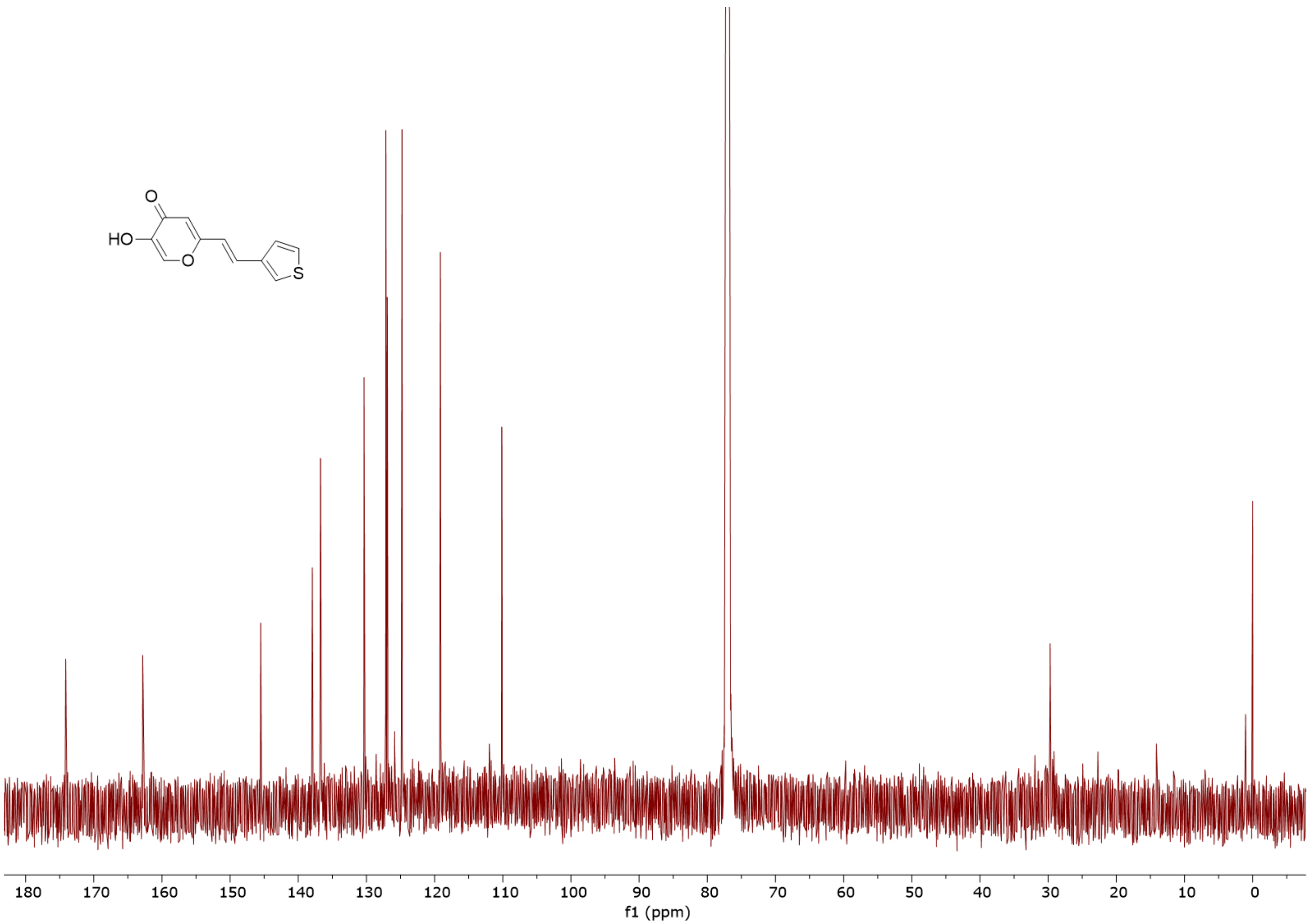

Figure S27. ^{13}C NMR spectrum ($CDCl_3$) of (*E*)-5-hydroxy-2-(2-(thiophen-3-yl)vinyl)-4*H*-pyran-4-one (*trans*-**6**).

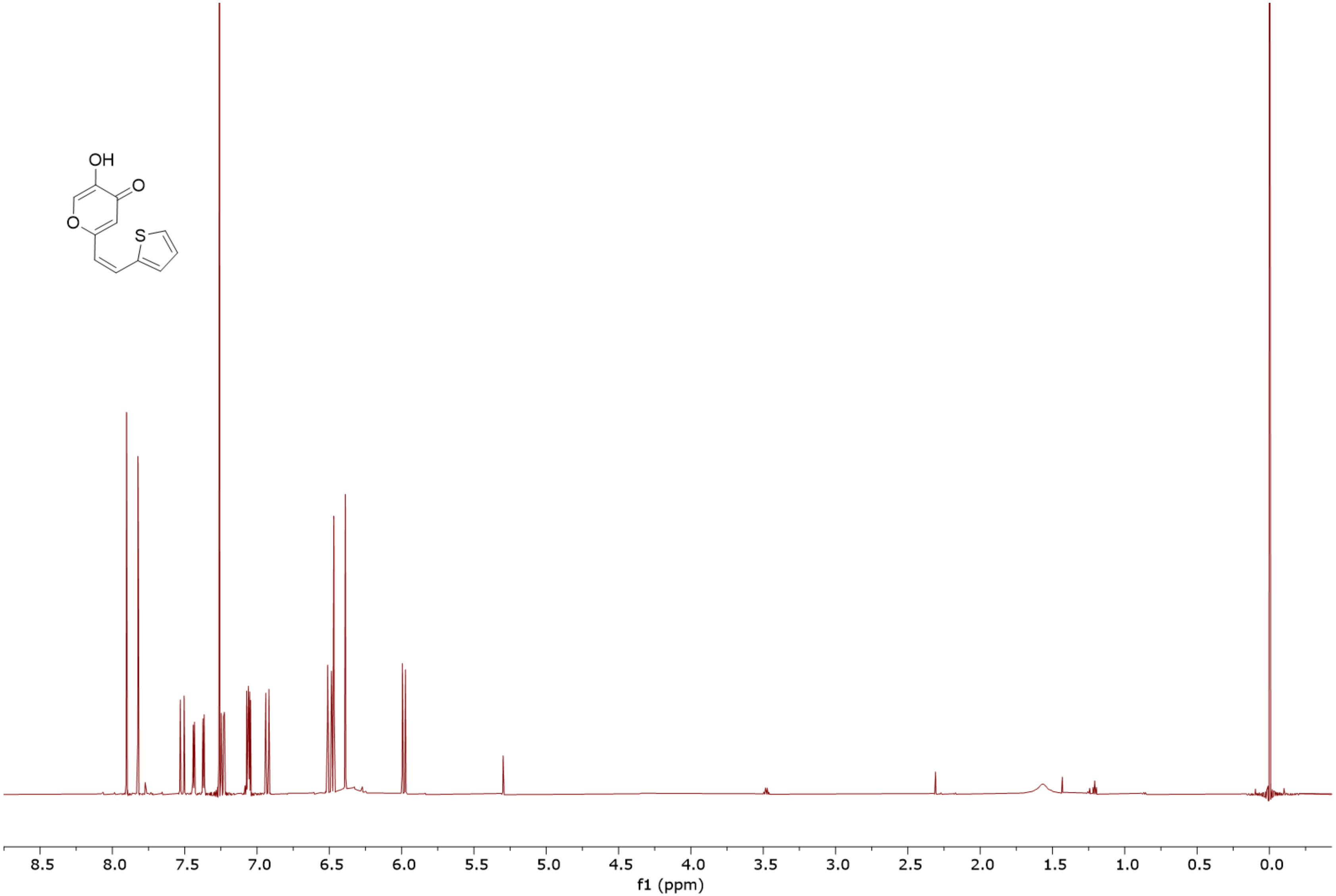

Figure S28. ^{1}H NMR spectrum ($CDCl_3$) of (*Z*)-5-hydroxy-2-(2-(thiophen-2-yl)vinyl)-4*H*-pyran-4-one (*cis*-**7**, with *trans*-**7**).

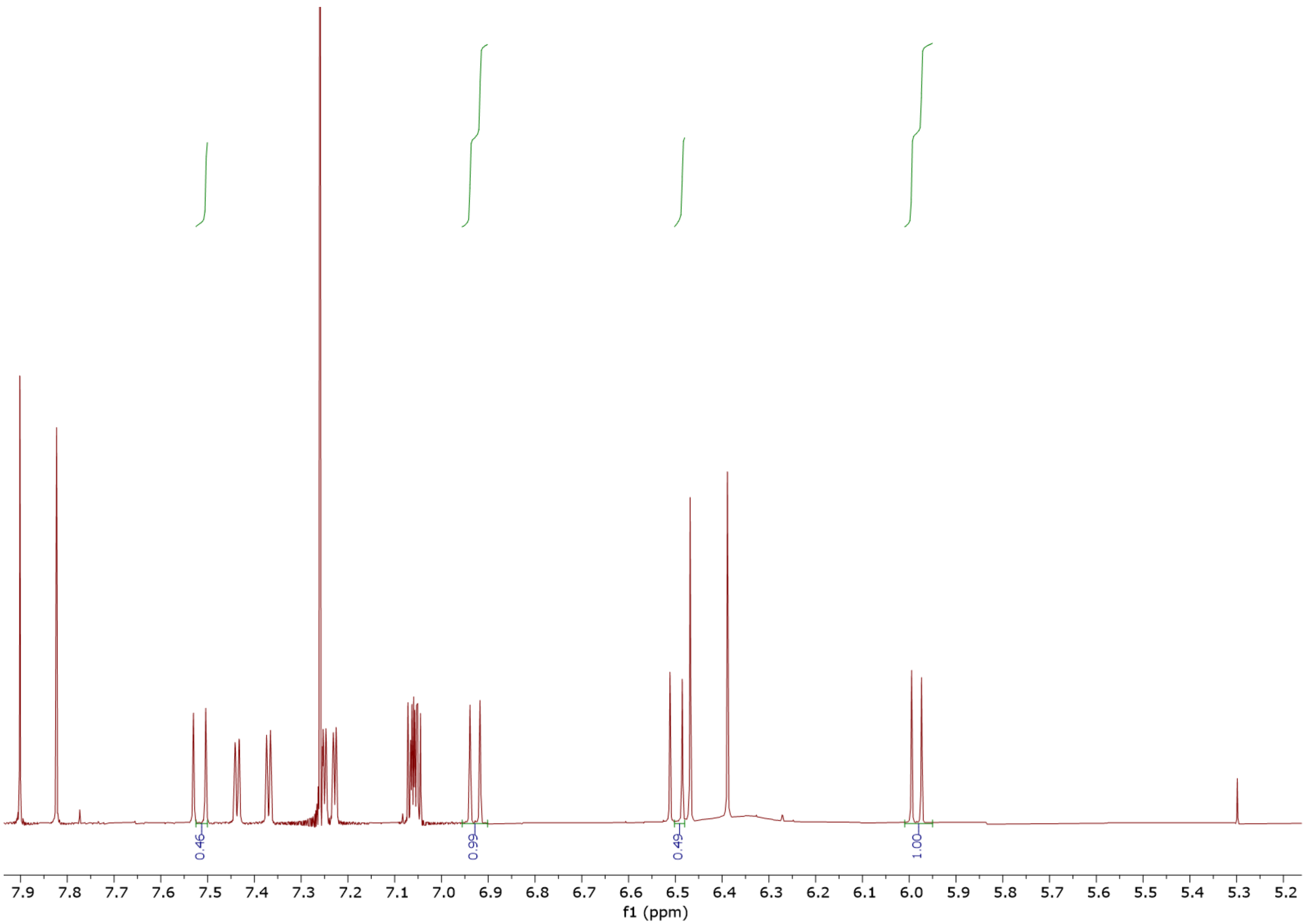

Figure S29. Part of the ^{1}H NMR spectrum ($CDCl_3$) of (*Z*)-5-hydroxy-2-(2-(thiophen-2-yl)vinyl)-4*H*-pyran-4-one (*cis*-**7**, with *trans*-**7**).

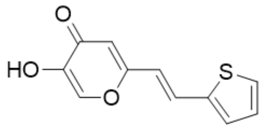

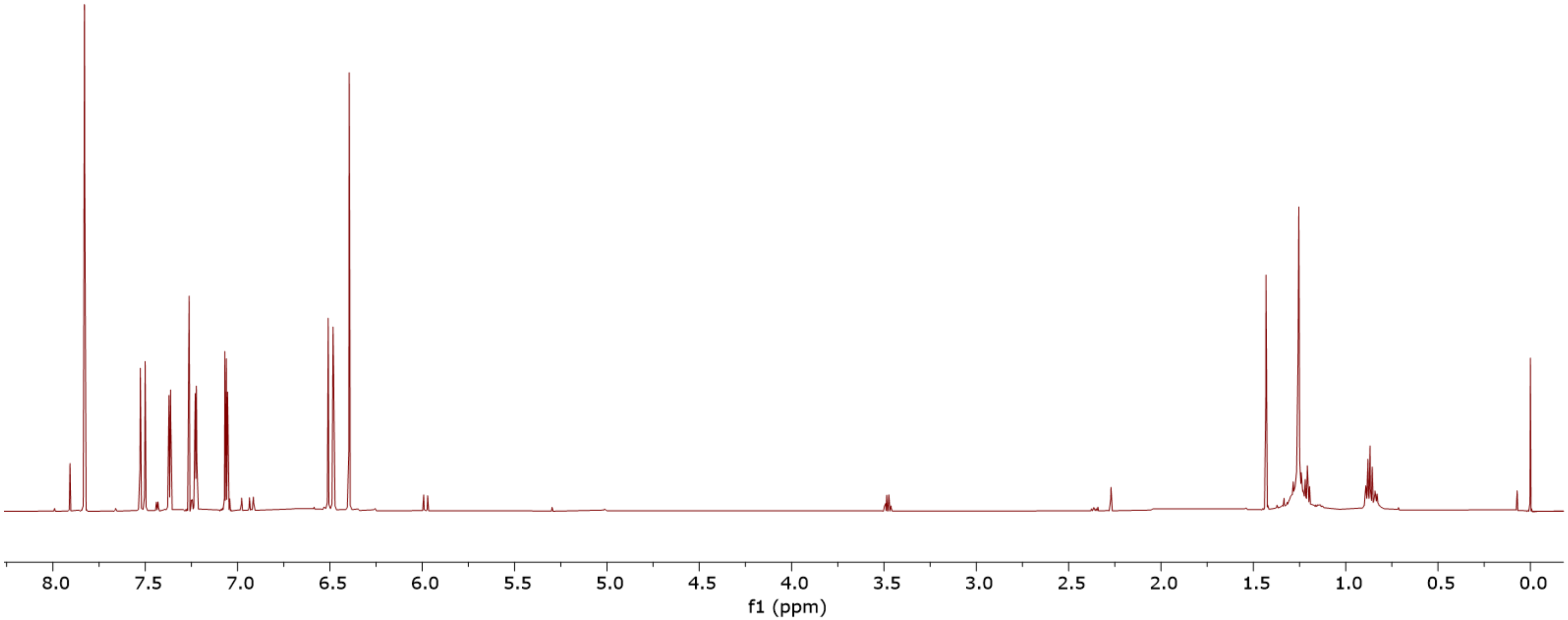

Figure S30. ^{1}H NMR spectrum ($CDCl_3$) of (*E*)-5-hydroxy-2-(2-(thiophen-2-yl)vinyl)-4*H*-pyran-4-one (*trans*-**7**).

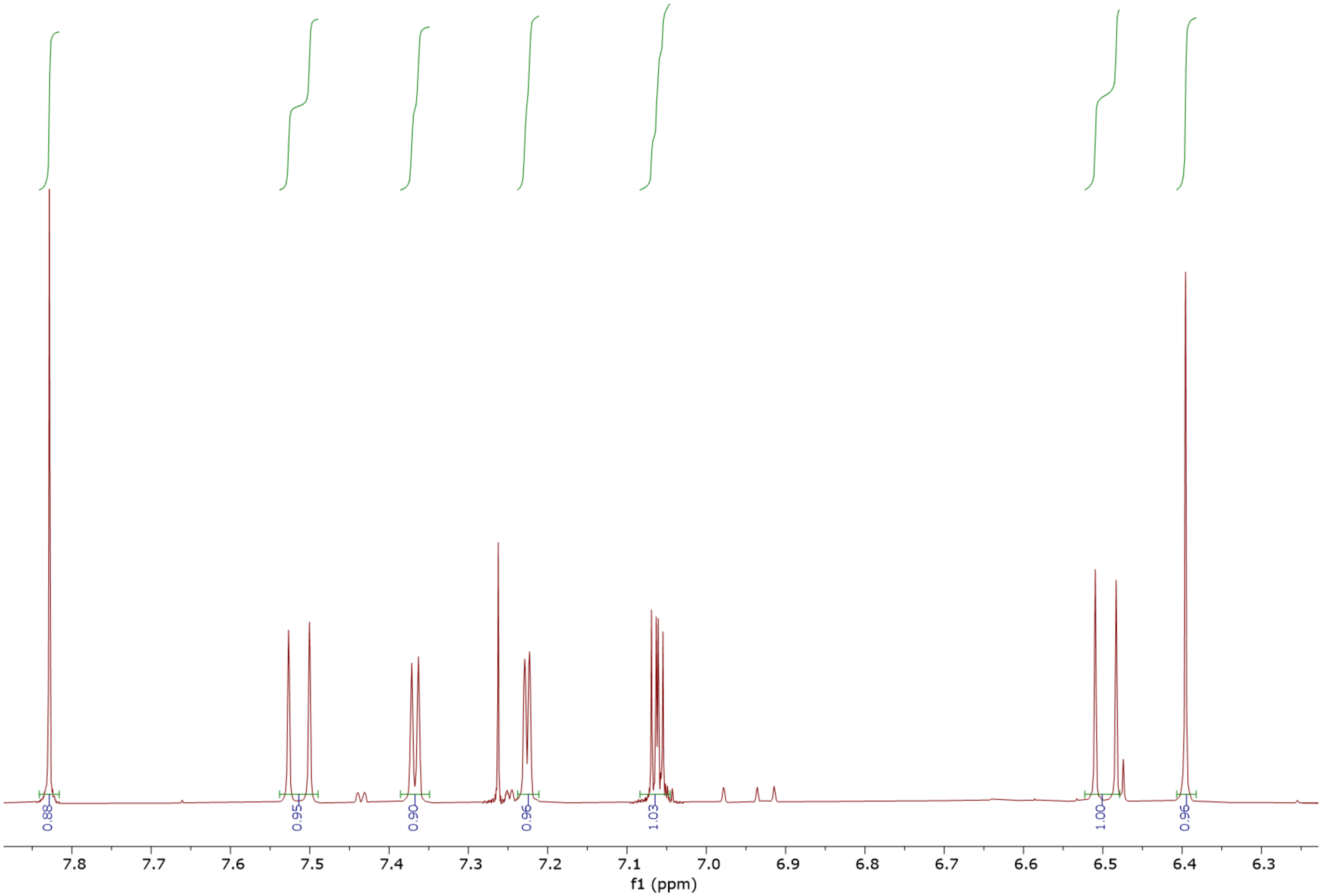

Figure S31. Part of the ^{1}H NMR spectrum ($CDCl_3$) of (*E*)-5-hydroxy-2-(2-(thiophen-2-yl)vinyl)-4*H*-pyran-4-one (*trans*-**7**).

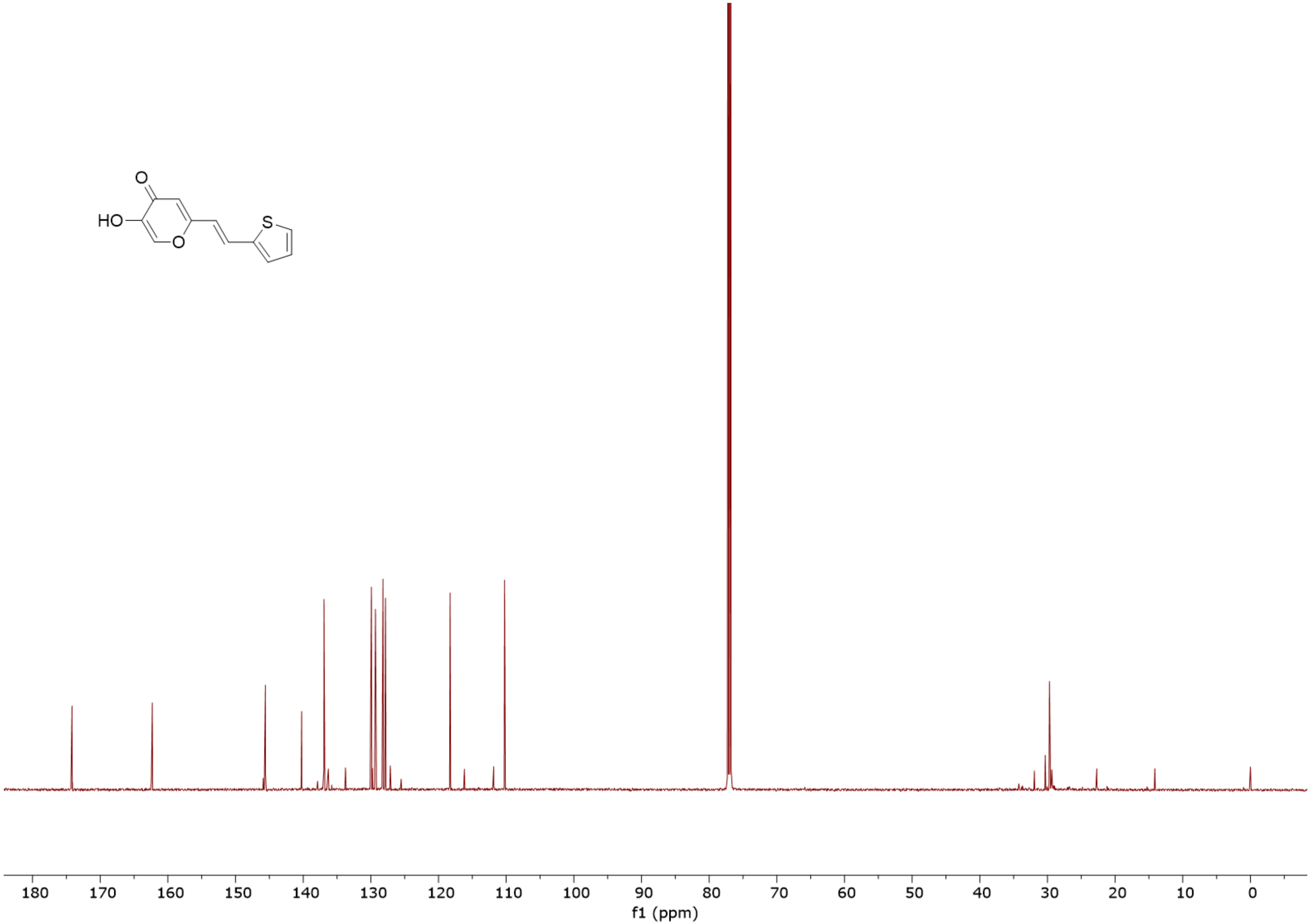

Figure S32. ^{13}C NMR spectrum ($CDCl_3$) of (*E*)-5-hydroxy-2-(2-(thiophen-2-yl)vinyl)-4*H*-pyran-4-one (*trans*-**7**).

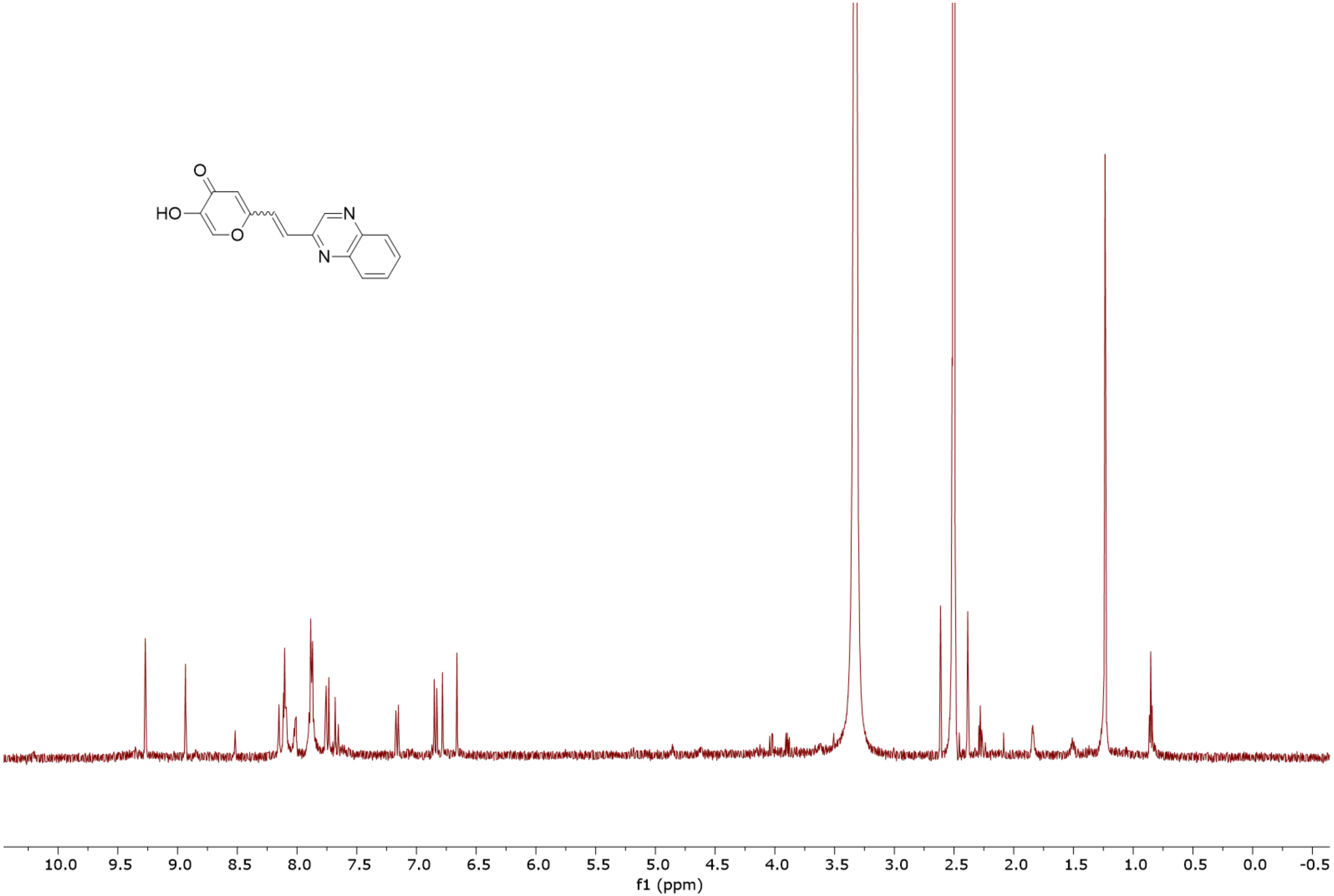

Figure S33. ^{1}H NMR spectrum (DMSO-d6) of 5-hydroxy-2-(2-(quinoxalin-2-yl)vinyl)-4*H*-pyran-4-one **8** (mixture of isomers).

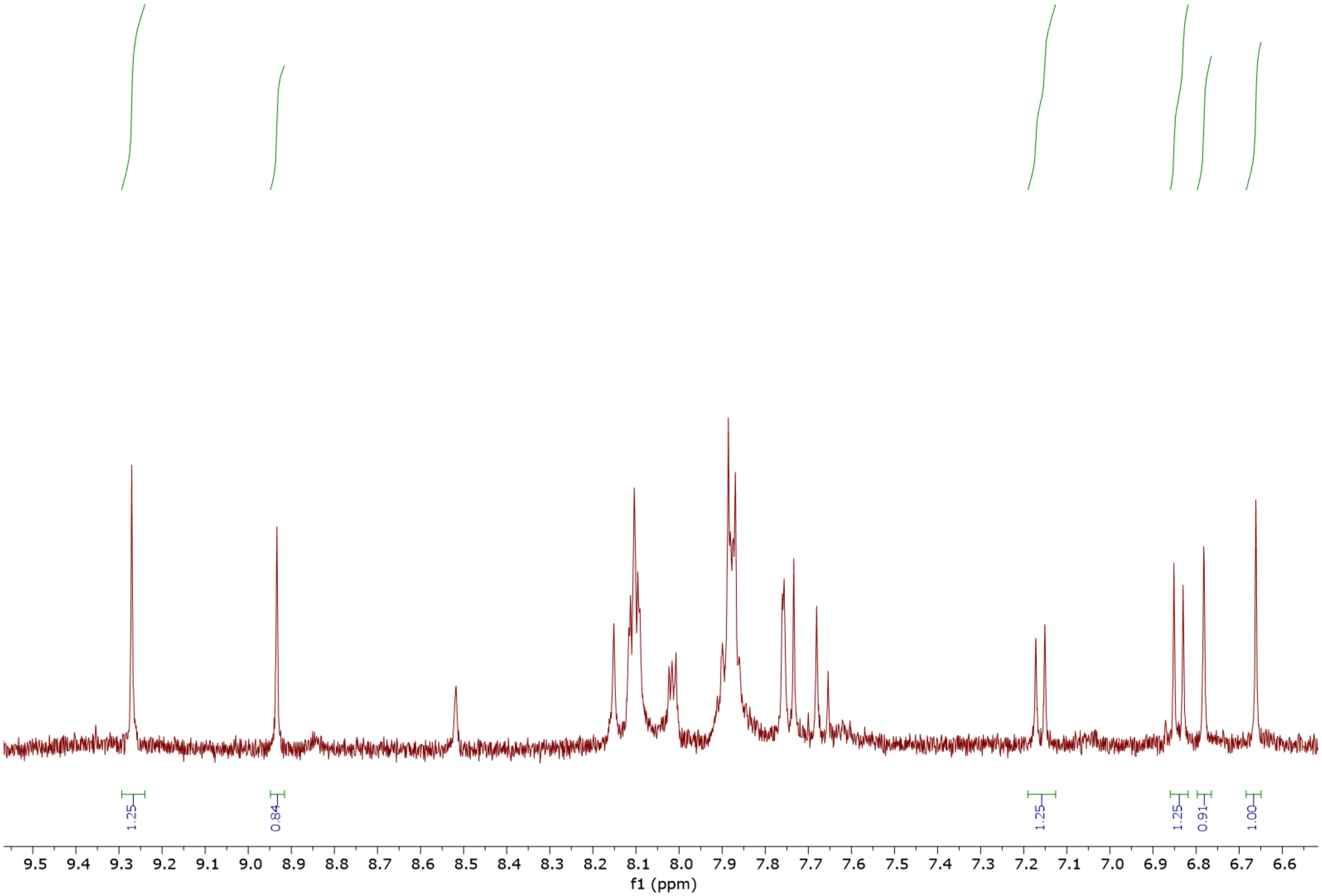

Figure S34. Part of the ^{1}H NMR spectrum (DMSO-d6) of 5-hydroxy-2-(2-(quinoxalin-2-yl)vinyl)-4*H*-pyran-4-one (mixture of isomers-**8**).

2. UV spectra of pure compounds

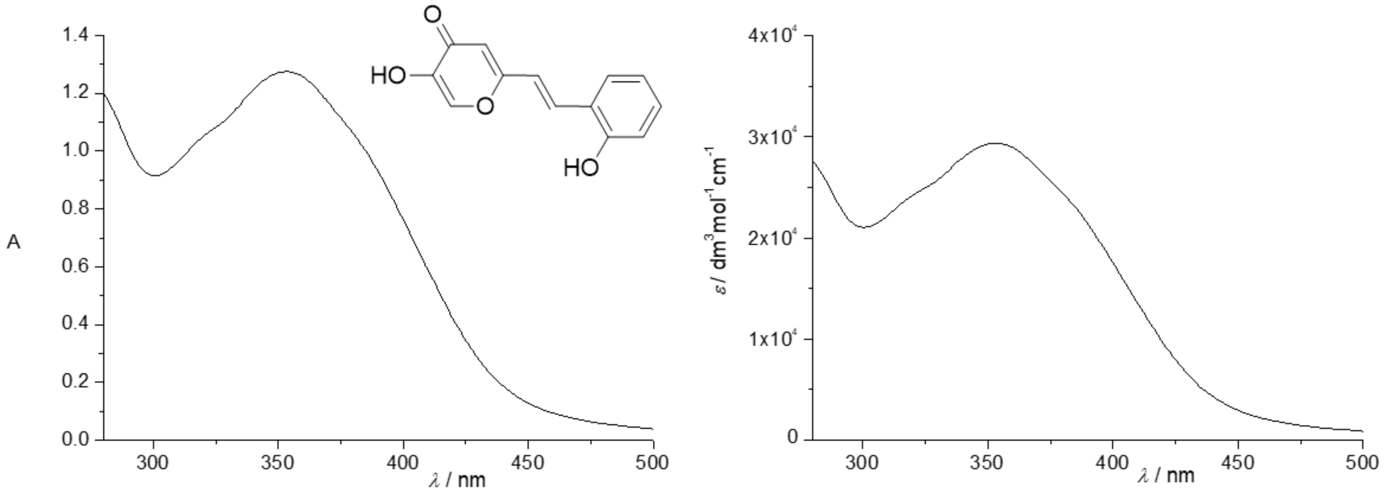

Figure S35. UV spectra (ACN) of (*E*)-5-hydroxy-2-(2-hydroxystyryl)-4*H*-pyran-4-one (*trans*-**1**).

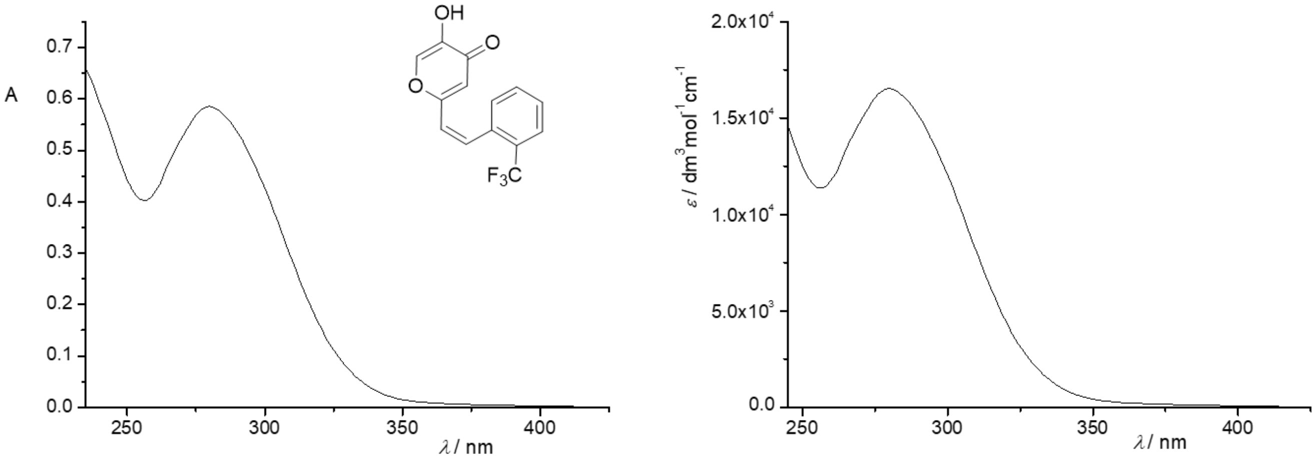

Figure S36. UV spectra (ACN) of (*Z*)-5-hydroxy-2-(2-(trifluoromethyl)styryl)-4*H*-pyran-4-one (*cis*-**2**).

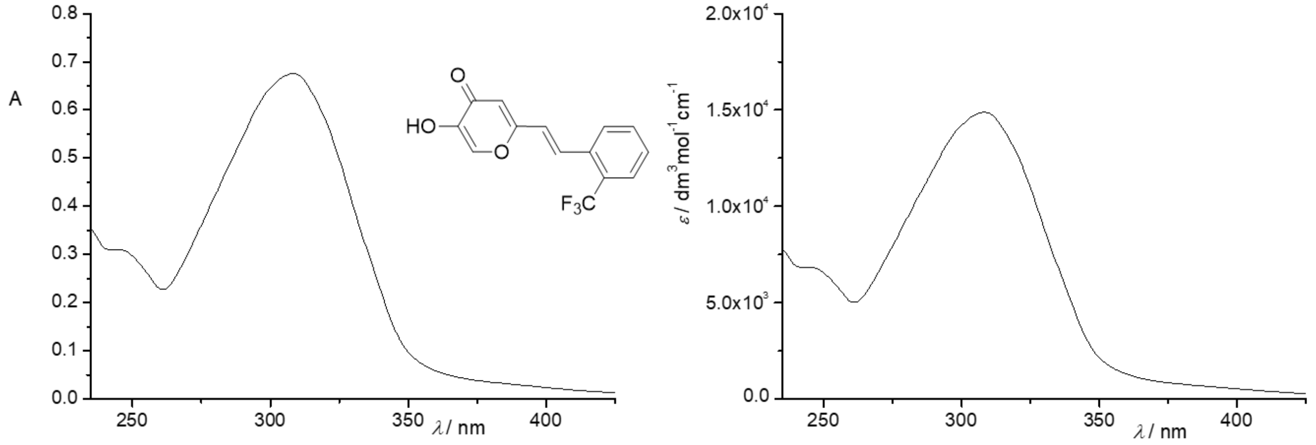

Figure S37. UV spectra (ACN) of (*E*)-5-hydroxy-2-(2-(trifluoromethyl)styryl)-4*H*-pyran-4-one (*trans*-**2**).

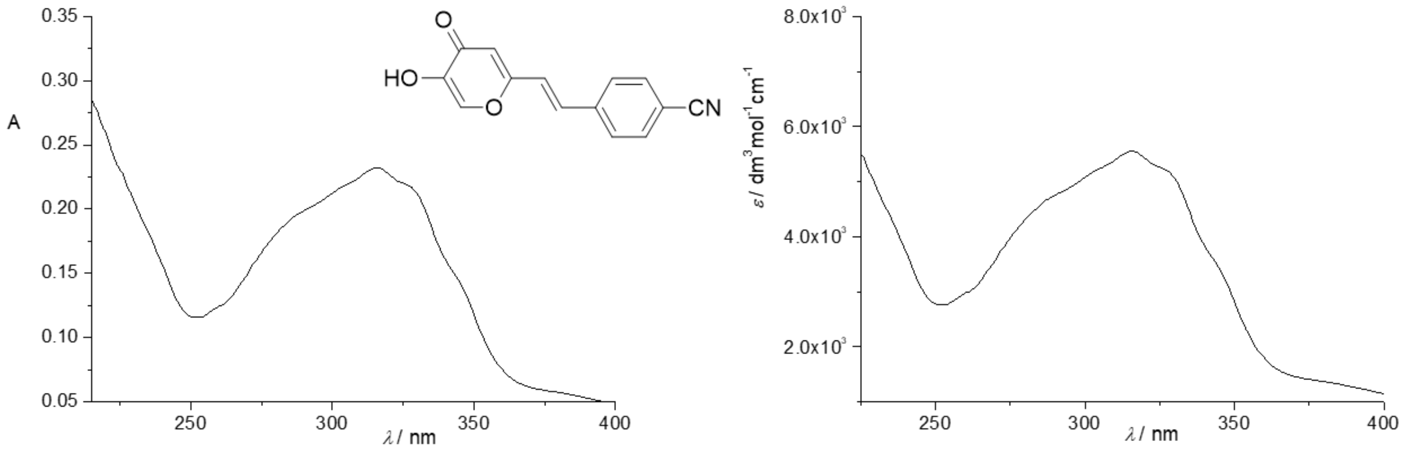

Figure S38. UV spectra (ACN) of 4-(2-(5-hydroxy-4-oxo-4*H*-pyran-2-yl)vinyl)benzonitrile (mixture of isomers-**3**).

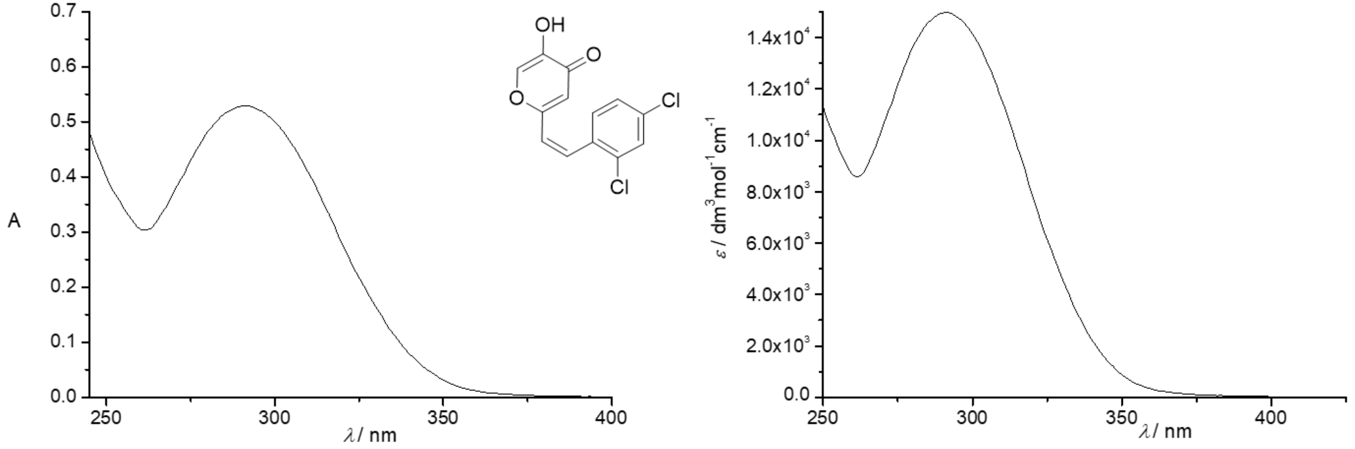

Figure S39. UV spectra (ACN) of (*Z*)-2-(2,4-dichlorostyryl)-5-hydroxy-4*H*-pyran-4-one (*cis*-**4**).

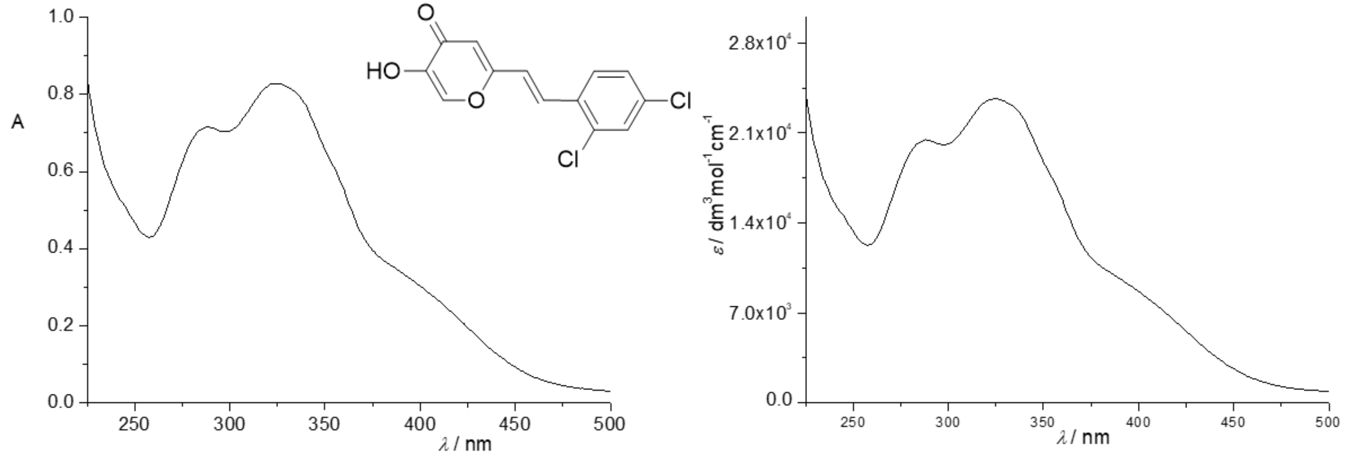

Figure S40. UV spectra (ACN) of (*E*)-2-(2,4-dichlorostyryl)-5-hydroxy-4*H*-pyran-4-one (*trans*-**4**).

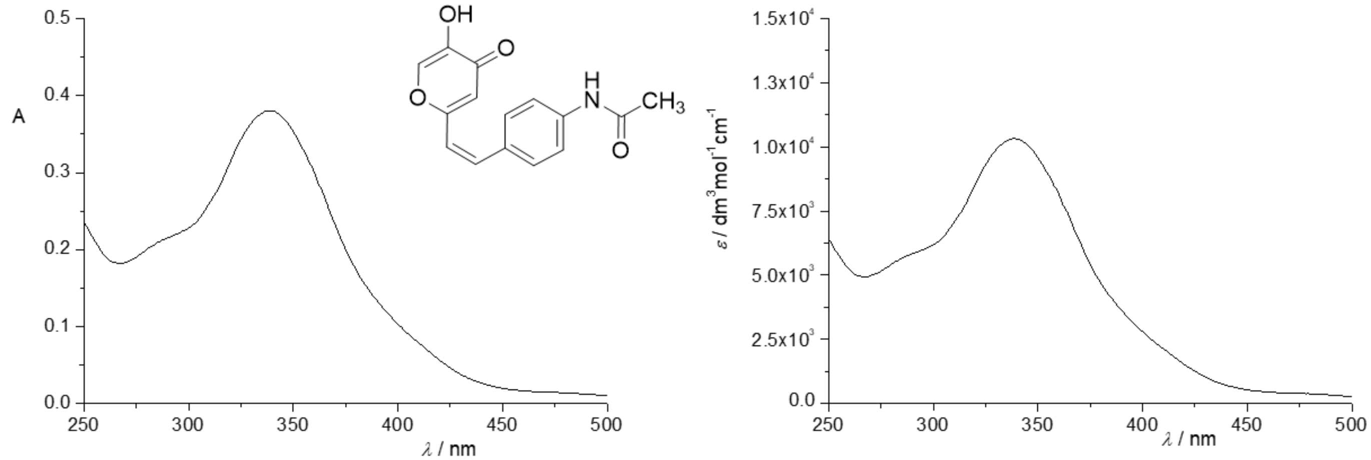

Figure S41. UV spectra (ACN) of (*Z*)-*N*-(4-(2-(5-hydroxy-4-oxo-4*H*-pyran-2-yl)vinyl)phenyl)acetamide (*cis*-**5**).

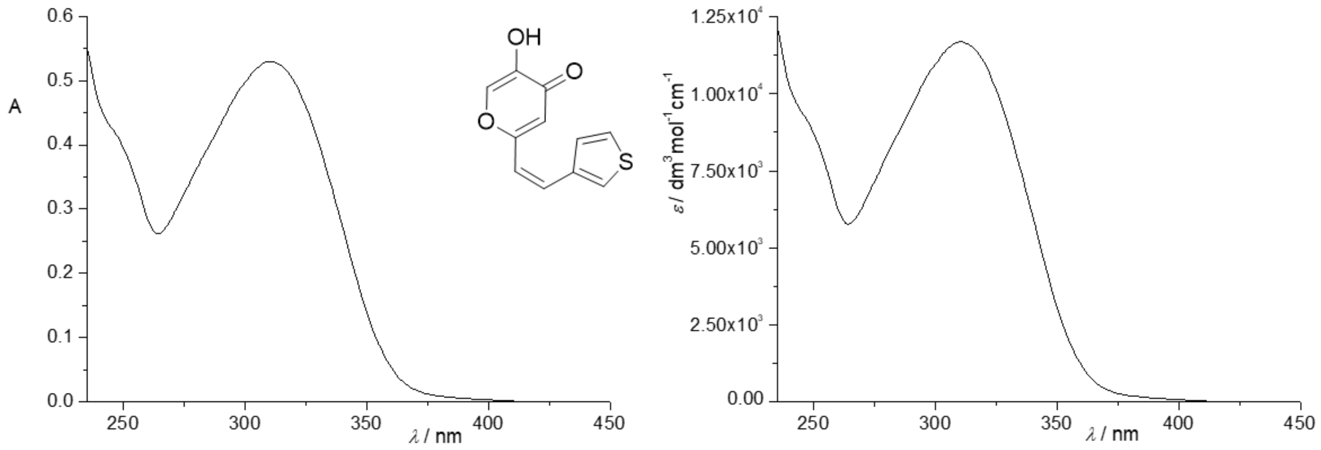

Figure S42. UV spectra (ACN) of (*Z*)-5-hydroxy-2-(2-(thiophen-3-yl)vinyl)-4*H*-pyran-4-one (*cis*-**6**).

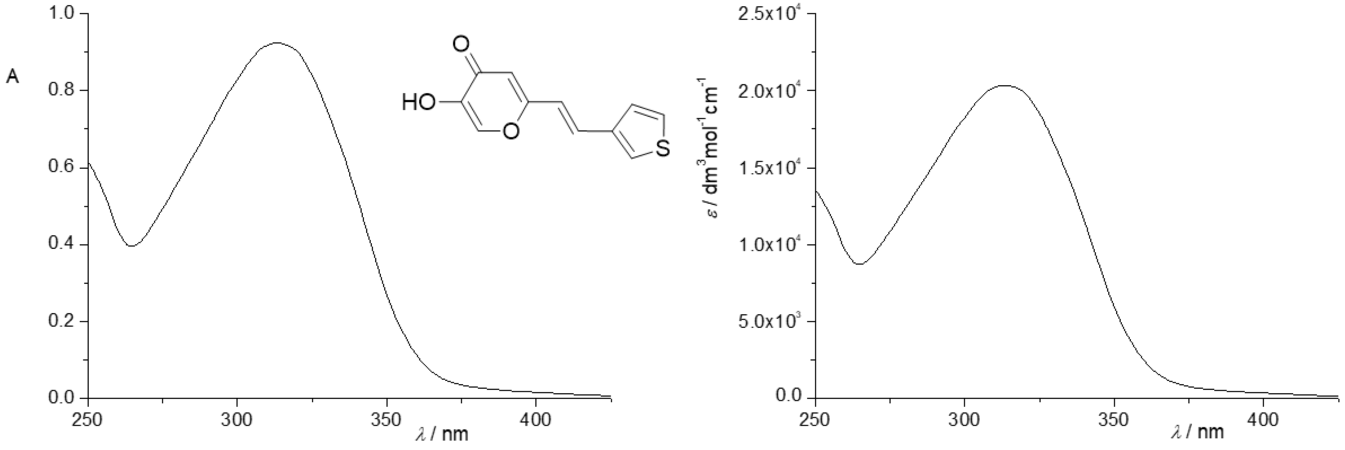

Figure S43. UV spectra (ACN) of (*E*)-5-hydroxy-2-(2-(thiophen-3-yl)vinyl)-4*H*-pyran-4-one (*trans*-**6**).

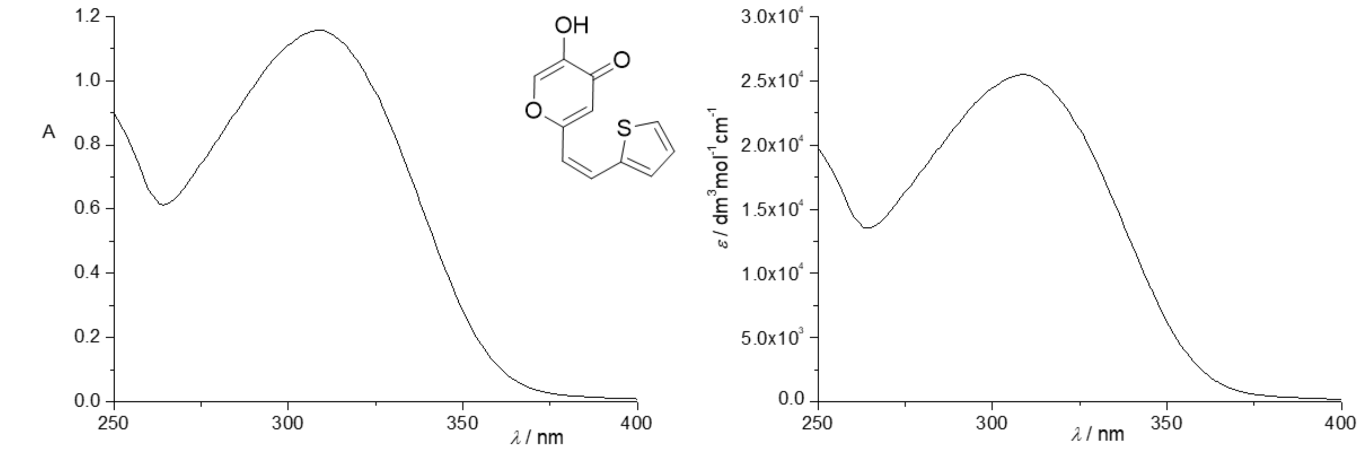

Figure S44. UV spectra (ACN) of (*Z*)-5-hydroxy-2-(2-(thiophen-2-yl)vinyl)-4*H*-pyran-4-one (*cis*-**7**).

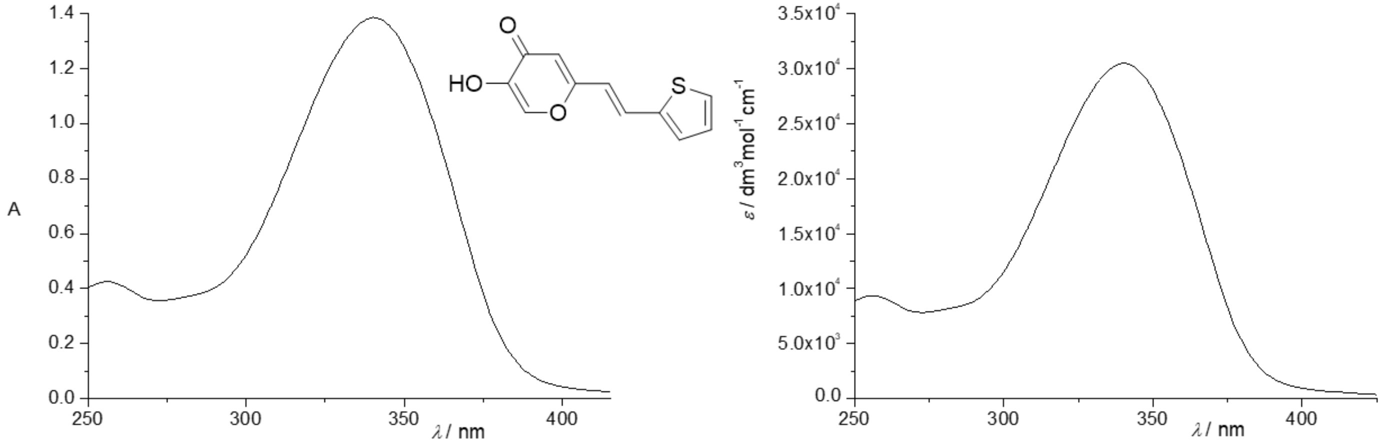

Figure S45. UV spectra (ACN) of (*E*)-5-hydroxy-2-(2-(thiophen-2-yl)vinyl)-4*H*-pyran-4-one (*trans*-**7**).

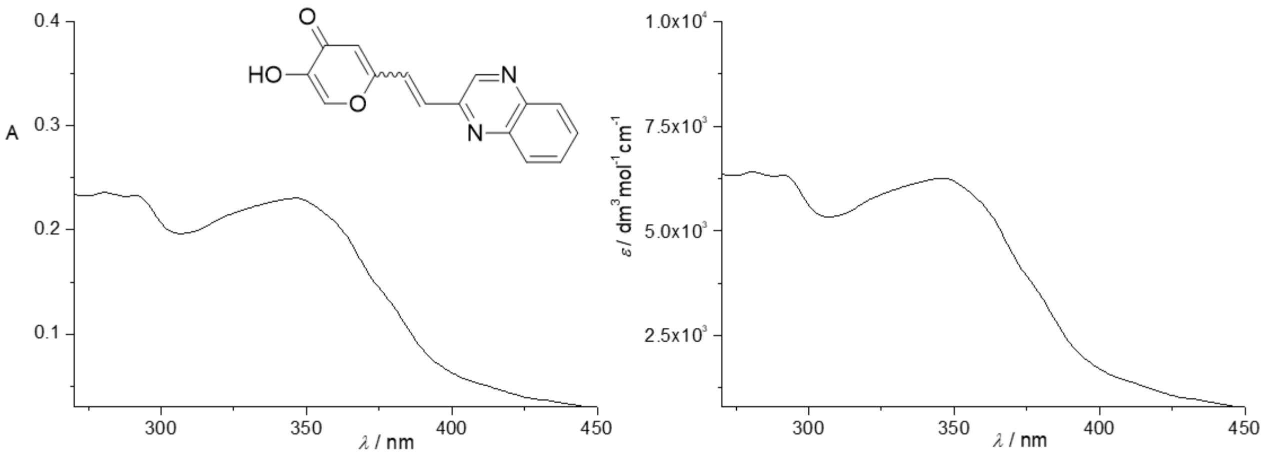

Figure S46. UV spectra (ACN) of 5-hydroxy-2-(2-(quinoxalin-2-yl)vinyl)-4*H*-pyran-4-one (mixture of isomers-**8**).

3. IR spectra of pure compounds

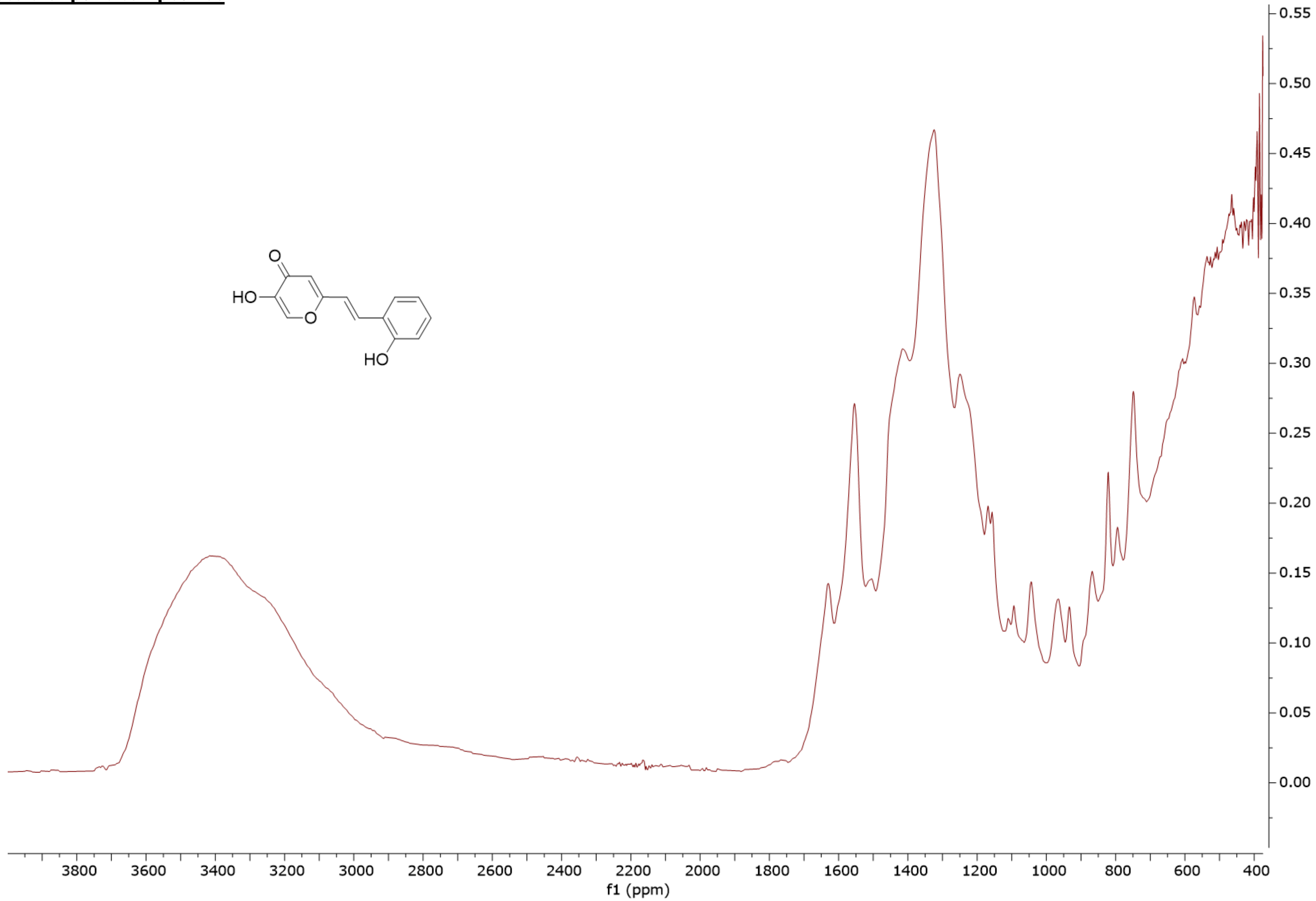

Figure S47. IR ART spectrum of (*E*)-5-hydroxy-2-(2-hydroxystyryl)-4*H*-pyran-4-one (*trans*-**1**).

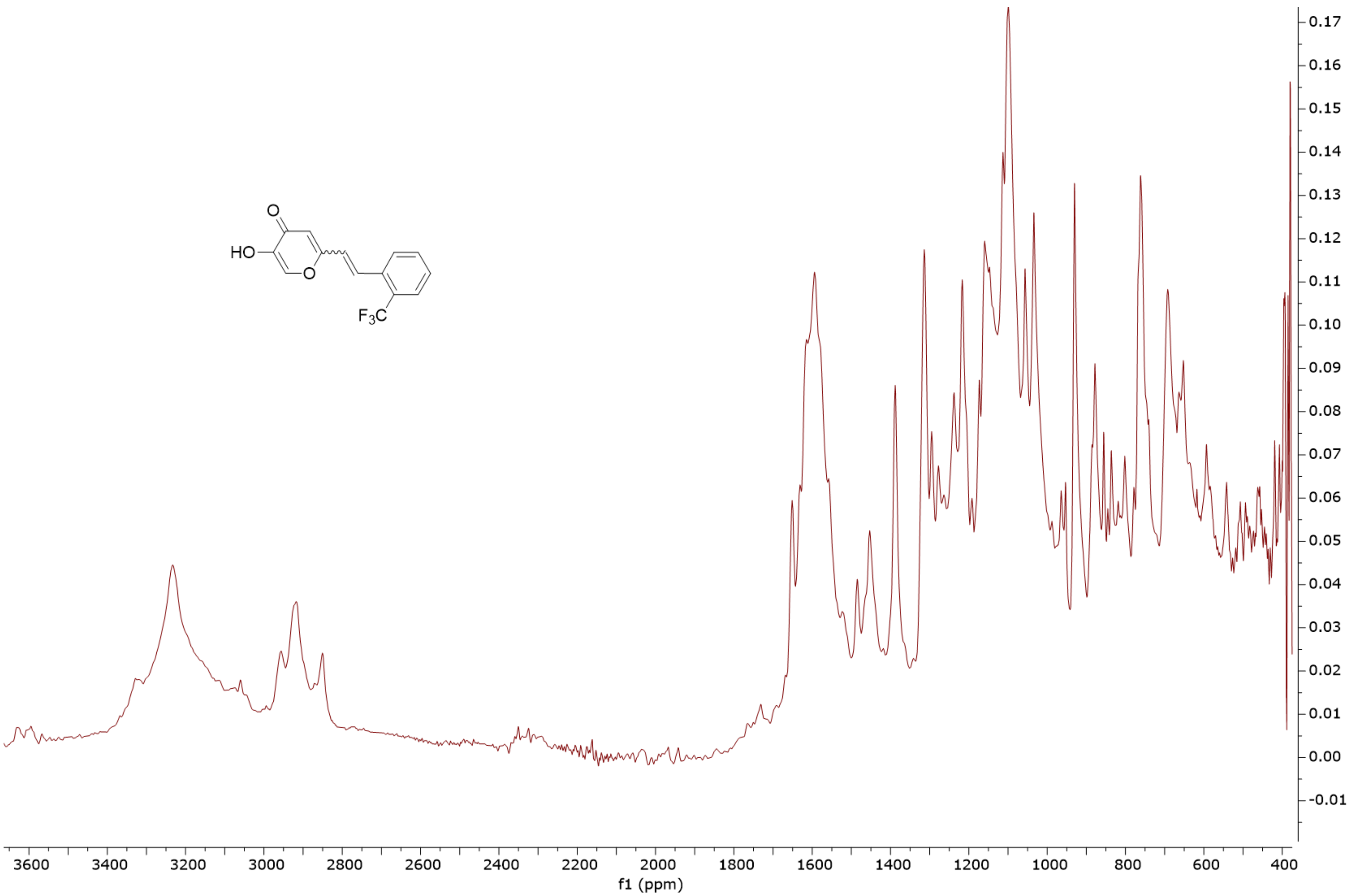

Figure S48. IR ATR spectrum of 5-hydroxy-2-(2-(trifluoromethyl)styryl)-4*H*-pyran-4-one (**2**).

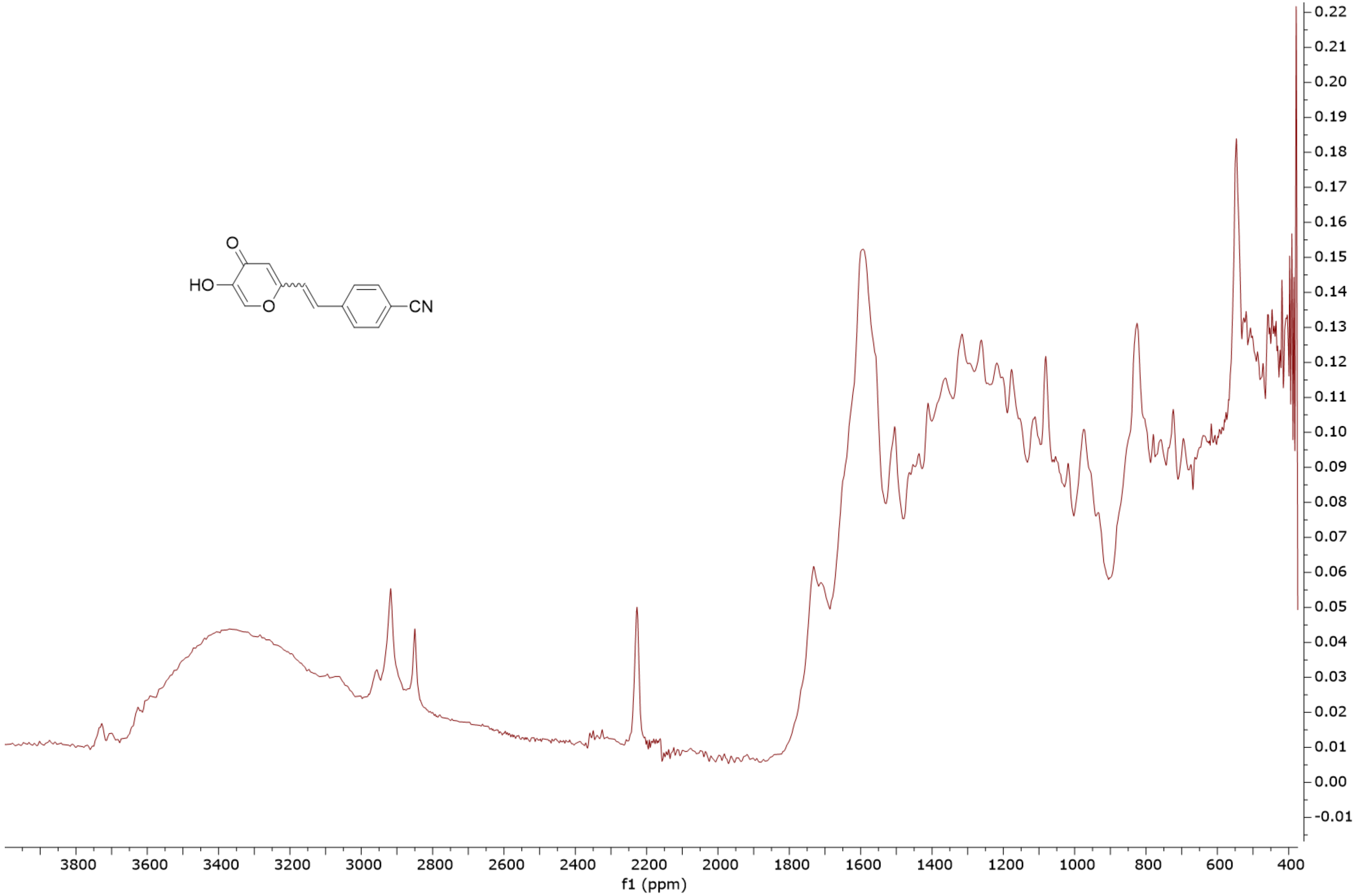

Figure S49. IR ATR spectrum of 4-(2-(5-hydroxy-4-oxo-4*H*-pyran-2-yl)vinyl)benzonitrile (**3**).

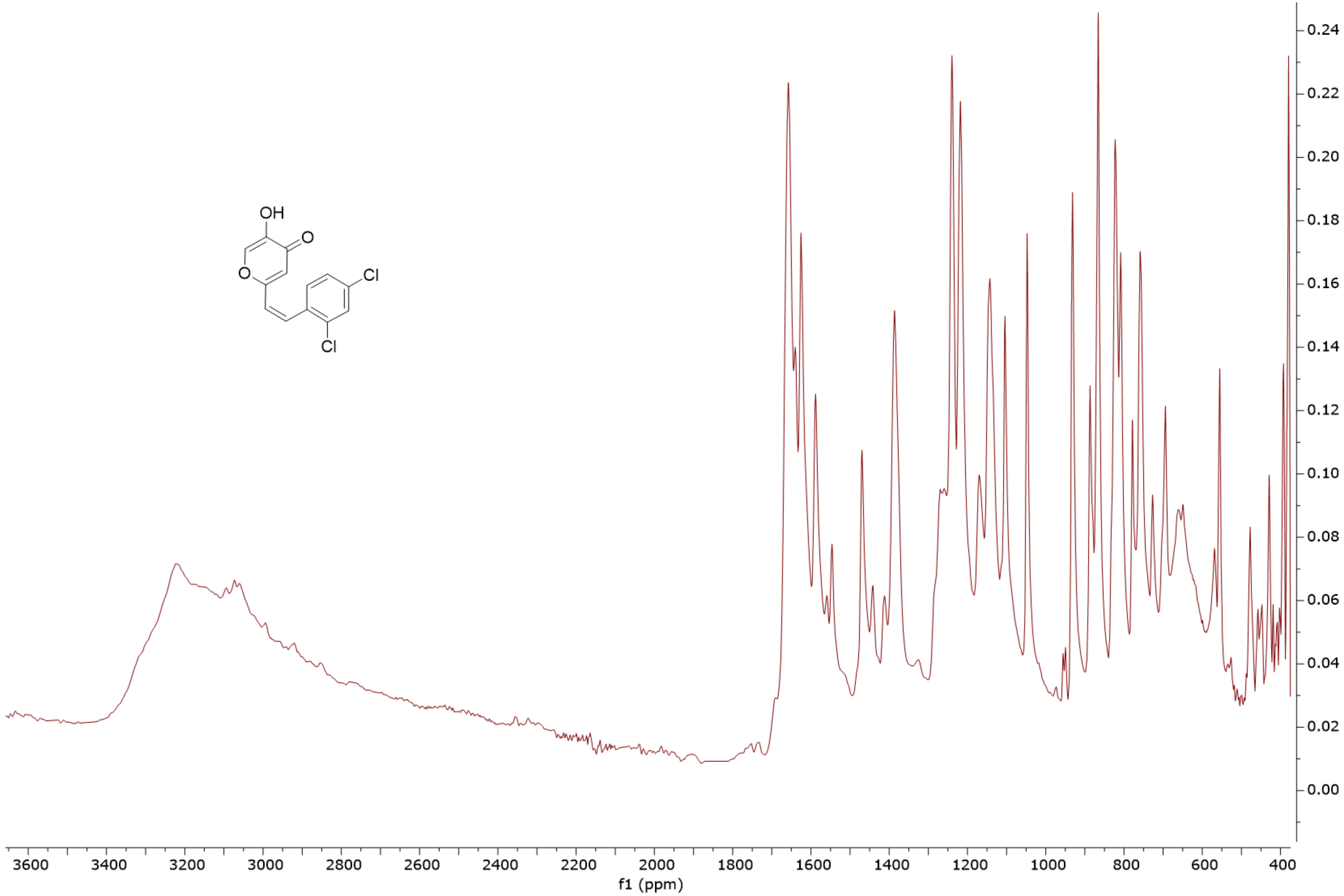

Figure S50. IR ATR spectrum of (*Z*)-2-(2,4-dichlorostyryl)-5-hydroxy-4*H*-pyran-4-one (*cis*-**4**).

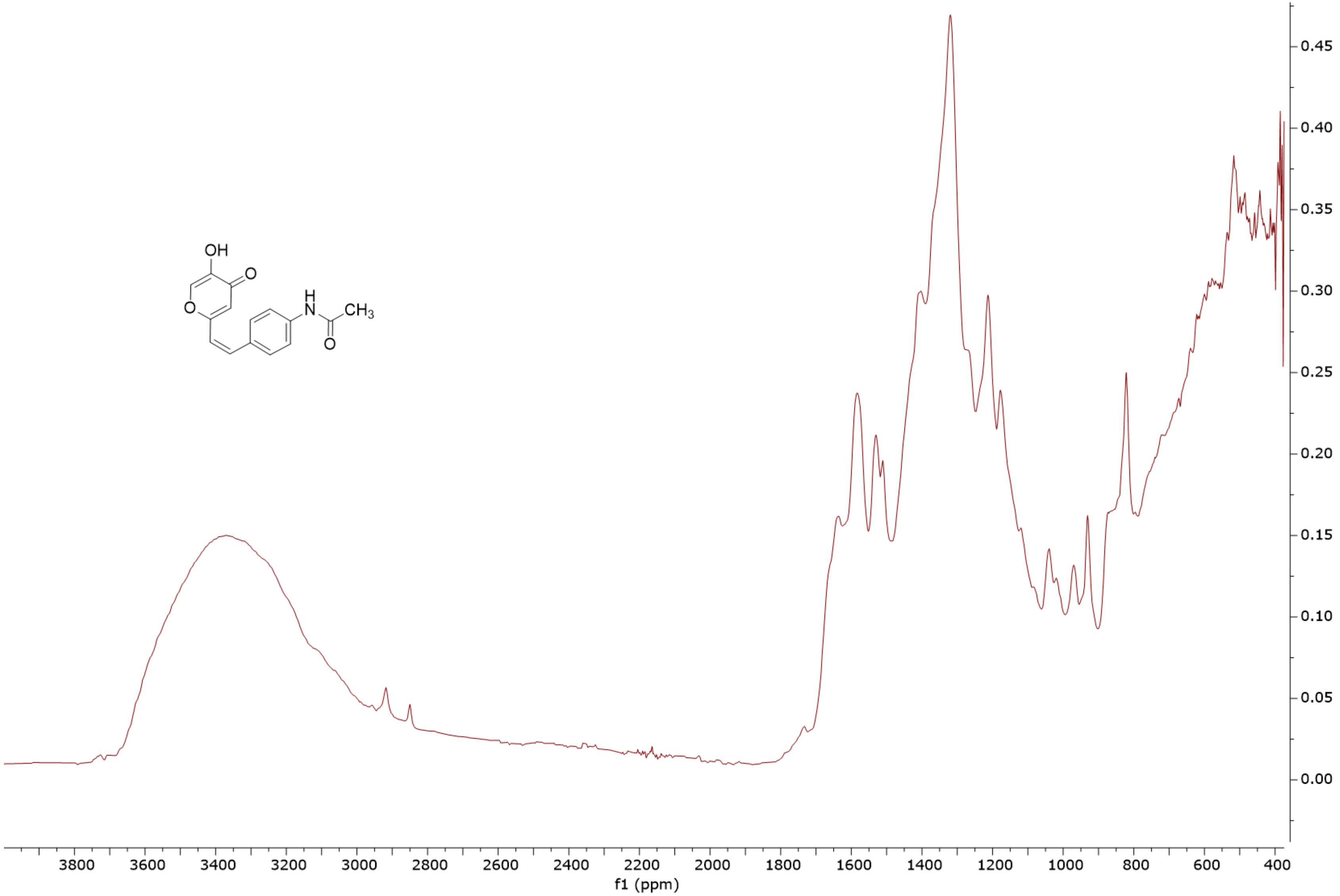

Figure S51. IR ATR spectrum of (*Z*)-*N*-(4-(2-(5-hydroxy-4-oxo-4*H*-pyran-2-yl)vinyl)phenyl)acetamide (*cis*-**5**).

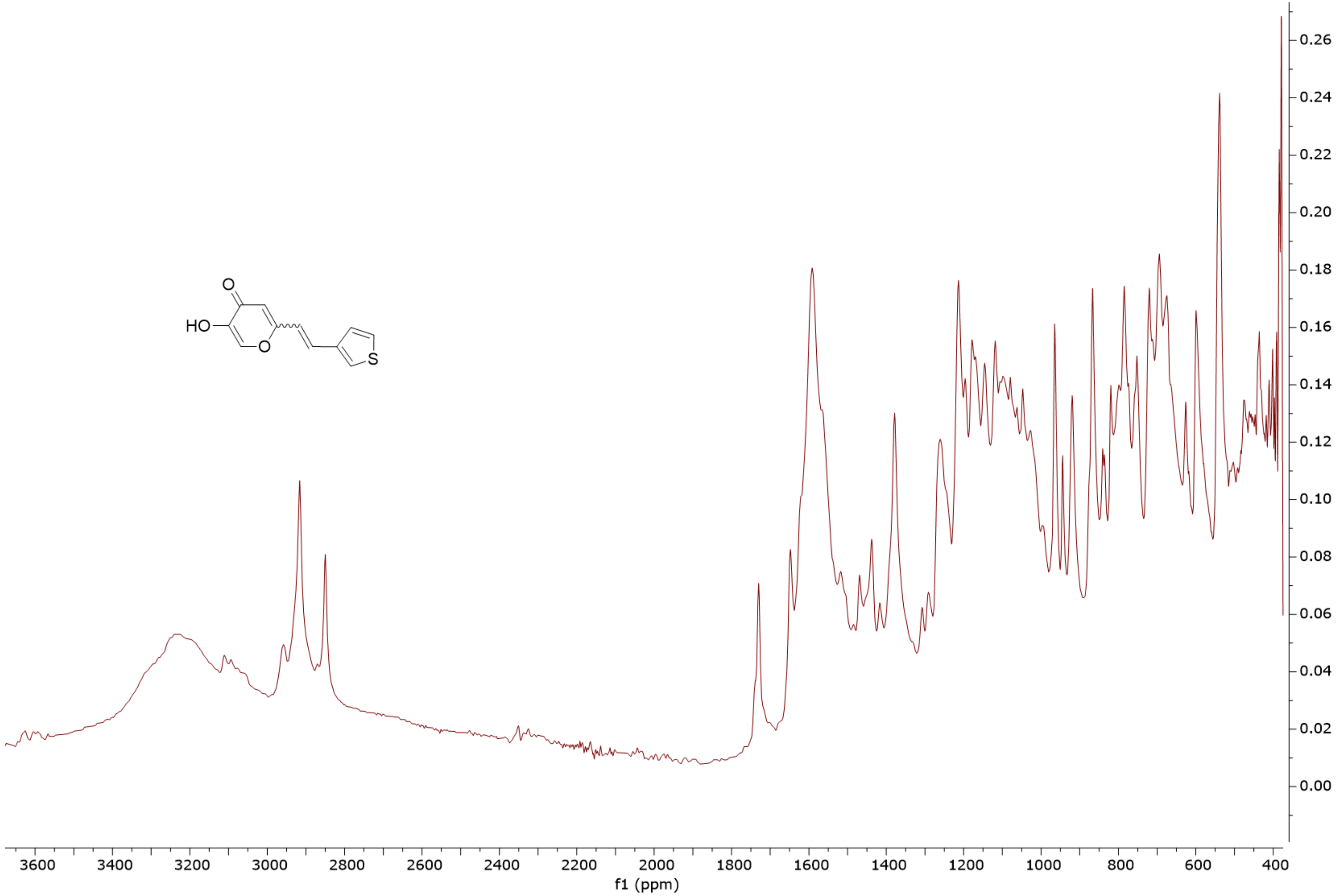

Figure S52. IR ATR spectrum of 5-hydroxy-2-(2-(thiophen-3-yl)vinyl)-4*H*-pyran-4-one (**6**).

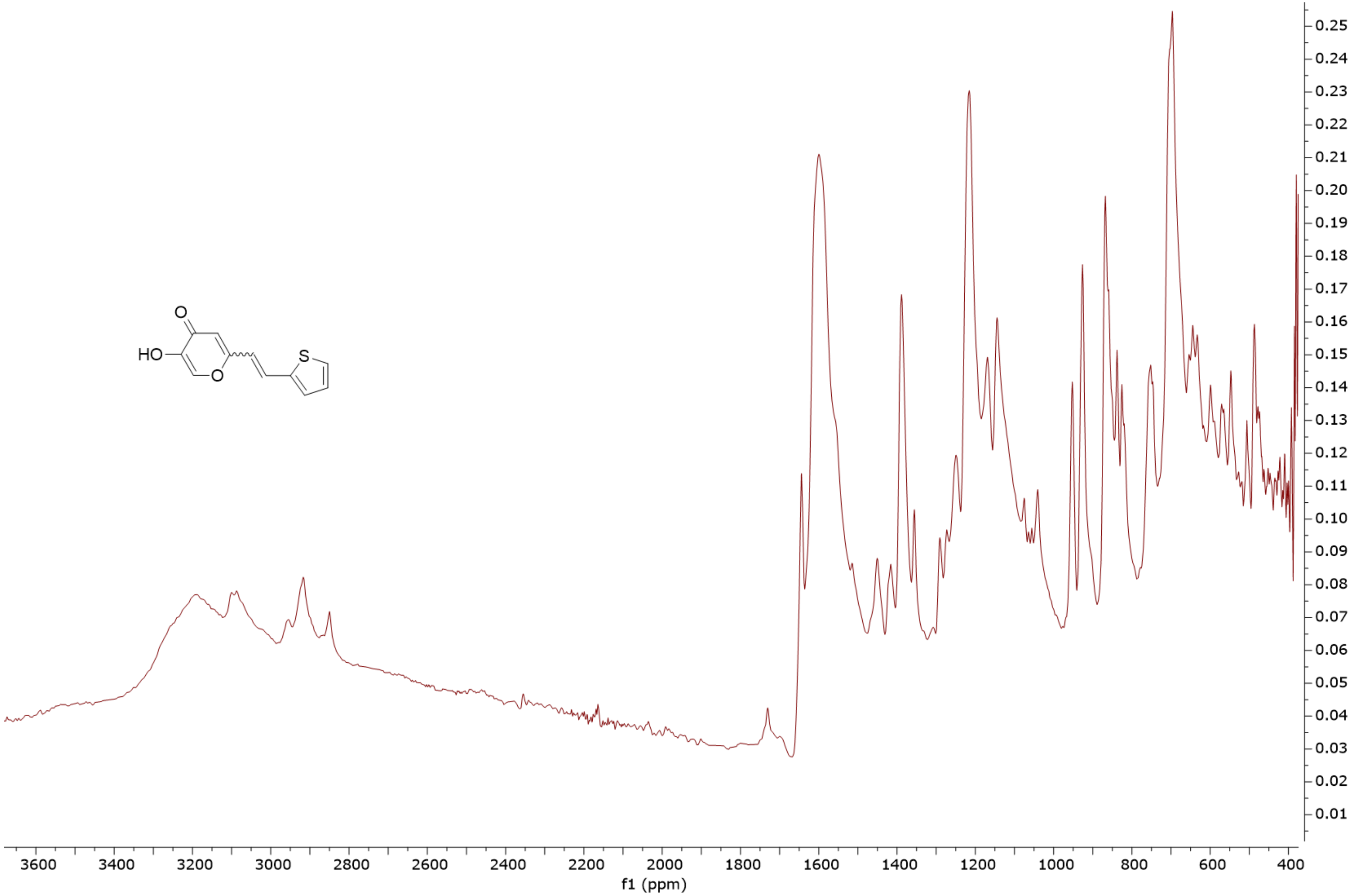

Figure S53. IR ATR spectrum of 5-hydroxy-2-(2-(thiophen-2-yl)vinyl)-4*H*-pyran-4-one (**7**).

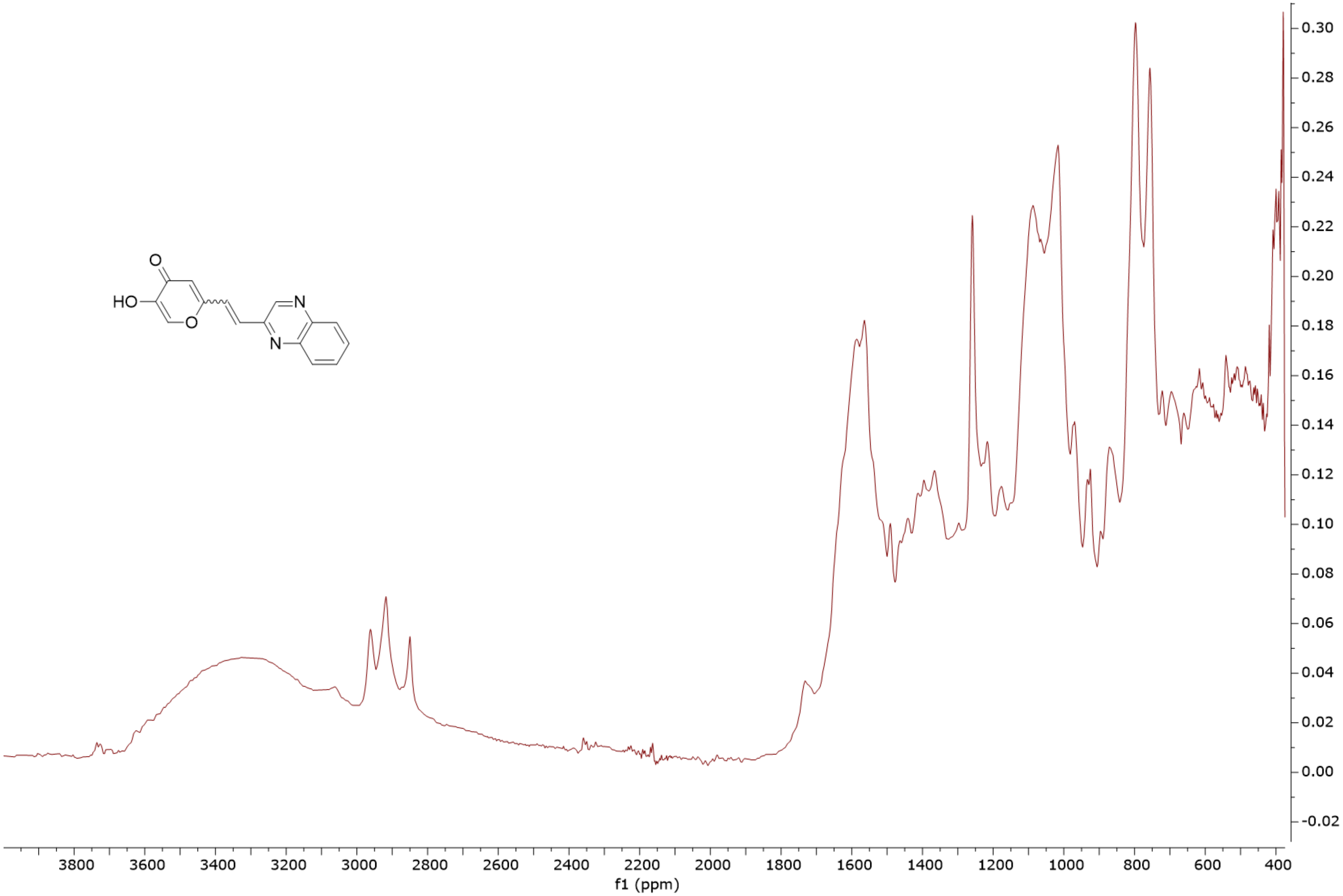

Figure S54. IR ATR spectrum of 5-hydroxy-2-(2-(quinoxalin-2-yl)vinyl)-4*H*-pyran-4-one (**8**).

4. HRMS analyses

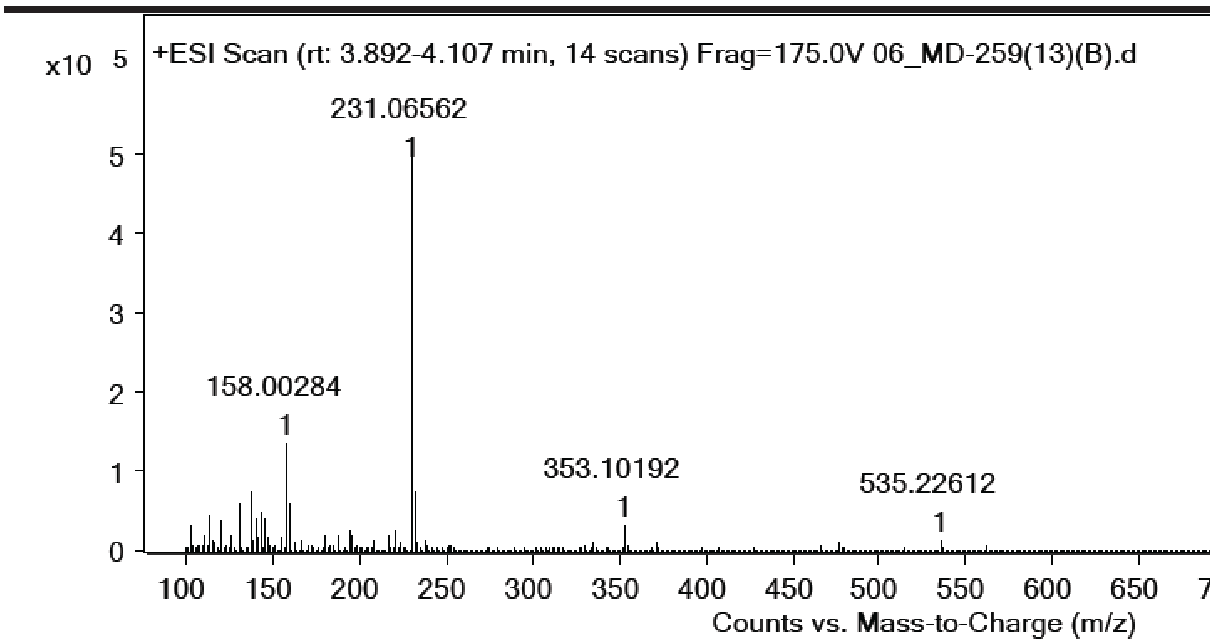

Formula Calculator Results

Formula	Best	Mass	Tgt Mass	Diff (ppm)	Ion Species	Score
C13 H10 O4	True	230.05833	230.05791	-1.83	C13 H11 O4	99.16

Figure S55. MS spectrum and HRMS analysis of 5-hydroxy-2-(2-hydroxystyryl)-4*H*-pyran-4-one (**1**).

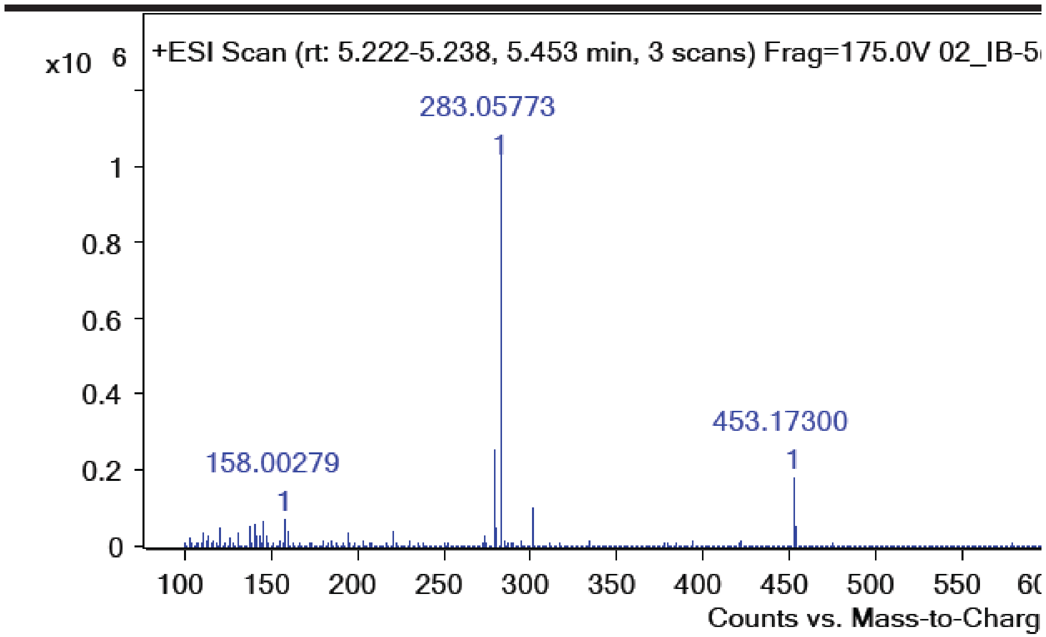

Formula Calculator Results

Formula	Best	Mass	Tgt Mass	Diff (ppm)	Ion Species	Score
C14 H9 F3 O3	True	282.0505	282.05038	-0.43	C14 H10 F3 O3	98.37

Figure S56. MS spectrum and HRMS analysis of 5-hydroxy-2-(2-(trifluoromethyl)styryl)-4*H*-pyran-4-one (**2**).

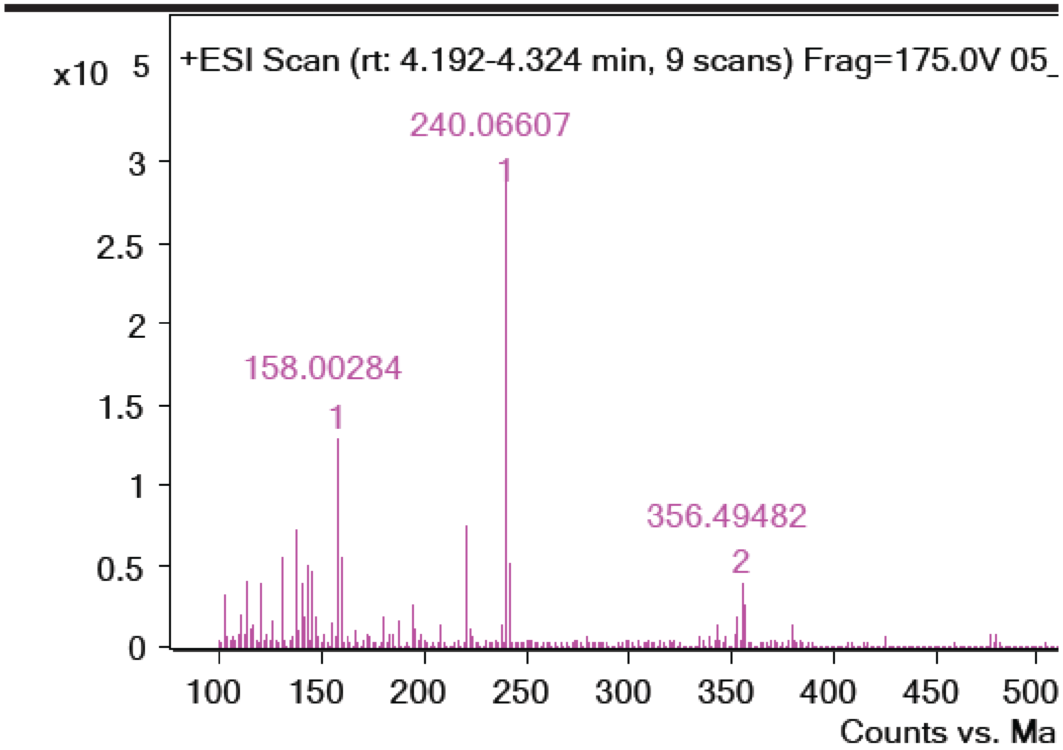

Formula Calculator Results

Formula	Best	Mass	Tgt Mass	Diff (ppm)	Ion Species	Score
C14 H9 N O3	True	239.05881	239.05824	-2.38	C14 H10 N O3	97.87

Figure S57. MS spectrum and HRMS analysis of 4-(2-(5-hydroxy-4-oxo-4*H*-pyran-2-yl)vinyl)benzonitrile (**3**).

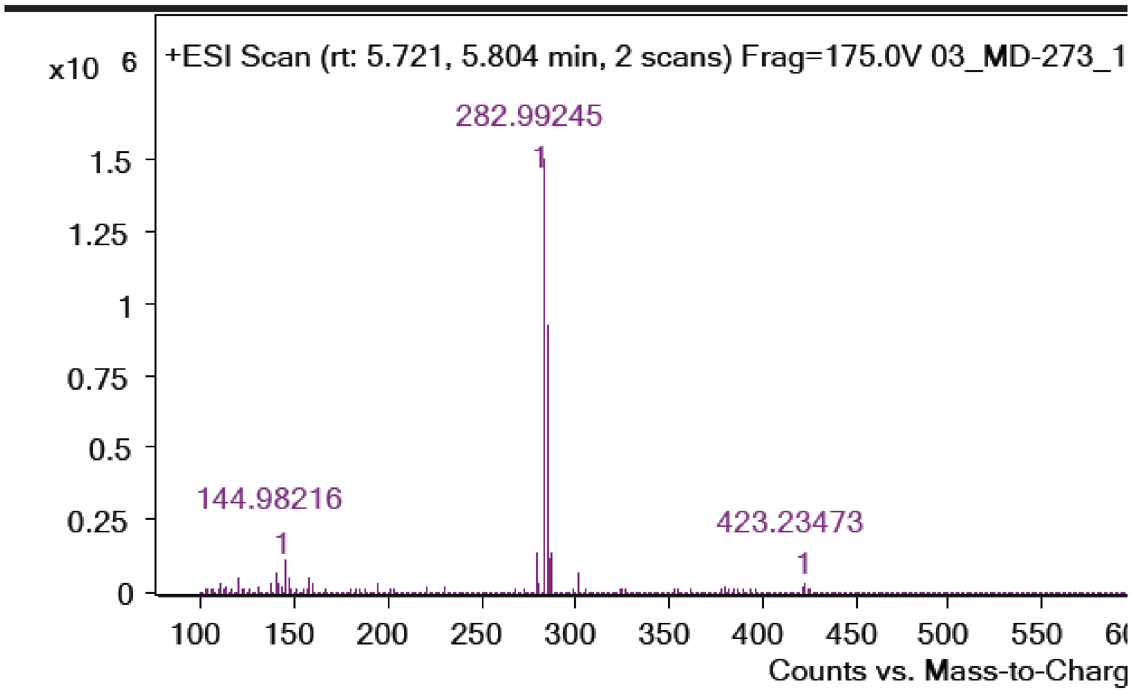

Formula Calculator Results

Formula	Best	Mass	Tgt Mass	Diff (ppm)	Ion Species	Score
C13 H8 Cl2 O3	True	281.98521	281.98505	-0.55	C13 H9 Cl2 O3	98.94

Figure S58. MS spectrum and HRMS analysis of 2-(2,4-dichlorostyryl)-5-hydroxy-4*H*-pyran-4-one (**4**).

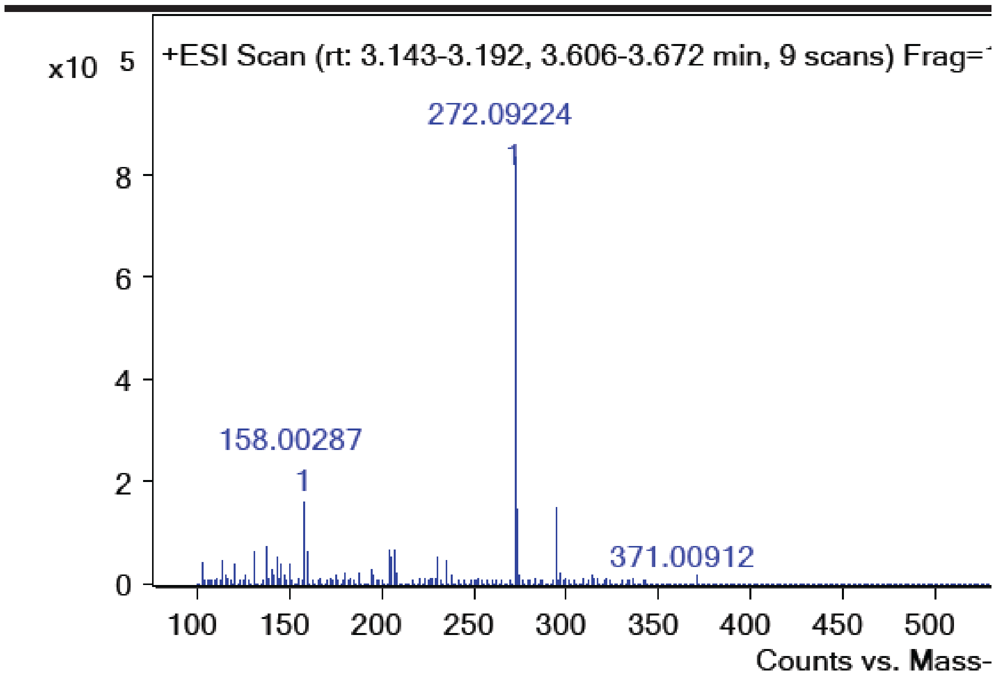

Formula Calculator Results

Formula	Best	Mass	Tgt Mass	Diff (ppm)	Ion Species	Score
C15 H13 N O4	True	271.08505	271.08446	-2.2	C15 H14 N O4	98.15

Figure S59. MS spectrum and HRMS analysis of *N*-(4-(2-(5-hydroxy-4-oxo-4*H*-pyran-2-yl)vinyl)phenyl)acetamide (**5**).

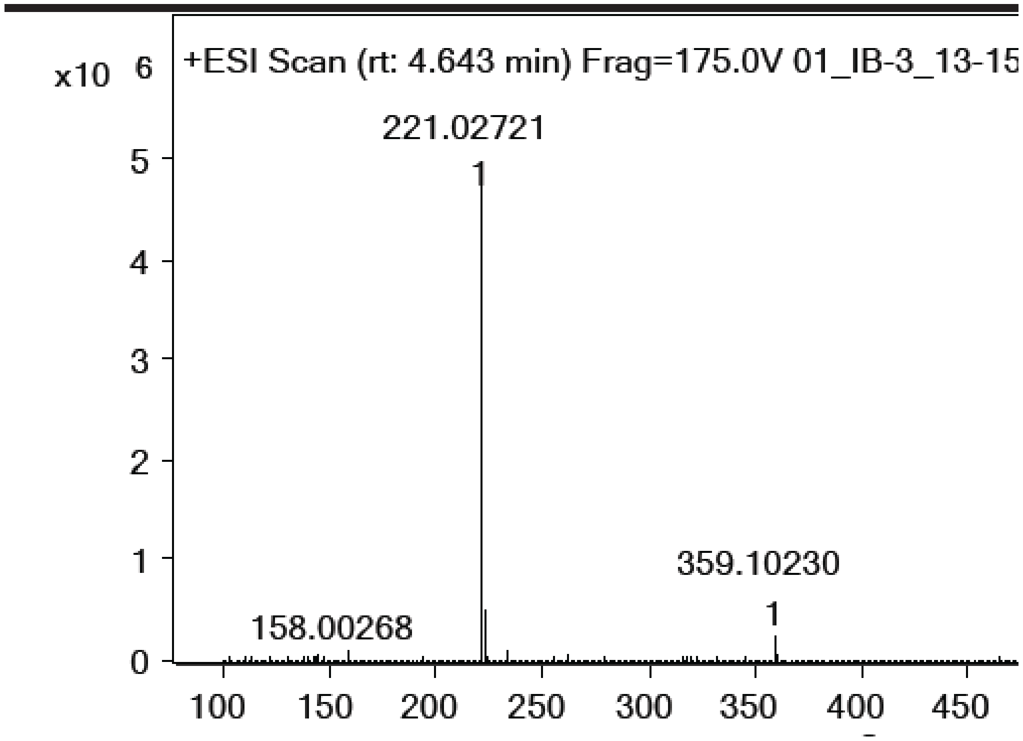

Formula Calculator Results

Formula	Best	Mass	Tgt Mass	Diff (ppm)	Ion Species	Score
C11 H8 O3 S	True	220.01998	220.01941	-2.55	C11 H9 O3 S	95.09

Figure S60. MS spectrum and HRMS analysis of 5-hydroxy-2-(2-(thiophen-3-yl)vinyl)-4*H*-pyran-4-one (**6**).

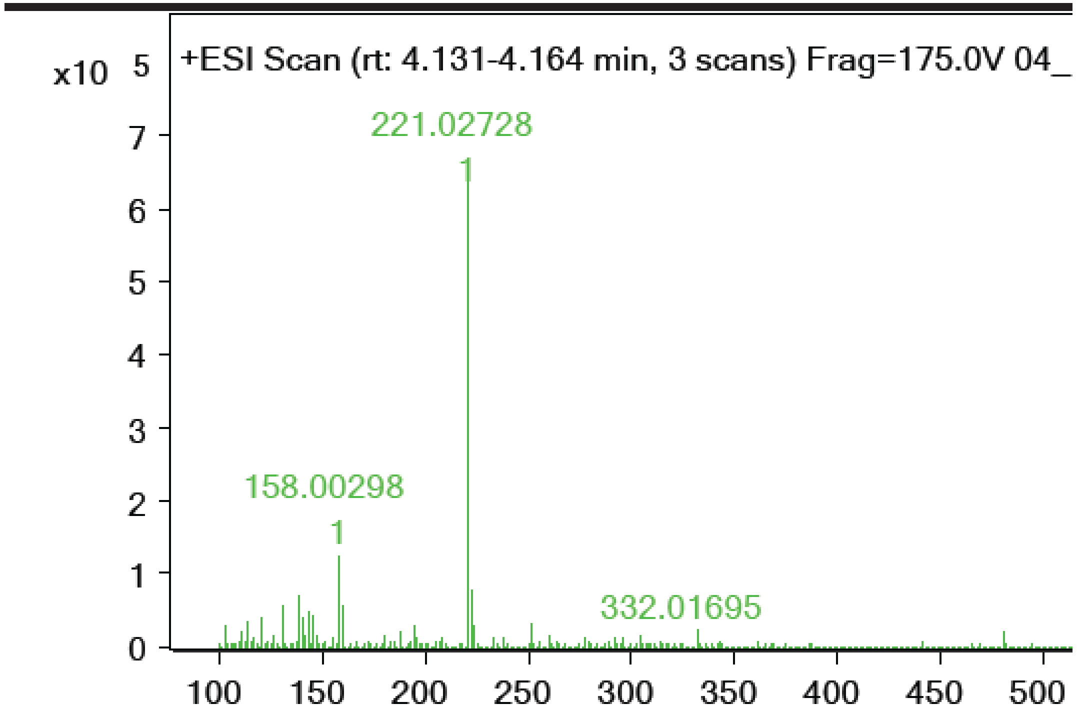

Formula Calculator Results

Formula	Best	Mass	Tgt Mass	Diff (ppm)	Ion Species	Score
C11 H8 O3 S	True	220.02002	220.01941	-2.75	C11 H9 O3 S	97.51

Figure S61. MS spectrum and HRMS analysis of 5-hydroxy-2-(2-(thiophen-2-yl)vinyl)-4*H*-pyran-4-one (**7**).

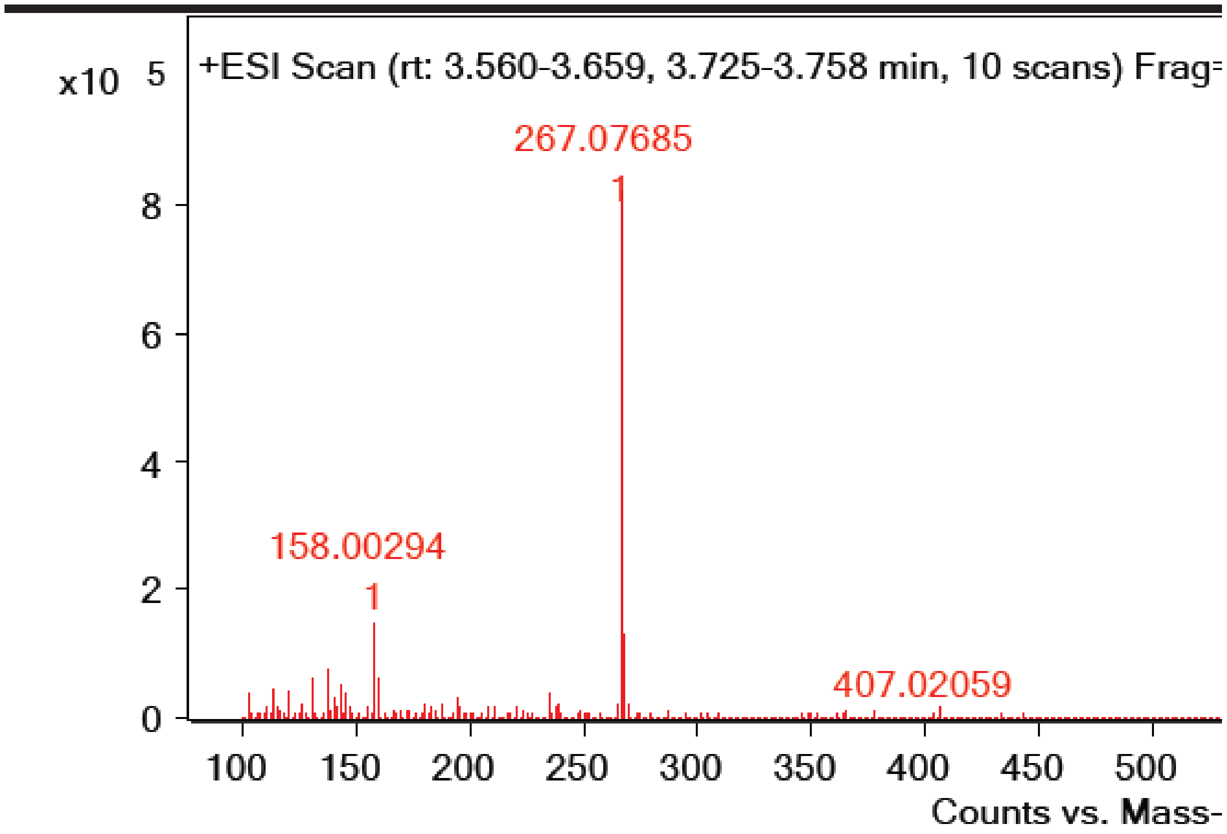

Formula Calculator Results

Formula	Best	Mass	Tgt Mass	Diff (ppm)	Ion Species	Score
C15 H8 N2 O3	True	264.05442	264.05349	-3.53	C15 H9 N2 O3	72.79
C15 H10 N2 O3	True	266.06973	266.06914	-2.19	C15 H11 N2 O3	96.65

Figure S62. MS spectrum and HRMS analysis of 5-hydroxy-2-(2-(quinoxalin-2-yl)vinyl)-4*H*-pyran-4-one (**8**).

5. Molecular docking

Table S1. Results of molecular docking of selected compounds into the active site of AChE (PDB ID: 1EEA). Free energies of binding given in kcal mol^{-1}, inhibition constants in μM.

Compound docked into AChE	Estimated free energies of binding (lowest/highest)		Estimated inhibition constants, K_i (lowest/highest)		Number of distinctive conformational clusters	Distribution of conformations within clusters
**II**	−5.35	−5.26	119	141	2	8, 17
**III**	−5.59	−5.38	80	114	2	24, 1
cis-**4**	−6.28	−6.04	25	38	4	13, 9, 2, 1

Table S2. Results of molecular docking of selected compounds into the active site of BChE (PDB ID: 1P0I). Free energies of binding given in kcal mol^{-1}, inhibition constants in μM.

Compound docked into BChE	Estimated free energies of binding (lowest/highest)		Estimated inhibition constants, K_i (lowest/highest)		Number of distinctive conformational clusters	Distribution of conformations within clusters
**II**	−5.04	−5.01	201	218	2	19, 6
**III**	−5.31	−5.12	129	179	2	23, 2
cis-**4**	−5.38	−5.19	113	157	3	15, 9, 1
trans-**6**	−4.61	−4.57	415	448	1	25

Table S3. Results of molecular docking of selected compounds into the active site of AChE (PDB ID: 1VOT, [1S]) in the presence of water molecules taken from Ref. 35. Free energies of binding given in kcal mol^{-1}, inhibition constants in μM.

Compound docked into AChE	Estimated free energies of binding (lowest/highest)		Estimated inhibition constants, K_i (lowest/highest)		Number of distinctive conformational clusters	Distribution of conformations within clusters
**II**	−5.94	−5.57	45	83	6	17, 2, 3, 1, 1, 1
**III**	−6.19	−6.09	29	63	1	25
cis-**4**	−7.10	−6.14	6	32	4	8, 9, 7, 1

Table S4. Results of molecular docking of selected compounds into the active site of BChE (PDB ID: 5DYW, [2S]) in the presence of five structural water molecules, according to Ref 36. Free energies of binding given in kcal mol^{-1}, inhibition constants in μM.

Compound docked into BChE	Estimated free energies of binding (lowest/highest)		Estimated inhibition constants, K_i (lowest/highest)		Number of distinctive conformational clusters	Distribution of conformations within clusters
**II**	−4.75	−4.71	330	404	2	11, 14
**III**	−5.12	−4.55	175	458	2	22, 3
cis-**4**	−5.54	−4.81	87	302	2	22, 3
trans-**6**	−4.46	−4.10	537	995	2	24, 1

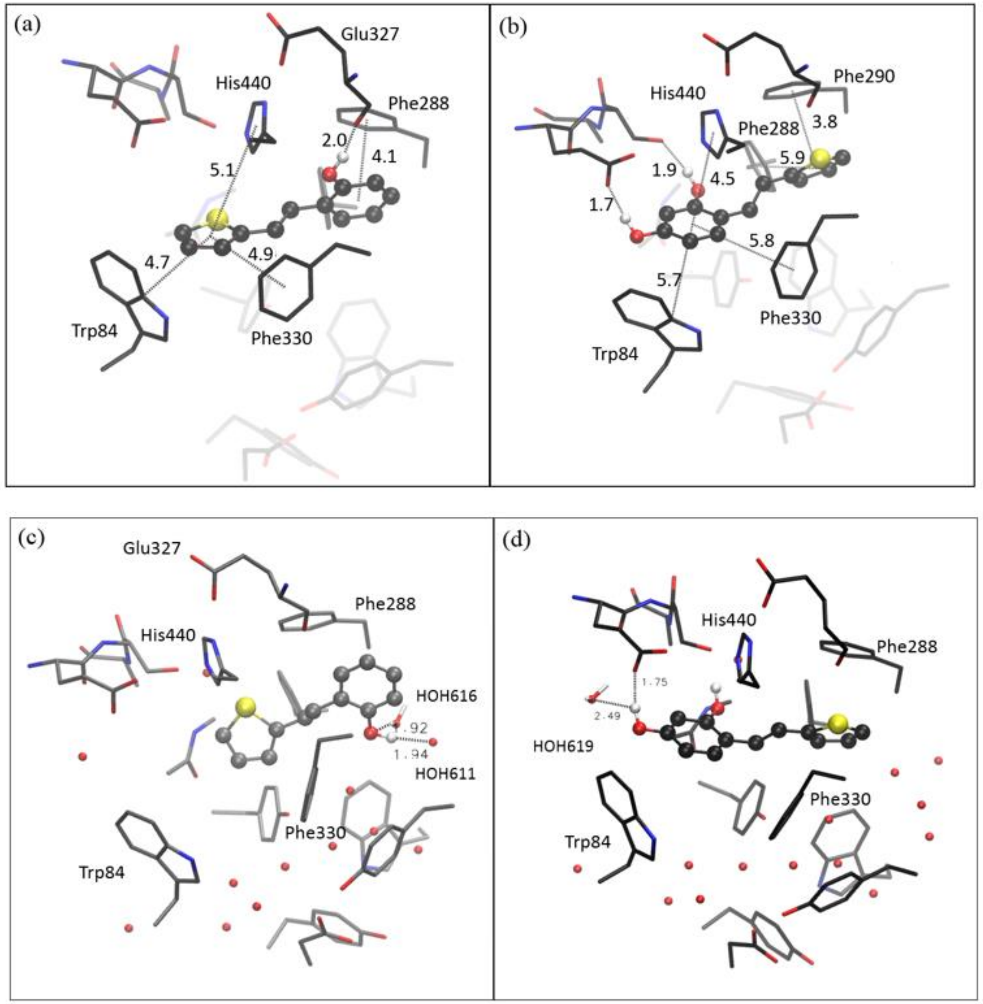

Figure S63. The structures of the active site of AChE (PDB ID: 1EEA) docked with **II** (a) and **III** (b), and the active site of AChE (PDB ID: 1VOT) in the presence of water molecules (Ref. 35) docked with **II** (c) and **III** (d). Figures (a) and (b) are also presented in the main text (Fig. 8); here are given for comparison. Hydrogens omitted for the sake of clarity.

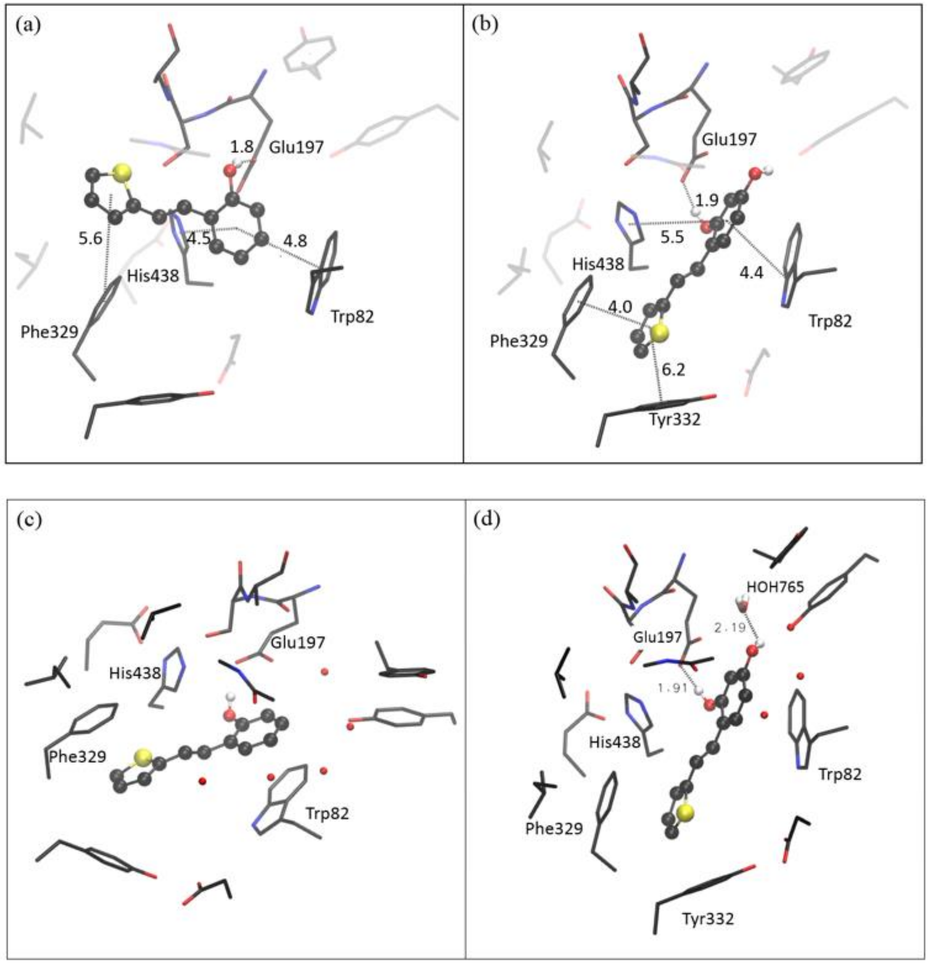

Figure S64. The structure of the active site of BChE (PDB ID: 1P0I) docked with **II** (a) and **III** (b), and the active site of BChE (PDB ID: 5DYW) in the presence of five structural water molecules (Ref. 36) docked with **II** (c) and **III** (d). Figures (a) and (b) are also presented in the main text (Fig. 9); here are given for comparison. Hydrogens omitted for the sake of clarity.

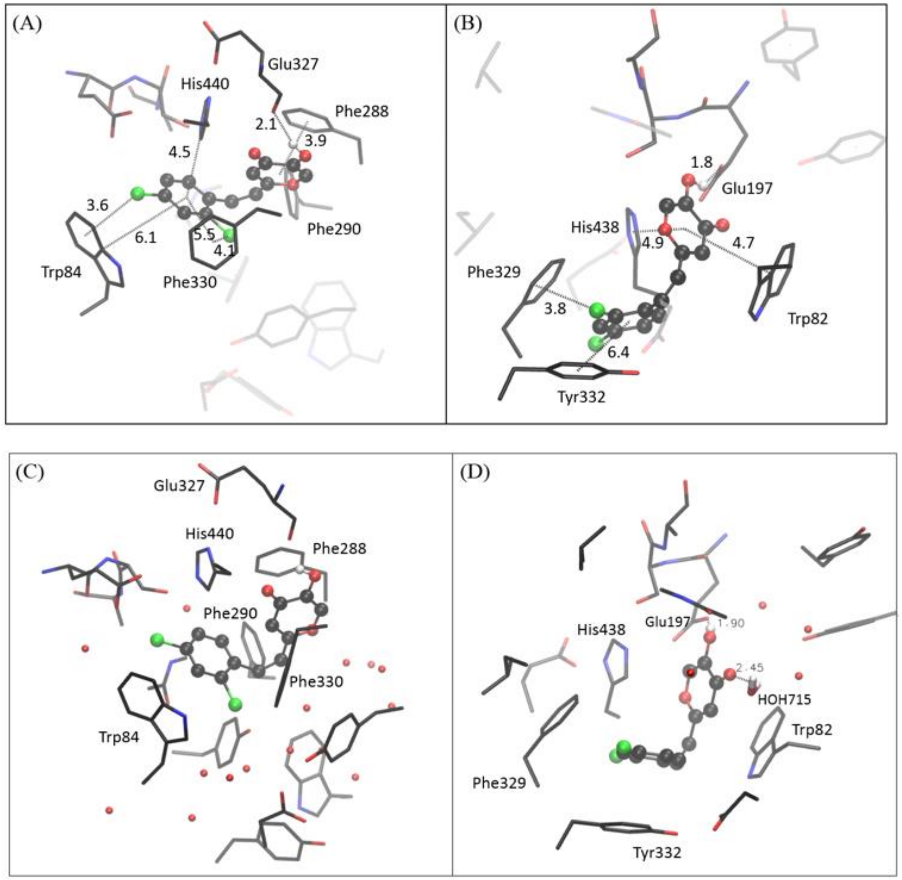

Figure S65. The active sites of AChE (PDB ID: 1EEA) and BChE (PDB ID: 1P0I) docked with *cis*-**4** (A and B), and the active site of AChE (PDB ID: 1VOT) with molecules of water (Ref 35) and BChE (PDB ID: 5DYW) in the presence of five structural water molecules (Ref. 36) docked with *cis*-**4** (C and D, respectively). Figures (A) and (B) are also presented in the main text (Fig. 10); here are given for comparison. Hydrogens omitted for the sake of clarity.

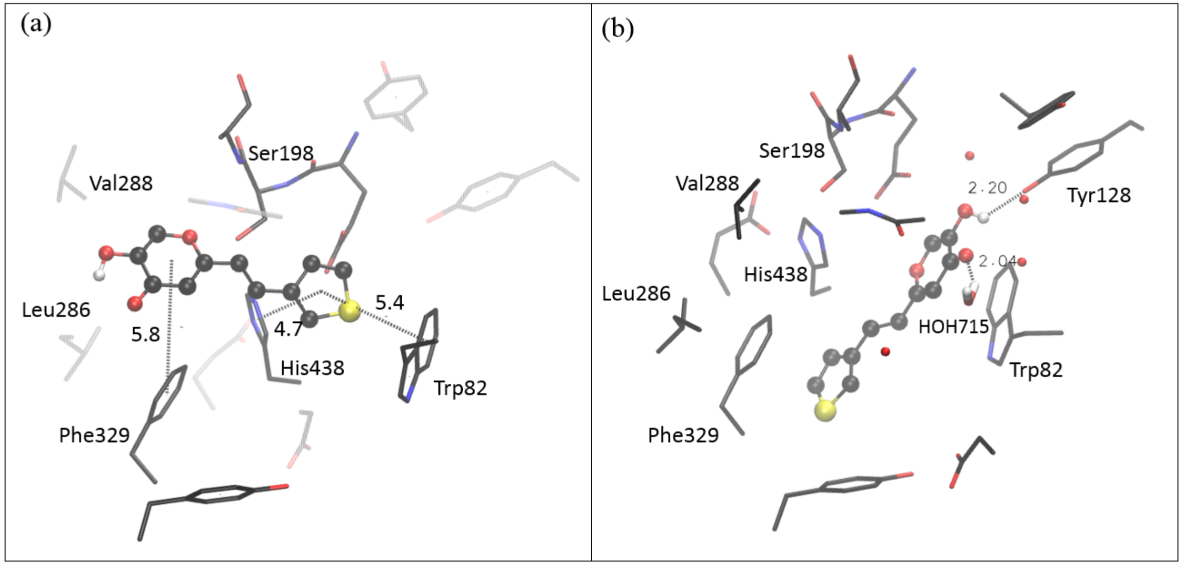

Figure S66. Compound *trans*-**6** docked into the active site of BChE (1P0I.pdb) (a), and into the active site of BChE (1DYW.pdb) in the presence of five structural water molecules (Ref. 36). Figure (a) is also presented in the main text (Fig. 11); here given for comparison. Hydrogens omitted for clarity.

6. Biometal chelating capability of cholinesterase inhibitory active pyranones

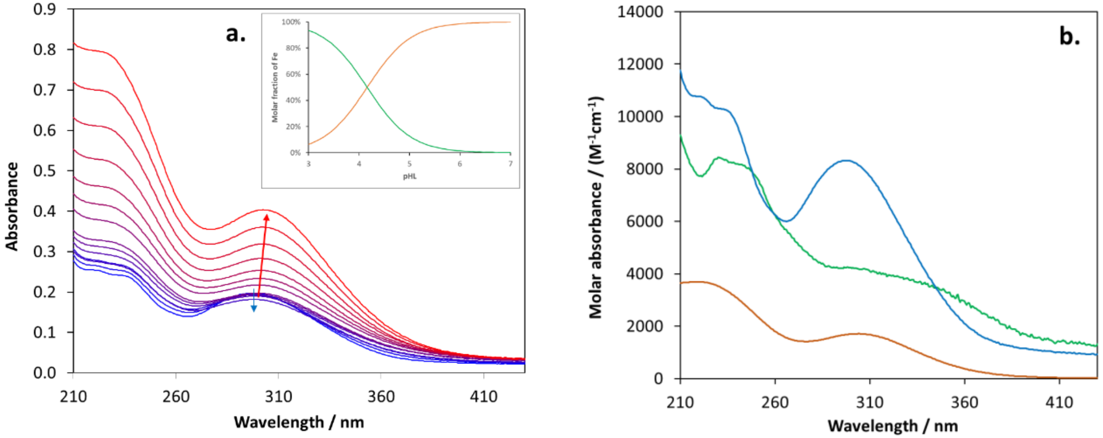

Figure S67. The measured absorption spectra (a.) at constant total ligand (**cis-2**) concentration ($5.4{\cdot}10^{-5}$ M) and various Fe^{3+} total concentration (from 0 M (blue) to $1.1{\cdot}10^{-4}$ M (red)). The inset shows the molar fraction of free Fe^{3+} (orange) and 1:1 complex (FeL^{2+}) (green) as functions of the protonated ligand concentration (pHL). Figure b shows the molar absorbance of Fe^{3+} (orange), ligand (blue), and FeL^{2+} adduct (green).

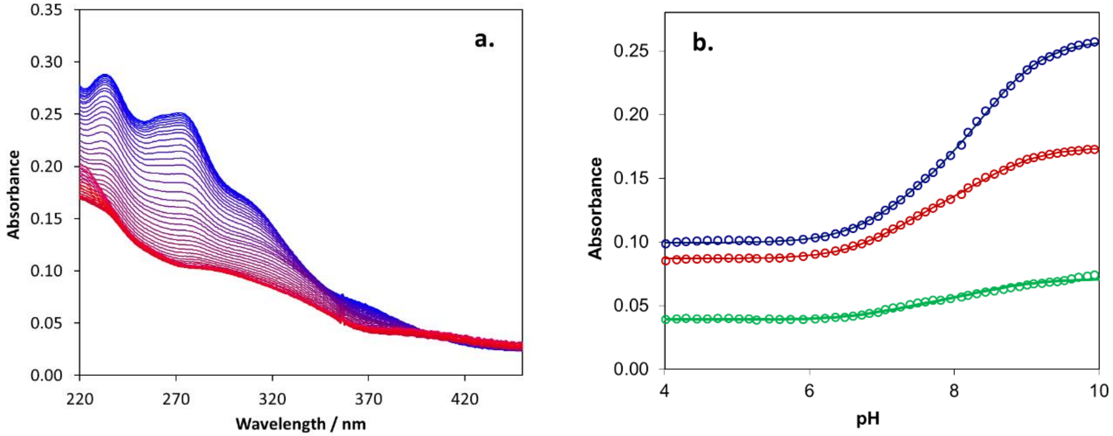

Figure S68. The measured absorbance spectra (a.) during potentiometric titration of ligand (**cis-2**). The pH changes from 10.0 (blue) to 6.0 (red). Figure b shows the measured (circles) and the calculated absorbance (lines) at two characteristic wavelengths (258 nm (blue), and 365 nm (red)).

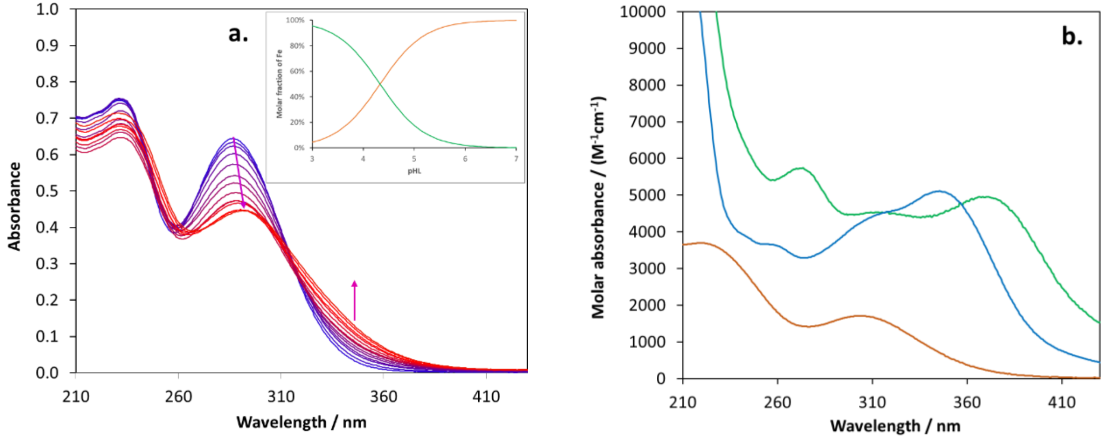

Figure S69. The measured absorbance spectra (a.) at constant total ligand (**cis-4**) concentration ($2.4 \cdot 10^{-5}$ M) and various Fe^{3+} total concentration (from 0 M (blue) to $1.8 \cdot 10^{-4}$ M (red)). The inset shows the molar fraction of free Fe^{3+} (orange) and 1:1 complex (FeL^{2+}) (green) as functions of the protonated ligand concentration (pHL). Figure b shows the molar absorbance of Fe^{3+} (orange), ligand (blue), and FeL^{2+} adduct (green).

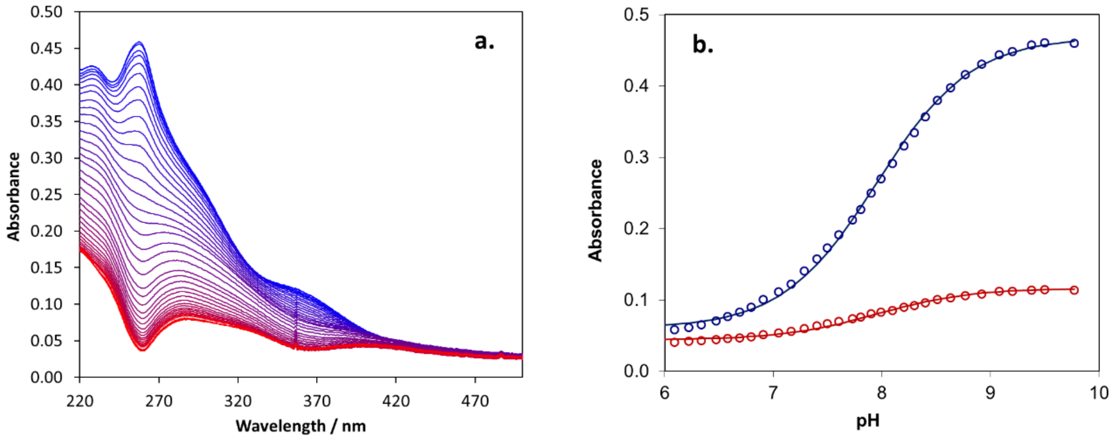

Figure S70. The measured absorbance spectra (a.) during potentiometric titration of ligand (**cis-4**). The pH changes from 10.0 (blue) to 4.0 (red). Figure b shows the measured (circles) and the calculated absorbance (lines) at three characteristic wavelengths (271 nm (blue), 308 nm (red), and 368 nm (green)).

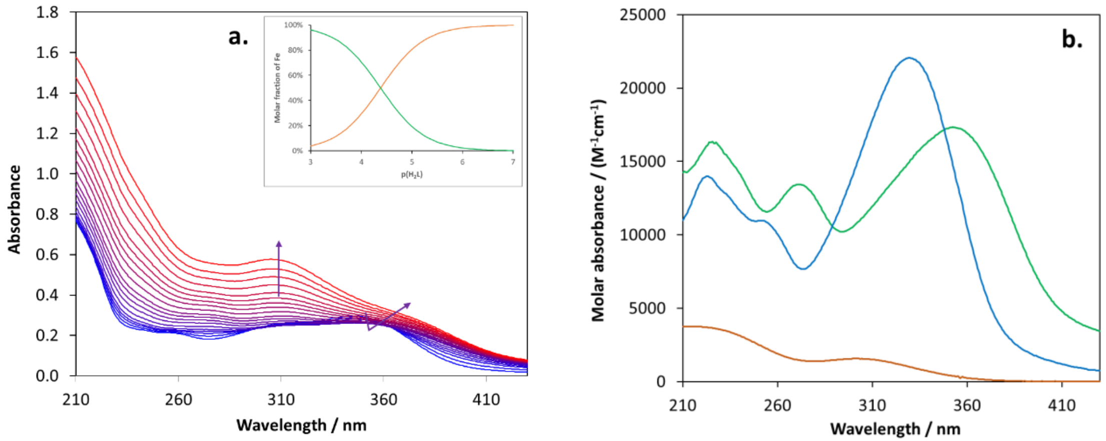

Figure S71. The measured absorbance spectra (a.) at constant total ligand (**trans-1**) concentration (5.5·10⁻⁵ M) and various Fe^{3+} total concentration (from 0 M (blue) to 2.4·10⁻⁴ M (red)). The inset shows the molar fraction of free Fe^{3+} (orange) and 1:1 complex ($FeHL^{2+}$) (green) as functions of the protonated ligand concentration (pH_2L). Figure b shows the molar absorbance of Fe^{3+} (orange), ligand (blue), and $FeHL^{2+}$ adduct (green).

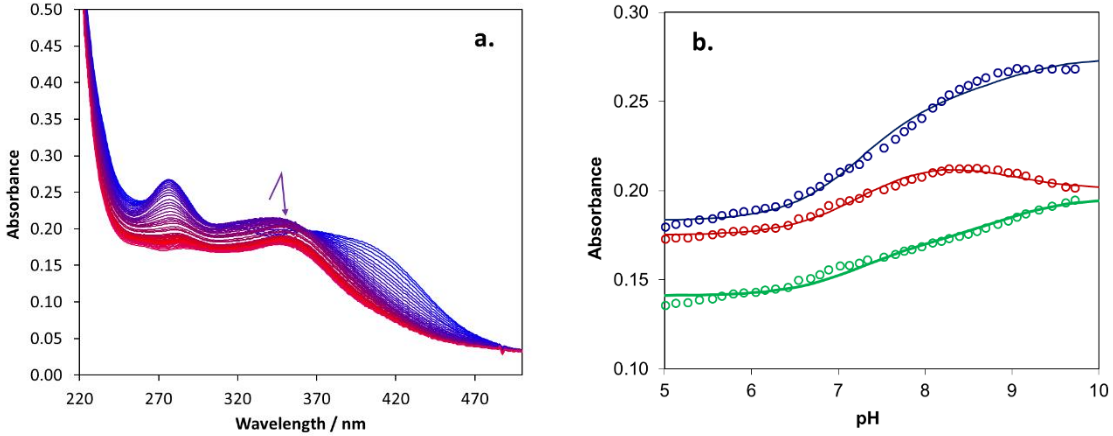

Figure S72. The measured absorbance spectra (a.) during potentiometric titration of ligand (**trans-1**). The pH changes from 10.0 (blue) to 5.0 (red). Figure b shows the measured (circles) and the calculated absorbance (lines) at three characteristic wavelengths (276 nm (blue), 346 nm (red), and 410 nm (green)).

7. Crystal structures and packing of resveratrol-maltol hybrids

Table S5. Geometric parameters of hydrogen bonds determined from geometric analysis.

	D–H / Å	H···*A* / Å	*D*···*A* / Å	*D*–H···*A* / º	Symm. op. on *A*
cis-**2**					
O2–H2A···O1	0.82	2.37	2.782(4)	112	*x*, *y*, *z*
O2–H2A···O1	0.82	1.96	2.689(4)	147	1–*x*, 2–*y*, –*z*
C2–H2···F3	0.93	2.37	2.709(7)	101	*x*, *y*, *z*
C7–H7···F1	0.93	2.67	3.270(5)	123	*x*, –1+*y*, *z*
trans-**6**					
O3–H3···O2	0.82	2.41	2.788(4)	109	*x*, *y*, *z*
O3–H3···O2	0.82	1.95	2.636(4)	140	–*x*, 2–*y*, 1–*z*
O6–H6A···O5	0.82	2.34	2.762(5)	113	*x*, *y*, *z*
O6–H6A···O5	0.82	1.99	2.712(5)	147	1–*x*, –1–*y*, 1–*z*
C4–H4···O5	0.93	2.54	3.224(6)	130	*x*, *y*, *z*
C5–H5···O1	0.93	2.43	2.753(5)	101	*x*, *y*, *z*
C13–H13···O3	0.93	2.44	3.320(5)	158	1+*x*, *y*, *z*
C16–H16···O4	0.93	2.38	2.729(5)	102	*x*, *y*, *z*
C22–H22···O2	0.93	2.57	3.300(5)	136	1–*x*, 1–*y*, 1–*z*

Table S6. Geometric parameters of the C–H···π interactions.

C–H···π	H···Cg / Å	γ^a / º	C–H···Cg / Å	C···Cg / Å	Symm. Operation on Cg
cis-**2**					
C5–H5···O3→C13	2.84	12.65	164	3.745(5)	*x*, –1+*y*, *z*
trans-**6**					
C1–H1···S1→C4	2.83	19.75	141	3.606(5)	1–*x*, –1/2+*y*, 3/2–*z*
C12–H12···S2→C15	2.96	22.70	145	3.757(3)	2–*x*, 1/2+*y*, 3/2–*z*

[a] γ = angle defined by a line connecting centre of gravity of the aromatic ring with H atom and the normal to the aromatic ring.

Table S7. Geometric parameters of π interactions in the compound *trans*-**6**.

$\pi\cdots\pi$	$Cg^a\cdots Cg$ / Å	α^b / °	β^c / °	Cg···plane(Cg2) / Å	Offset/ Å	Symm.
S1→C4···S1→C4	5.148(2)	43.5	43.5	3.7347(17)	3.543	*x*, –1+*y*, *z*
S1→C4···S1→C4	4.771(2)	17.7	80.2	4.5454(17)	-	1–*x*, 1/2+*y*, 3/2–*z*
S1→C4···O1→C11	4.801(2)	35.8	48.7	3.1710(18)	-	*x*, –1+*y*, *z*
O1→C11···O1→C11	5.148(2)	51.4	51.4	3.2109(15)	4.024	*x*, –1+*y*, *z*
O1→C11···O1→C11	4.739(2)	46.0	46.0	3.2901(15)	3.411	–*x*, 1–*y*, 1–*z*
O1→C11···S2→C15	5.877(2)	57.5	68.1	2.1958(15)	-	–1+*x*, *y*, *z*
O1→C11···S2→C15	5.496(2)	46.3	68.4	2.0221(15)	-	1–*x*, –1/2+*y*, 3/2–*z*
S2→C15···S2→C15	5.148(2)	45.6	45.6	3.5990(17)	3.681	*x*, –1+*y*, *z*
S2→C15···S2→C15	5.008(2)	16.8	76.2	4.7939(17)	-	2–*x*, –1/2+*y*, 3/2–*z*
S2→C15···O4→C22	4.459(2)	39.2	35.8	3.6143(17)	2.817	1–*x*, 1/2+*y*, 3/2–*z*
O4→C22···O4→C22	5.148(2)	47.0	47.0	3.5123(16)	3.763	*x*, –1+*y*, *z*

References in Supporting Information

1S. Košak, U.; Brus, B.; Knez, D.; Šink, R.; Žakel, S.; Trontelj, J.; Pišlar, A.; Šlenc, J.; Gobec, M.; Živin, M.; et al. Development of an in-vivo active reversible butyrylcholinesterase inhibitor. *Sci. Rep.* **2016**, *6*, 39495.

2S. Raves, M.L.; Harel, M.; Pang, Y.P.; Silman, I.; Kozikowski, A.P.; Sussman, J.L. Structure of acetylcholinesterase complexed with the nootropic alkaloid, (-)-huperzine A. *Nat. Struct. Biol.* **1997**, *4*, 57.
